# Liberal-conservative asymmetries in anti-democratic tendencies are partly explained by psychological differences in a nationally representative U.S. sample

**DOI:** 10.1038/s44271-024-00096-3

**Published:** 2024-07-02

**Authors:** Débora de Oliveira Santos, John T. Jost

**Affiliations:** 1 Department of Political Science, Federal University of Rio Grande do Sul, Porto Alegre, RS Brazil; 2 Department of Psychology, New York University, New York, NY USA

**Keywords:** Psychology, Politics

## Abstract

Based on theory and research in political psychology, we hypothesized that liberal-conservative differences in right-wing authoritarianism, social dominance orientation, and political system justification would contribute to asymmetries in anti-democratic tendencies. These hypotheses were tested in a nationally representative survey of U.S. adults (*N* = 1557). Results revealed that conservatives were less supportive of political equality and legal rights and guarantees and more willing to defect from democratic “rules of the game” and vote for anti-democratic candidates, even after adjusting for political extremism. Mediational analyses suggested that conservatives’ anti-democratic tendencies were partially attributable to higher levels of right-wing authoritarianism and social dominance orientation. Conservatives also scored higher in political system justification, which was associated with support for free speech and mitigated anti-democratic tendencies. Democrats and Republicans who approved January 6, 2021, insurrectionists were more conservative and higher in right-wing authoritarianism than those who did not. Implications for social psychology and society are discussed.

## Abstract (web summary)

In a nationally representative survey in the United States, conservatives were less supportive of democratic norms such as political equality and legal rights and guarantees as compared to liberals. These associations were partially mediated by higher levels of right-wing authoritarianism and social dominance orientation.

## Introduction

Increasingly, social scientists have sounded the alarm that Western democracy is at risk^1–7^. Events such as the violent attack on the U.S. Capitol by “Stop the Steal” supporters of Donald Trump on January 6, 2021^8^, and a similar insurrection by supporters of Jair Bolsonaro in Brazil on January 8, 2023^9^, suggest to many that political polarization in several countries has reached dangerous levels, threatening the peaceful transfer of power and other democratic norms and practices.

Experts agree that some degree of political polarization is useful in democratic societies to ensure electoral choice^10–12^. However, extremely high levels of divisiveness and animosity can be harmful. Polarization at the level of elite politicians may prevent compromise, create gridlock, and contribute to majoritarian drift, single-party dominance, and the politicization of democratic norms^1,5,6,13,14^. At the mass level, extreme polarization may undermine trust in and support for democracy and lead people to engage in political violence and other anti-democratic activities^3,4,7,15–19^. Over time, pernicious forms of political polarization may threaten both the legitimacy and stability of liberal democracy.

The U.S. is an especially important context to understand, insofar as elite- and mass-level support for democracy has long been taken for granted^20–22^. Some scholars continue to assert that, while political elites are divided over issues of policy, ordinary citizens in the U.S. are not especially polarized^23,24^. Others, however, find the evidence for mass polarization to be convincing and possibly troubling^2,25–27^. Even when questioned about whether the attack on the U.S. Capitol on January 6, 2021, was a legitimate form of protest, Democrats and Republicans are deeply divided^28^. As evidence of increasing polarization among elites and ordinary citizens has accumulated, expert reports conclude that the quality of democracy in the U.S. has declined rapidly^29,30^.

Most tools of political science, which emphasize institutional factors and partisan dynamics in the context of mutual electoral competition, lend themselves to symmetrical analyses of political polarization and defection from democratic norms^14,16,18,31,32^. The same is true of prominent social psychological theories that focus on generic processes of group identification and in-group favoritism^33–38^. Thus, it is often assumed *a priori* that “both sides” (liberals and conservatives, Democrats and Republicans) are equally responsible for the deterioration of intergroup relations and defection from democratic norms of tolerance and civility in the U.S.

At the same time, a sizeable literature in political psychology documents significant asymmetries between the liberal-left and conservative-right that have clear ramifications for democratic commitment^39^. To begin with, conservatives score higher than liberals on individual difference measures of right-wing authoritarianism (RWA) and social dominance orientation (SDO)^40–45^. For instance, conservatives are more likely than liberals to endorse statements such as the following: “Some groups of people are simply inferior to other groups”; “What our country needs instead of more ‘civil rights’ is a good stiff dose of law and order”^46^. RWA and SDO are both associated with decreased commitment to basic democratic norms and principles^47–52^. Prior research finds that higher levels of RWA and SDO are linked to support for restrictions on civil liberties and human rights^48,50^, willingness to support repressive governmental measures^48^, and lesser endorsement of democratic values and the democratic system^49,50,52^.

In the present research program, we investigated the possibility that there would be left-right ideological asymmetries in anti-democratic tendencies in the U.S., and that these asymmetries would be explained, at least in part, by psychological differences. Specifically, we hypothesized that political conservatism would be positively associated with anti-democratic tendencies and that this direct effect would be statistically mediated by indirect effects of RWA (H1) and SDO (H2). We measured pro- vs. anti-democratic sentiment with seven different attitudes about (a) rights and guarantees according to the democratic rule of law; (b) political equality in voting; (c) freedom of speech; (d) defection from the democratic “rules of the game”; (e) willingness to use political violence; (f) tolerance of disliked groups; and (g) willingness to vote for blatantly anti-democratic candidates.

Individuals also differ in terms of political system justification (PSJ), that is, the extent to which they subscribe to the legitimacy and desirability of the existing political system^53^, and these differences predict support for the political status quo^54^. Surveys conducted in France, Germany, and the U.K. revealed that individuals who scored higher on PSJ were more supportive of traditional, mainstream political parties and less supportive of radical, anti-establishment parties led by left-wing or right-wing populists^55^. Therefore, we expected that PSJ would be related to support for the democratic status quo. Because political conservatism is positively associated with system justification in the U.S.^53^, we hypothesized that—after adjusting for RWA and SDO— the indirect effect of conservatism on support for anti-democratic activity via PSJ would be negative in sign (H3).

Finally, we hypothesized that there would be meaningful psychological differences between Americans who approved (vs. disapproved) of the January 6, 2021, insurrectionists. Specifically, we predicted that both Democrats and Republicans who approved of the insurrectionists would be higher in RWA (H4) and SDO (H5) and lower in PSJ (H6), compared to fellow co-partisans who disapproved of the insurrectionists. To further probe liberal-conservative asymmetries in anti-democratic tendencies we included additional models in which we adjusted for both ideological and partisan extremity. All the above hypotheses were tested in a nationally representative survey of U.S. adults (*N* = 1557).

## Methods

### Data

All data used in this article come from the 2022 Health of Democracy Survey, commissioned by the University of Notre Dame and carried out by the National Opinion Research Center (NORC) at the University of Chicago in connection with the AmeriSpeak® Panel and following AAPOR’s Transparency Initiative. Initially, 6509 panelists already registered on the NORC’s AmeriSpeak® Panel – which recruitment is based on household level – were selected and invited to participate in the 2022 Health of Democracy Survey. The sample of panelists was selected using sampling strata based on age, race/ethnicity, education, and sex. Sampled panelists received an initial invitation from NORC and email reminders to take the survey online through the AmeriSpeak® Mobile App, the password-protected AmeriSpeak® Web portal, or by following a link in the email invitation. To incentivize participation, sampled panelists were offered $4.00 for completing the survey. From the 6509 sampled panelists, the final survey sample was nationally representative of the U.S. general adult population above 18 years old and composed of 1557 respondents who accepted and completed the survey (and after removing 78 observations that failed to satisfy data quality parameters). The survey was self-administered, online, and available in English and Spanish, and it was fielded from October 18–26, 2022. The study was not preregistered.

### Analytic strategy

After identifying batteries of items to assess pro- and anti-democratic sentiment (dependent variables), ideological orientations (independent variables), and psychological dispositions (independent and mediating variables), we factor-analyzed the items in each battery and created new variables based on factor scores. Variables were formally tested for normality and equal variance. We used SPSS 25 to run exploratory principal-axis factor analyses with the oblimin rotation method. Factor scores were then transformed into new variables using regression.

Next, we performed hierarchical regression models estimating robust standard errors to account for heteroscedasticity for each dependent variable derived from the factor analysis using R 4.2.2. Hierarchical regression models were specified as follows: Step 1: demographic variables (race, ethnicity, age, sex, income, and education); Step 2: ideological variables (conservative [vs. liberal] ideological self-placement and strength of Republican [vs. Democratic] Party identification); Step 3: demographic and ideological variables included in Steps 1 and 2; Step 4: demographic, ideological, and extremism (ideological and partisan extremism) variables; Step 5: demographic, ideological, and psychological (RWA, SDO, and PSJ) variables; and Step 6: all variables included in Steps 4 and 5. We also performed a Structural Equation Model to test the mediation role of psychological differences in pro- and anti-democratic tendencies with R 4.2.2, using the “lavaan” package version 0.6-15^56^ with 10,000 bootstraps. In addition, to explore the ideological and psychological differences between Republicans and Democrats who expressed warm feelings toward January 6 and their partisan peers who did not, we conducted Mann-Whitney-Wilcoxon tests.

### Measures

Details concerning scale items and internal consistencies for variables used in the analyses are provided in Supplementary Methods (Supplementary Table 1).

#### Ideological self-placement

Respondents were asked to indicate their primary ideological identification and the strength of their identification. Based on these responses, a 5-point variable was constructed ranging from 1 (“Very Liberal”) to 5 (“Very Conservative”). Answers -1, corresponding to “Unknown” (*n* = 29), were treated as missing data (*M* = 3.03, *SD* = 1.15).

#### Party identification

Respondents were asked to indicate their primary party identification and the strength of their identification. Other respondents were asked if they “Lean Democrat,” “Lean Republican,” or “Don’t lean.” Based on these responses, a 7-point variable was constructed ranging from 1 (“Strong Democrat”) to 7 (“Strong Republican”). Answers −1, corresponding to “Unknown” (*n* = 2), were treated as missing data (*M* = 3.77, *SD* = 2.14).

#### Ideological extremism

We created a variable based on the absolute value of distance from the ideological center. Thus, participants who chose 1 (extremely liberal) or 5 (extremely conservative) received an extremism score of 2, whereas participants who chose the scale midpoint received a score of 0 (*Mdn* = 1, *SD* = 0.82).

#### Partisan extremism

We created a variable based on the absolute value of distance from the party identification center. Thus, participants who chose 1 (extremely Democratic) or 7 (extremely Republican) received an extremism score of 3, whereas participants who chose the scale midpoint received a score of 0 (*Mdn* = 2, *SD* = 1.07).

#### RWA

Three items were administered to measure individual differences in RWA (e.g., “What our country really needs instead of more ‘civil rights’ is a good stiff dose of law and order”). Respondents indicated their levels of (dis)agreement on a scale from 1 (“Strongly disagree”) to 7 (“Strongly agree”). The measurement demonstrated good reliability (Cronbach’s alpha = 0.659). We created a composite variable based on factor scores; higher scores indicate higher levels of RWA (*Mdn* = 0.007, *SD* = 0.950).

#### SDO

Four items were administered to measure individual differences in SDO (e.g. “Some groups of people are simply inferior to others”). Respondents indicated their levels of (dis)agreement on a scale from 1 (“Strongly disagree”) to 7 (“Strongly agree”). The measurement demonstrated good reliability (Cronbach’s alpha = 0.681). We created a composite variable based on factor scores; higher scores indicate higher levels of SDO (*Mdn* = 0.027, *SD* = 0.826).

#### PSJ

Four items were administered to measure individual differences in PSJ (e.g. “The American political system is the best system there is”). Respondents indicated their levels of (dis)agreement on a scale from 1 (“Strongly disagree”) to 7 (“Strongly agree”). The measurement demonstrated good reliability (Cronbach’s alpha = 0.679). We created a composite variable based on factor scores; higher scores indicate higher levels of PSJ (*Mdn* = −0.049, *SD* = 0.848).

#### Support for democratic principles

Respondents indicated their levels of (dis)agreement on a scale from 1 (“Strongly disagree”) to 7 (“Strongly agree”) for 13 items pertaining to different democratic principles (e.g. “Everyone should be allowed to vote”; “Everyone should be allowed to express any idea, even potentially dangerous ideas”; “In order for a leader’s actions to be legitimate, they need to follow the rules”).

The measurement demonstrated good reliability (Cronbach’s alpha = 0.790). For the factor analysis, we used 12 items, excluding the item “Two adults who love each other should be allowed to get married, regardless of sexual orientation,” because we expected that it would be strongly correlated with ideological self-placement for reasons that may or may not pertain to anti-democratic sentiment. Factor analysis for the 12 items resulted in a three-factor solution. We created composite variables based on factor scores. The first factor was comprised of items about legal rights and guarantees according to the democratic rule of law (“Legal Rights and Guarantees”: *Mdn* = 0.275, *SD* = 0.926); the second factor was comprised of items about freedom of speech (“Freedom of Speech”: *Mdn* = −0.042, *SD* = 0.856); the third factor was comprised of items about political equality in voting (“Political Equality”: *Mdn* = 0.169, *SD* = 0.858). Higher scores indicate more support for democratic principles.

#### Defection from democratic rules of the game

Respondents indicated their levels of (dis)agreement on a scale from 1 (“Strongly disagree”) to 7 (“Strongly agree”) for three items measuring defection from democratic norms taken from McClosky’s^57^ work (e.g. “I don’t mind a politician’s methods if they manage to get the right things done”). The measurement demonstrated good reliability (Cronbach’s alpha = 0.609). We created a composite variable based on factor scores; higher scores indicate more willingness to defect from the democratic rules of the game (*Mdn* = 0.033, *SD* = 0.794).

#### Willingness to vote for blatantly anti-democratic candidate

Respondents were asked to imagine a candidate for the U.S. Senate from their own state whom they would like to support. They were then asked to use a 7-point scale ranging from 1 (“Much less likely to vote for them”) to 7 (“Much more likely to vote for them”) to indicate how three hypothetical anti-democratic statements (e.g. “I don’t care if the courts say this election is legitimate. I will decide whether to accept the results or not”) would affect their vote. The measurement demonstrated good reliability (Cronbach’s alpha = 0.759). We created a composite variable based on factor scores; higher scores indicate greater willingness to support anti-democratic candidates (*Mdn* = −0.211, *SD* = 0.918).

#### Tolerance of least liked groups

Respondents were asked to rank five groups (Black Lives Matter supporters, Make America Great Again supporters, Atheists, Racists, and Muslim Extremists) from most to least favored. Frequencies of least liked groups by Democratic and Republican respondents are presented in Supplementary Note 1 (Supplementary Table 2).

The group identified as least favored was used in three questions about political tolerance, in which respondents indicated their levels of (dis)agreement on a scale from 1 (“Strongly disagree”) to 7 (“Strongly agree”) for three statements such as “A book written by a member of [Least liked group] should be allowed in our local library.” The measurement demonstrated good reliability (Cronbach’s alpha = 0.864). We created a composite variable based on factor scores; higher scores indicate greater political tolerance (*Mdn* = 0.001, *SD* = 0.940).

#### Willingness to use political violence

Respondents were asked to indicate their levels of (dis)agreement on a scale from 1 (“Strongly disagree”) to 7 (“Strongly agree”) with four items regarding beliefs about whether American politics are in a state of crisis and support for political violence (e.g. “The United States is on the brink of a new civil war”; “I would personally be willing to use violence to ensure that a [if Democrat, show Democratic; if Republican, show Republican; if Independent, show Democratic] party candidate wins the 2024 presidential election”). A fifth item (“Joe Biden and the Democrats stole the 2020 presidential election”) was also administered, but we excluded it from analysis because it contained explicit political content that would obviously be more agreeable to Republicans than Democrats. Indeed, Republicans were far more likely than Democrats to “Agree strongly” with this statement (Republicans = 20.2%, Democrats = 0.69%; *Z* = 8.21, *p* < 0.001, *r* .683).

The measurement demonstrated good reliability (Cronbach’s alpha = 0.609); factor analysis resulted in a two-factor solution. The first factor captured willingness to use political violence; the second factor captured beliefs about an American political crisis. We created composite variables based on factor scores, but only the first factor (“Willingness to Use Political Violence”) was used, because (as noted by an anonymous reviewer) the second factor was not unambiguously related to anti-democratic tendencies. Higher scores indicate more willingness to use political violence (*Mdn* = −0.492, *SD* = 0.939).

#### Attitudes toward the January 6 insurrectionists

Respondents were asked to rate their feelings about eight political entities from 0 (“Cold”) to 100 (“Warm”), including the January 6 insurrectionists. We rescaled the variable to range from 0 to 1, then created a binary variable for warmer feelings towards January 6 insurrection by dummy-coding as follows: 1 = above 0.5; 0 = below 0.5. Additional analyses of affective polarization based on feeling thermometer variables are broken down by party affiliation in Supplementary Discussion 1 (Supplementary Fig. 1 and Supplementary Table 52).

#### Demographic variables

In all regression analyses, we adjusted for respondents’ demographic characteristics. We accounted for race and ethnicity with three dummy-coded variables for White (*N* = 1025, *Mode* = 1), Black (*N* = 193, *Mode* = 0), and Latino (*N* = 233, *Mode* = 0). Respondents were asked to type in their age in years (*Mdn* = 52, *SD* = 16.97), and to indicate their sex (1 = male respondents, 2 = female respondents; *Mode* = 2), income (nine levels ranging from 1 = Under $10,000 to 9 = $150,000 or more; *Mdn* = 6, *SD* = 2.29), and education (five levels ranging from 1 = Less than High School to 5 = Postgraduate study or Professional degree; *Mdn* = 3, *SD* = 1.07).

### Reporting summary

Further information on research design is available in the Nature Portfolio Reporting Summary linked to this article.

## Results

### Bivariate correlations

Consistent with previous research, conservatism was positively and significantly correlated with RWA, SDO, and PSJ. Identification with the Republican Party was also positively and significantly correlated with RWA, SDO, and PSJ. These effect sizes, some of which are quite high by the standards of behavioral science, are shown in Table 1.

**Table 1:** Bivariate correlations between political and psychological variables

	RWA	SDO	PSJ
Political conservatism (Ideological self-placement, 1–5)	0.491 [0.45, 0.53] *p* < 0.001 *N* = 1507	0.294 [0.25, 0.34] *p* < 0.001 *N* = 1507	0.169 [0.12, 0.22] *p* < 0.001 *N* = 1513
Strength of Republican (vs. Democratic) Party identification (1–7)	0.493 [0.45, 0.53] *p* < 0.001 *N* = 1530	0.256 [0.21, 0.30] *p* < 0.001 *N* = 1530	0.108 [0.06, 0.16] *p* < 0.001 *N* = 1540

Bivariate two-tailed Spearman correlations. Brackets contain 95% confidence intervals.

*RWA* Right-wing authoritarianism, *SDO* Social dominance orientation, *PSJ* Political system justification.

Importantly, conservatism and identification with the Republican Party were negatively correlated with support for legal rights and guarantees and political equality; and positively correlated with willingness to defect from the rules of the game, use political violence, and vote for blatantly anti-democratic candidates. These findings are consistent with the asymmetrical notion that liberals are more committed than conservatives to democratic norms and practices^39^. Conservatism and identification with the Republican Party were positively correlated with support for freedom of speech, and there was no statistically significant effect with political tolerance. These results are shown in Table 2.

**Table 2:** Bivariate correlations for pro- and anti-democratic attitudes, ideology, party identification, RWA, SDO, and PSJ

	1	2	3	4	5	6	7	8	9	10	11	12
1. Legal Rights and Guarantees	1 *N* = 1499											
2. Political Equality	0.644 [0.61, 0.67] *p* < 0.001 *N* = 1499	1 *N* = 1499										
3. Freedom of Speech	0.071 [0.02, 0.12] *p* = 0.006 *N* = 1499	−0.054 [−0.10, −0.00] *p* = 0.035 *N* = 1499	1 *N* = 1499									
4. Defection from Rules of the Game	−0.269 [−0.31, −0.22] *p* < 0.001 *N* = 1492	−0.233 [−0.28, −0.18] *p* < 0.001 *N* = 1492	0.180 [0.13, 0.23] *p* < 0.001 *N* = 1492	1 *N* = 1544								
5. Willingness to Use Violence	−0.328 [−0.37, −0.28] *p* < 0.001 *N* = 1477	−0.239 [−0.29, −0.20] *p* < 0.001 *N* = 1477	0.041 [−0.01, 0.09] *p* = 0.117 *N* = 1477	0.368 [0.32, 0.41] *p* < 0.001 *N* = 1519	1 *N* = 1529							
6. Tolerance of Disliked Group	0.042 [−0.01, 0.09] *p* = 0.119 *N* = 1373	0.040 [−0.01, 0.09] *p* = 0.136 *N* = 1373	0.257 [0.21, 0.31] *p* < 0.001 *N* = 1373	−0.153 [−0.20, −0.10] *p* < 0.001 *N* = 1410	0.000 [−0.05, 0.05] *p* = 0.995 *N* = 1400	1 *N* = 1416						
7. Willingness to Vote for Anti-democratic Candidate	−0.389 [−0.43, −0.34] *p* < 0.001 *N* = 1463	−0.447 [−0.49, −0.40] *p* < 0.001 *N* = 1463	0.198 [0.25, 0.15] *p* < 0.001 *N* = 1463	0.409 [0.37, 0.45] *p* < 0.001 *N* = 1503	0.306 [0.26, 0.35] *p* < 0.001 *N* = 1490	−0.041 [−0.09, 0.01] *p* = 0.129 *N* = 1383	1 *N* = 1512					
8. Political Conservatism	−0.235 [−0.28, −0.19] *p* < 0.001 *N* = 1472	−0.479 [−0.52, −0.44] *p* < 0.001 *N* = 1472	0.168 [0.12, 0.22] *p* < 0.001 *N* = 1472	0.238 [0.19, 0.28] *p* < 0.001 *N* = 1516	0.080 [0.03, 0.12] *p* = 0.002 *N* = 1506	−0.025 [−0.08, 0.03] *p* = 0.343 *N* = 1402	0.392 [0.35, 0.43] *p* < 0.001 *N* = 1489	1 *N* = 1528				
9. Strength of Republican (vs. Democratic) Party ID	−0.202 [−0.25, −0.15] *p* < 0.001 *N* = 1498	−0.495 [−0.53, −0.46] *p* < 0.001 *N* = 1498	0.208 [0.16, 0.26] *p* < 0.001 *N* = 1498	0.238 [0.19, 0.28] *p* < 0.001 *N* = 1542	0.067 [0.02, 0.12] *p* = 0.009 *N* = 1527	−0.040 [−0.09, 0.01] *p* = 0.136 *N* = 1414	0.428 [0.39, 0.47] *p* < 0.001 *N* = 1510	0.651 [0.62, 0.68] *p* < 0.001 *N* = 1528	1 *N* = 1555			
10. RWA	−0.205 [−0.25, −0.16] *p* < 0.001 *N* = 1481	−0.434 [−0.47, −0.39] *p* < 0.001 *N* = 1481	0.163 [0.11, 0.21] *p* < 0.001 *N* = 1481	0.476 [0.44, 0.51] *p* < 0.001 *N* = 1522	0.197 [0.15, 0.24] *p* < 0.001 *N* = 1514	−0.157 [−0.21, −0.10] *p* < 0.001 *N* = 1406	0.407 [0.36, 0.45] *p* < 0.001 *N* = 1493	0.491 [0.45, 0.53] *p* < 0.001 *N* = 1507	0.493 [0.45, 0.53] *p* < 0.001 *N* = 1530	1 *N* = 1532		
11. SDO	−0.488 [−0.53, −0.45] *p* < 0.001 *N* = 1481	−0.433 [−0.47, −0.39] *p* < 0.001 *N* = 1481	0.080 [0.03, 0.13] *p* = 0.002 *N* = 1481	0.399 [0.35, 0.44] *p* < 0.001 *N* = 1522	0.346 [0.30, 0.39] *p* < 0.001 *N* = 1509	−0.018 [−0.07, 0.03] *p* = 0.507 *N* = 1400	0.431 [0.39, 0.47] *p* < 0.001 *N* = 1493	0.294 [0.25, 0.34] *p* < 0.001 *N* = 1507	0.256 [0.21, 0.30] *p* < 0.001 *N* = 1530	0.339 [0.29, 0.38] *p* < 0.001 *N* = 1513	1 *N* = 1532	
12. PSJ	0.054 [0.00, 0.10] *p* = 0.038 *N* = 1489	−0.094 [−0.14, −0.04] *p* < 0.001 *N* = 1489	0.151 [0.10, 0.20] *p* < 0.001 *N* = 1489	−0.144 [−0.19, −0.09] *p* < 0.001 *N* = 1531	−0.130 [−0.18, −0.08] *p* < 0.001 *N* = 1519	0.151 [0.10, 0.20] *p* < 0.001 *N* = 1406	−0.028 [−0.08, 0.02] *p* = 0.274 *N* = 1500	0.169 [0.12, 0.22] *p* < 0.001 *N* = 1513	0.108 [0.06, 0.16] *p* < 0.001 *N* = 1540	0.023 [−0.03, 0.07] *p* = 0.374 *N* = 1522	0.040 [−0.01, 09] *p* = 0.116 *N* = 1522	1 *N* = 1542

Bivariate two-tailed Spearman correlations. Brackets contain 95% confidence intervals.

*RWA* Right-wing authoritarianism, *SDO* Social dominance orientation, *PSJ* Political system justification.

### Multiple regression analyses examining the effects of political and psychological variables on pro- and anti-democratic tendencies

To investigate the simultaneous effects of political and psychological variables on anti-democratic tendencies, we performed hierarchical ordinary least squares regressions for each of the seven dependent variables. Table 3 summarizes regression results for the first four dependent variables (for complete regression results, see Supplementary Note 2: Supplementary Tables 3–26).

**Table 3:** Results of hierarchical regression models for attitudes toward various democratic principles and willingness to defect from democratic “rules of the game”

Independent variables	Legal rights and guarantees			Political equality			Freedom of speech			Defection from rules of the game		
	β (SE)	*p*	Std. 95% CI	β (SE)	*p*	Std. 95% CI	β (SE)	*p*	Std. 95% CI	β (SE)	*p*	Std. 95% CI
**Step 1: demographic variables**												
White	0.06 (0.10)	0.258	[−0.04, 0.16]	−0.02 (0.08)	0.644	[−0.11, 0.07]	0.02 (0.09)	0.660	[−0.08, 0.12]	−0.09 (0.08)	0.056	[−0.19, 0.00]
Black	−0.02 (0.12)	0.592	[−0.11, 0.06]	0.11 (0.10)	0.003	[0.04, 0.18]	−0.05 (0.11)	0.213	[−0.13, 0.03]	0.02 (0.10)	0.702	[−0.06, 0.09]
Latino	−0.09 (0.12)	0.068	[−0.18, 0.01]	−0.04 (0.10)	0.283	[−0.12, 0.04]	0.07 (0.10)	0.133	[−0.02, 0.15]	0.04 (0.09)	0.338	[−0.04, 0.12]
Age	0.20 (0.00)	<0.001	[0.15, 0.25]	0.02 (0.00)	0.417	[−0.03, 0.07]	−0.02 (0.00)	0.370	[−0.07, 0.03]	−0.05 (0.00)	0.031	[−0.10, −0.01]
Sex (1 = male, 2 = female)	0.01 (0.05)	0.556	[−0.03, 0.06]	0.06 (0.04)	0.023	[0.01, 0.11]	−0.13 (0.04)	<0.001	[−0.18, −0.08]	0.01 (0.04)	0.593	[−0.03, 0.06]
Income	0.09 (0.01)	0.002	[0.03, 0.15]	−0.01 (0.01)	0.818	[−0.06, 0.05]	−0.02 (0.01)	0.393	[−0.08, 0.03]	−0.03 (0.01)	0.225	[−0.09, 0.02]
Education	0.15 (0.02)	<0.001	[0.10, 0.20]	0.15 (0.02)	<0.001	[0.10, 0.21]	−0.01 (0.02)	0.782	[−0.06, 0.05]	−0.26 (0.02)	<0.001	[−0.31, −0.21]
*N*	1,490			1,490			1,490			1,534		
R^2^_adj_	0.12			0.04			0.02			0.11		
**Step 2: ideological variables**												
Conservatism	−0.16 (0.03)	<0.001	[−0.23, −0.09]	−0.27 (0.02)	<0.001	[−0.33, −0.21]	0.07 (0.03)	0.056	[−0.00, 0.13]	0.14 (0.02)	<0.001	[0.07, 0.21]
Republican ID	−0.01 (0.01)	0.749	[−0.08, 0.06]	−0.29 (0.01)	<0.001	[−0.35, −0.23]	0.17 (0.01)	<0.001	[0.11, 0.24]	0.14 (0.01)	<0.001	[0.08, 0.21]
*N*	1472			1472			1472			1516		
R^2^_adj_	0.03			0.26			0.05			0.07		
**Step 3: demographic and ideological variables**												
White	0.07 (0.10)	0.176	[−0.03, 0.17]	0.05 (0.08)	0.248	[−0.03, 0.13]	−0.01 (0.09)	0.841	[−0.11, 0.09]	−0.13 (0.08)	0.005	[−0.23, −0.04]
Black	−0.02 (0.11)	0.677	[−0.10, 0.06]	0.07 (0.09)	0.033	[0.01, 0.14]	−0.02 (0.11)	0.718	[−0.10, 0.07]	0.06 (0.10)	0.135	[−0.02, 0.14]
Latino	−0.10 (0.12)	0.042	[−0.19, −0.00]	−0.05 (0.09)	0.227	[−0.12, 0.03]	0.07 (0.10)	0.119	[−0.02, 0.15]	0.03 (0.09)	0.423	[−0.05, 0.11]
Age	0.22 (0.00)	<0.001	[0.17, 0.27]	0.07 (0.00)	0.003	[0.02, 0.11]	−0.05 (0.00)	0.045	[−0.10, 0.00]	−0.09 (0.00)	<0.001	[−0.14, −0.04]
Sex (1 = male, 2 = female)	0.01 (0.04)	0.726	[−0.04, 0.06]	0.03 (0.04)	0.139	[−0.01, 0.08]	−0.12 (0.04)	<0.001	[−0.17, −0.07]	0.03 (0.04)	0.179	[−0.01, 0.08]
Income	0.09 (0.01)	0.002	[0.04, 0.15]	−0.00 (0.01)	0.878	[−0.05, 0.04]	−0.03 (0.01)	0.232	[−0.09, 0.02]	−0.03 (0.01)	0.178	[−0.09, 0.02]
Education	0.10 (0.02)	<0.001	[0.05, 0.15]	0.05 (0.02)	0.026	[0.01, 0.10]	0.03 (0.02)	0.279	[−0.02, 0.09]	−0.21 (0.02)	<0.001	[−0.26, −0.16]
Conservatism	−0.16 (0.03)	<0.001	[−0.23, −0.10]	−0.27 (0.02)	<0.001	[−0.33, −0.21]	0.07 (0.03)	0.040	[0.00, 0.14]	0.11 (0.02)	0.001	[0.05, 0.18]
Republican ID	−0.05 (0.01)	0.178	[−0.12, 0.02]	−0.28 (0.01)	<0.001	[−0.35, −0.22]	0.17 (0.01)	<0.001	[0.11, 0.24]	0.20 (0.01)	<0.001	[0.13, 0.27]
*N*	1463			1463			1463			1506		
R^2^_adj_	0.15			0.27			0.07			0.18		
**Step 4: demographic, ideological, and extremism variables**												
White	0.05 (0.09)	0.322	[−0.05, 0.15]	0.04 (0.08)	0.362	[−0.04, 0.12]	−0.01 (0.09)	0.824	[−0.11, 0.09]	−0.14 (0.08)	0.005	[−0.23, −0.04]
Black	−0.03 (0.11)	0.467	[−0.11, 0.05]	0.06 (0.09)	0.062	[−0.00, 0.13]	−0.01 (0.11)	0.778	[−0.09, 0.07]	0.05 (0.10)	0.171	[−0.02, 0.13]
Latino	−0.11 (0.12)	0.023	[−0.20, −0.02]	−0.05 (0.09)	0.174	[−0.13, 0.02]	0.07 (0.10)	0.121	[−0.02, 0.15]	0.03 (0.09)	0.446	[−0.05, 0.11]
Age	0.21 (0.00)	<0.001	[0.16, 0.26]	0.06 (0.00)	0.014	[0.01, 0.10]	−0.05 (0.00)	0.072	[−0.10, 0.00]	−0.09 (0.00)	<0.001	[−0.14, −0.05]
Sex (1 = male, 2 = female)	0.00 (0.04)	0.861	[−0.04, 0.05]	0.03 (0.04)	0.200	[−0.02, 0.07]	−0.11 (0.04)	<0.001	[−0.16, −0.06]	0.03 (0.04)	0.257	[−0.02, 0.07]
Income	0.08 (0.01)	0.003	[0.03, 0.14]	−0.01 (0.01)	0.662	[−0.06, 0.04]	−0.03 (0.01)	0.326	[−0.08, 0.03]	−0.04 (0.01)	0.112	[−0.09, 0.01]
Education	0.10 (0.02)	<0.001	[0.05, 0.15]	0.05 (0.02)	0.029	[0.01, 0.10]	0.03 (0.02)	0.319	[−0.03, 0.08]	−0.21 (0.02)	<0.001	[−0.26, −0.16]
Conservatism	−0.16 (0.03)	<0.001	[−0.23, −0.10]	−0.28 (0.02)	<0.001	[−0.34, −0.21]	0.08 (0.03)	0.027	[0.01, 0.15]	0.11 (0.02)	0.002	[0.04, 0.18]
Republican ID	−0.04 (0.01)	0.235	[−0.11, 0.03]	−0.28 (0.01)	<0.001	[−0.34, −0.21]	0.16 (0.01)	<0.001	[0.09, 0.23]	0.21 (0.01)	<0.001	[0.14, 0.28]
Ideological extremism	0.05 (0.03)	0.069	[−0.00, 0.10]	−0.00 (0.03)	0.976	[−0.05, 0.05]	0.06 (0.03)	0.054	[−0.00, 0.11]	−0.05 (0.03)	0.070	[−0.10, 0.00]
Partisan extremism	0.08 (0.03)	0.008	[0.02, 0.14]	0.06 (0.02)	0.020	[0.01, 0.12]	−0.04 (0.02)	0.217	[−0.09, 0.02]	0.05 (0.02)	0.093	[−0.01, 0.10]
*N*	1463			1463			1463			1506		
R^2^_adj_	0.16			0.28			0.07			0.18		
**Step 5: demographic, ideological, and psychological variables**												
White	0.04 (0.09)	0.382	[−0.05, 0.13]	−0.00 (0.07)	0.973	[−0.07, 0.07]	−0.00 (0.10)	0.995	[−0.10, 0.10]	−0.11 (0.08)	0.012	[−0.20, −0.03]
Black	−0.02 (0.11)	0.590	[−0.10, 0.06]	0.05 (0.08)	0.113	[−0.01, 0.11]	−0.01 (0.11)	0.735	[−0.10, 0.07]	0.03 (0.09)	0.432	[−0.04, 0.10]
Latino	−0.10 (0.11)	0.029	[−0.18, −0.01]	−0.07 (0.08)	0.058	[−0.13, 0.00]	0.06 (0.11)	0.151	[−0.02, 0.15]	0.02 (0.09)	0.579	[−0.05, 0.10]
Age	0.18 (0.00)	<0.001	[0.14, 0.23]	0.06 (0.00)	0.005	[0.02, 0.11]	−0.09 (0.00)	0.001	[−0.14, −0.04]	−0.06 (0.00)	0.007	[−0.11, −0.02]
Sex (1 = male, 2 = female)	0.01 (0.04)	0.733	[−0.04, 0.06]	0.03 (0.04)	0.121	[−0.01, 0.08]	−0.11 (0.04)	<0.001	[−0.16, −0.06]	0.01 (0.03)	0.746	[−0.04, 0.05]
Income	0.07 (0.01)	0.009	[0.02, 0.13]	−0.01 (0.01)	0.533	[−0.06, 0.03]	−0.04 (0.01)	0.132	[−0.10, 0.01]	−0.00 (0.01)	0.882	[−0.05, 0.04]
Education	0.08 (0.02)	0.001	[0.03, 0.13]	0.00 (0.02)	0.861	[−0.04, 0.05]	0.04 (0.02)	0.168	[−0.02, 0.10]	−0.11 (0.02)	<0.001	[−0.15, −0.06]
Conservatism	−0.09 (0.02)	0.007	[−0.15, −0.02]	−0.17 (0.02)	<0.001	[−0.23, −0.11]	0.04 (0.03)	0.328	[−0.04, 0.11]	0.00 (0.02)	0.989	[−0.06, 0.06]
Republican ID	−0.02 (0.01)	0.581	[−0.09, 0.05]	−0.21 (0.01)	<0.001	[−0.27, −0.15]	0.14 (0.01)	<0.001	[0.07, 0.22]	0.05 (0.01)	0.115	[−0.01, 0.11]
RWA	0.05 (0.03)	0.101	[−0.01, 0.11]	−0.15 (0.02)	<0.001	[−0.20, −0.09]	0.08 (0.03)	0.025	[0.01, 0.15]	0.37 (0.02)	<0.001	[0.31, 0.43]
SDO	−0.37 (0.03)	<0.001	[−0.43, −0.31]	−0.26 (0.03)	<0.001	[−0.31, −0.21]	−0.00 (0.03)	0.949	[−0.06, 0.06]	0.22 (0.02)	<0.001	[0.17, 0.27]
PSJ	0.01 (0.02)	0.694	[−0.04, 0.06]	−0.04 (0.02)	0.120	[−0.08, 0.01]	0.14 (0.03)	<0.001	[0.08, 0.20]	−0.12 (0.02)	<0.001	[−0.16, −0.07]
*N*	1426			1426			1426			1462		
R^2^_adj_	0.26			0.36			0.09			0.36		
**Step 6: demographic, ideological, and psychological variables**												
White	0.03 (0.09)	0.543	[−0.06, 0.12]	−0.01 (0.07)	0.829	[−0.08, 0.07]	0.00 (0.10)	0.986	[−0.10, 0.11]	−0.12 (0.08)	0.009	[−0.21, −0.03]
Black	−0.03 (0.11)	0.430	[−0.11, 0.05]	0.04 (0.08)	0.191	[−0.02, 0.10]	−0.01 (0.12)	0.863	[−0.09, 0.08]	0.02 (0.09)	0.548	[−0.05, 0.10]
Latino	−0.10 (0.11)	0.018	[−0.19, −0.02]	−0.07 (0.08)	0.046	[−0.14, −0.00]	0.06 (0.11)	0.147	[−0.02, 0.15]	0.02 (0.09)	0.632	[−0.06, 0.09]
Age	0.17 (0.00)	<0.001	[0.12, 0.22]	0.06 (0.00)	0.015	[0.01, 0.10]	−0.09 (0.00)	0.001	[−0.14, −0.03]	−0.07 (0.00)	0.004	[−0.12, −0.02]
Sex (1 = male, 2 = female)	0.00 (0.04)	0.901	[−0.04, 0.05]	0.03 (0.04)	0.213	[−0.02, 0.07]	−0.10 (0.04)	<0.001	[−0.15, −0.05]	−0.00 (0.03)	0.923	[−0.04, 0.04]
Income	0.07 (0.01)	0.014	[0.01, 0.12]	−0.02 (0.01)	0.354	[−0.07, 0.02]	−0.03 (0.01)	0.233	[−0.09, 0.02]	−0.01 (0.01)	0.700	[−0.05, 0.04]
Education	0.08 (0.02)	0.001	[0.03, 0.13]	0.00 (0.02)	0.844	[−0.04, 0.05]	0.04 (0.02)	0.195	[−0.02, 0.09]	−0.11 (0.02)	<0.001	[−0.15, −0.06]
Conservatism	−0.08 (0.02)	0.008	[−0.15, −0.02]	−0.17 (0.02)	<0.001	[−0.23, −0.11]	0.04 (0.03)	0.269	[−0.03, 0.11]	−0.00 (0.02)	0.979	[−0.06, 0.06]
Republican ID	−0.01 (0.01)	0.701	[−0.08, 0.06]	−0.20 (0.01)	<0.001	[−0.26, −0.13]	0.13 (0.01)	0.001	[0.05, 0.20]	0.06 (0.01)	0.074	[−0.01, 0.12]
Ideological extremism	0.02 (0.03)	0.352	[−0.03, 0.07]	−0.03 (0.02)	0.236	[−0.08, 0.02]	0.07 (0.03)	0.012	[0.02, 0.13]	−0.02 (0.02)	0.506	[−0.06, 0.03]
Partisan extremism	0.07 (0.02)	0.018	[0.01, 0.13]	0.07 (0.02)	0.014	[0.01, 0.12]	−0.06 (0.02)	0.052	[−0.12, 0.00]	0.05 (0.02)	0.056	[−0.00, 0.10]
RWA	0.05 (0.03)	0.125	[−0.01, 0.11]	−0.15 (0.02)	<0.001	[−0.20, −0.10]	0.08 (0.03)	0.016	[0.02, 0.15]	0.37 (0.02)	<0.001	[0.31, 0.43]
SDO	−0.36 (0.03)	<0.001	[−0.42, −0.31]	−0.26 (0.03)	<0.001	[−0.31, −0.21]	−0.00 (0.03)	0.991	[−0.06, 0.06]	0.22 (0.02)	<0.001	[0.17, 0.27]
PSJ	0.00 (0.02)	0.904	[−0.04, 0.05]	−0.04 (0.02)	0.062	[−0.09, 0.00]	0.15 (0.03)	<0.001	[0.09, 0.21]	−0.12 (0.02)	<0.001	[−0.17, −0.07]
*N*	1426			1426			1426			1462		
R^2^_adj_	0.26			0.36			.09			0.36		

*RWA* Right-wing authoritarianism, *SDO* Social dominance orientation, *PSJ* Political system justification.

Brackets contain standardized 95% confidence intervals.

As shown in Step 1, some demographic variables were significant predictors of democratic vs. anti-democratic sentiment. Black respondents were more committed to political equality. Older respondents were more supportive of legal rights and guarantees and less likely to defect from the rules of the game. Female respondents were more committed to political equality, whereas male respondents were more committed to free speech. Wealthier respondents were more supportive of legal rights and guarantees. Highly educated respondents were more supportive of rights and guarantees and political equality and less likely to defect from the rules of the game.

In Step 2, liberals were more supportive of legal rights and guarantees and political equality and less likely to defect from the rules of the game. People identified with the Republican Party were less committed to political equality and more likely to defect from the rules of the game, but they were also more supportive of free speech. These results remain significant after adjusting for demographic variables (Step 3) and extremism (Step 4). In Step 4, partisan extremists were more supportive of legal rights and guarantees and political equality compared to moderates.

In Step 5, we estimated the effects of political and psychological variables simultaneously while adjusting for demographic variables. Liberals remained more supportive of legal rights and guarantees and political equality, but these effects were somewhat weaker in Step 5 than in Steps 2, 3, and 4. Those identified with the Republican Party remained less committed to political equality and more supportive of free speech. Conservatives and those identified with the Republican Party were more likely to defect from the rules of the game in Steps 2, 3, and 4 but not Step 5, suggesting that these differences may be attributable in part to demographic and/or psychological differences.

In Step 5, respondents who scored higher on RWA were less committed to political equality and more likely to defect from the rules of the game but, unexpectedly, more supportive of free speech. Respondents who scored higher on SDO were less supportive of legal rights and guarantees and political equality and more likely to defect from the rules of the game. Respondents who scored higher on PSJ were more supportive of free speech and less likely to defect from the rules of the game.

In Step 6, we estimated the effects of political and psychological variables while adjusting for demographic variables and extremism. As in Step 5, liberals remained more supportive than conservatives of legal rights and guarantees and political equality. Those identified with the Republican Party remained less committed to political equality and more supportive of free speech. Ideological extremists were more supportive of free speech compared to moderates. Partisan extremists remained more supportive of legal rights and guarantees and political equality compared to moderates. Even after adjusting for all other variables, results for RWA, SDO, and PSJ found in Step 5 remained statistically significant in Step 6.

Table 4 summarizes results for the next three dependent variables (for complete regression results, see Supplementary Note 2: Supplementary Tables 27–44). As shown in Step 1, older respondents were less likely to support anti-democratic candidates or use violence against political opponents. Female respondents were more likely to tolerate their least liked group. Poorer respondents were more willing to use violence. Less educated respondents were more willing to advocate violence, less tolerant of disliked groups, and more supportive of anti-democratic candidates.

**Table 4:** Results of hierarchical regression models for willingness to use political violence, tolerance of disliked groups, and willingness to vote for anti-democratic candidates

Independent variables	Willingness to use violence			Tolerance of disliked group			Willingness to vote for anti-democratic candidate		
	β (SE)	*p*	Std. 95% CI	β (SE)	*p*	Std. 95% CI	β (SE)	*p*	Std. 95% CI
**Step 1: demographic variables**									
White	−0.05 (0.09)	0.334	[−0.14, 0.05]	0.08 (0.11)	0.154	[−0.03, 0.18]	0.06 (0.08)	0.198	[−0.03, 0.14]
Black	0.08 (0.13)	0.052	[−0.00, 0.17]	−0.02 (0.12)	0.565	[−0.11, 0.06]	0.03 (0.10)	0.458	[−0.05, 0.10]
Latino	0.05 (0.11)	0.214	[−0.03, 0.14]	0.02 (0.12)	0.631	[−0.07, 0.11]	0.07 (0.10)	0.064	[−0.00, 0.15]
Age	−0.16 (0.00)	<0.001	[−0.21, −0.11]	0.02 (0.00)	0.525	[−0.04, 0.07]	−0.07 (0.00)	0.005	[−0.12, −0.02]
Sex (1 = male, 2 = female)	0.00 (0.05)	0.972	[−0.05, 0.05]	−0.10 (0.05)	<0.001	[−0.16, −0.05]	0.02 (0.05)	0.479	[−0.03, 0.07]
Income	−0.09 (0.01)	0.002	[−0.15, −0.03]	0.00 (0.01)	0.953	[−0.06, 0.06]	−0.03 (0.01)	0.273	[−0.09, 0.02]
Education	−0.13 (0.02)	<0.001	[−0.18, −0.08]	0.08 (0.03)	0.006	[0.02, 0.14]	−0.25 (0.02)	<0.001	[−0.30, −0.19]
*N*	1519			1407			1502		
R^2^_adj_	0.09			0.02			0.08		
**Step 2: ideological variables**									
Conservatism	0.07 (0.03)	0.040	[0.00, 0.14]	−0.01 (0.03)	0.822	[−0.08, 0.06]	0.20 (0.02)	<0.001	[0.14, 0.26]
Republican ID	−0.02 (0.02)	0.655	[−0.09, 0.05]	−0.03 (0.01)	0.366	[−0.10, 0.04]	0.31 (0.01)	<0.001	[0.25, 0.37]
*N*	1506			1402			1489		
R^2^_adj_	0.00			0.00			0.22		
**Step 3: demographic and ideological variables**									
White	−0.06 (0.09)	0.232	[−0.15, 0.04]	0.10 (0.11)	0.066	[−0.01, 0.21]	−0.00 (0.08)	0.990	[−0.08, 0.08]
Black	0.09 (0.13)	0.044	[0.00, 0.18]	−0.03 (0.13)	0.552	[−0.11, 0.06]	0.10 (0.10)	0.005	[0.03, 0.18]
Latino	0.05 (0.11)	0.237	[−0.03, 0.14]	0.03 (0.13)	0.523	[−0.06, 0.12]	0.08 (0.10)	0.028	[0.01, 0.16]
Age	−0.17 (0.00)	<0.001	[−0.22, −0.12]	0.02 (0.00)	0.468	[−0.03, 0.07]	−0.12 (0.00)	<0.001	[−0.17, −0.08]
Sex (1 = male, 2 = female)	0.01 (0.05)	0.784	[−0.04, 0.06]	−0.11 (0.05)	<0.001	[−0.16, −0.06]	0.05 (0.04)	0.031	[0.00, 0.09]
Income	−0.09 (0.01)	0.003	[−0.15, −0.03]	0.01 (0.01)	0.866	[−0.05, 0.06]	−0.04 (0.01)	0.073	[−0.09, 0.00]
Education	−0.11 (0.02)	<0.001	[−0.16, −0.06]	0.07 (0.03)	0.019	[0.01, 0.13]	−0.15 (0.02)	<0.001	[−0.20, −0.10]
Conservatism	0.06 (0.03)	0.089	[−0.01, 0.13]	0.02 (0.03)	0.653	[−0.05, 0.09]	0.18 (0.02)	<0.001	[0.12, 0.24]
Republican ID	0.04 (0.02)	0.285	[−0.03, 0.11]	−0.07 (0.02)	0.041	[−0.14, −0.00]	0.35 (0.01)	<0.001	[0.29, 0.42]
*N*	1496			1393			1479		
R^2^_adj_	0.10			0.03			0.29		
**Step 4: demographic, ideological, and extremism variables**									
White	−0.06 (0.09)	0.243	[−0.15, 0.04]	0.10 (0.11)	0.062	[−0.01, 0.21]	−0.01 (0.08)	0.789	[−0.10, 0.07]
Black	0.09 (0.13)	0.042	[0.00, 0.18]	−0.02 (0.13)	0.651	[−0.11, 0.07]	0.10 (0.11)	0.008	[0.03, 0.17]
Latino	0.05 (0.11)	0.233	[−0.03, 0.14]	0.03 (0.13)	0.503	[−0.06, 0.12]	0.08 (0.10)	0.041	[0.01, 0.15]
Age	−0.17 (0.00)	<0.001	[−0.22, −0.12]	0.03 (0.00)	0.347	[−0.03, 0.08]	−0.13 (0.00)	<0.001	[−0.17, −0.09]
Sex (1 = male, 2 = female)	0.01 (0.05)	0.791	[−0.04, 0.06]	−0.11 (0.05)	<0.001	[−0.16, −0.05]	0.05 (0.04)	0.034	[0.00, 0.09]
Income	−0.09 (0.01)	0.001	[−0.15, −0.03]	0.01 (0.01)	0.685	[−0.05, 0.07]	−0.05 (0.01)	0.059	[−0.10, 0.00]
Education	−0.11 (0.02)	<0.001	[−0.16, −0.06]	0.07 (0.03)	0.022	[0.01, 0.13]	−0.15 (0.02)	<0.001	[−0.20, −0.11]
Conservatism	0.06 (0.03)	0.094	[−0.01, 0.13]	0.02 (0.03)	0.541	[−0.05, 0.09]	0.19 (0.02)	<0.001	[0.13, 0.25]
Republican ID	0.04 (0.02)	0.280	[−0.03, 0.11]	−0.08 (0.02)	0.018	[−0.15, −0.01]	0.35 (0.01)	<0.001	[0.29, 0.42]
Ideological extremism	−0.01 (0.03)	0.737	[−0.07, 0.05]	0.05 (0.03)	0.125	[−0.01, 0.10]	0.06 (0.03)	0.021	[0.01, 0.10]
Partisan extremism	−0.00 (0.03)	0.958	[−0.06, 0.06]	−0.05 (0.03)	0.082	[−0.11, 0.01]	0.03 (0.02)	0.222	[−0.02, 0.08]
*N*	1496			1393			1479		
R^2^_adj_	0.09			0.03			0.30		
**Step 5: demographic, ideological, and psychological variables**									
White	−0.04 (0.09)	0.414	[−0.13, 0.05]	0.10 (0.11)	0.066	[−0.01, 0.21]	0.01 (0.08)	0.729	[−0.07, 0.10]
Black	0.07 (0.12)	0.086	[−0.01, 0.16]	−0.03 (0.13)	0.447	[−0.12, 0.05]	0.10 (0.10)	0.008	[0.02, 0.17]
Latino	0.05 (0.11)	0.218	[−0.03, 0.13]	0.04 (0.13)	0.411	[−0.05, 0.13]	0.07 (0.10)	0.047	[0.00, 0.15]
Age	−0.15 (0.00)	<0.001	[−0.20, −0.10]	0.01 (0.00)	0.825	[−0.05, 0.06]	−0.11 (0.00)	<0.001	[−0.15, −0.06]
Sex (1 = male, 2 = female)	0.00 (0.04)	0.882	[−0.04, 0.05]	−0.10 (0.05)	<0.001	[−0.15, −0.04]	0.04 (0.04)	0.096	[−0.01, 0.08]
Income	−0.08 (0.01)	0.007	[−0.13, −0.02]	−0.01 (0.01)	0.758	[−0.07, 0.05]	−0.03 (0.01)	0.163	[−0.08, 0.01]
Education	−0.07 (0.02)	0.006	[−0.13, −0.02]	0.03 (0.03)	0.320	[−0.03, 0.09]	−0.10 (0.02)	<0.001	[−0.14, −0.05]
Conservatism	−0.03 (0.03)	0.473	[−0.10, 0.05]	0.05 (0.03)	0.144	[−0.02, 0.13]	0.09 (0.03)	0.004	[0.03, 0.15]
Republican ID	−0.02 (0.02)	0.621	[−0.09, 0.05]	−0.03 (0.02)	0.386	[−0.11, 0.04]	0.27 (0.01)	<0.001	[0.21, 0.34]
RWA	0.03 (0.03)	0.292	[−0.03, 0.09]	−0.18 (0.04)	<0.001	[−0.25, −0.11]	0.15 (0.03)	<0.001	[0.09, 0.20]
SDO	0.32 (0.03)	<0.001	[0.26, 0.37]	0.04 (0.03)	0.238	[−0.02, 0.10]	0.24 (0.03)	<0.001	[0.19, 0.29]
PSJ	0.02 (0.02)	0.271	[−0.02, 0.07]	0.12 (0.03)	<0.001	[0.06, 0.18]	−0.02 (0.02)	0.475	[−0.06, 0.03]
*N*	1457			1363			1439		
R^2^_adj_	0.19			0.06			0.37		
**Step 6: demographic, ideological, extremism, and psychological variables**									
White	−0.04 (0.09)	0.401	[−0.13, 0.05]	0.10 (0.11)	0.056	[−0.00, 0.21]	0.00 (0.08)	0.951	[−0.08, 0.09]
Black	0.07 (0.12)	0.085	[−0.01, 0.16]	−0.03 (0.13)	0.574	[−0.11, 0.06]	0.09 (0.11)	0.014	[0.02, 0.16]
Latino	0.05 (0.11)	0.224	[−0.03, 0.13]	0.04 (0.13)	0.389	[−0.05, 0.13]	0.07 (0.10)	0.068	[−0.01, 0.14]
Age	−0.15 (0.00)	<0.001	[−0.20, −0.10]	0.01 (0.00)	0.683	[−0.05, 0.07]	−0.12 (0.00)	<0.001	[−0.16, −0.07]
Sex (1 = male, 2 = female)	0.00 (0.05)	0.857	[−0.04, 0.05]	−0.09 (0.05)	0.001	[−0.14, −0.04]	0.04 (0.04)	0.094	[−0.01, 0.08]
Income	−0.07 (0.01)	0.008	[−0.13, −0.02]	−0.00 (0.01)	0.958	[−0.06, 0.06]	−0.03 (0.01)	0.154	[−0.08, 0.01]
Education	−0.07 (0.02)	0.005	[−0.13, −0.02]	0.03 (0.03)	0.334	[−0.03, 0.09]	−0.10 (0.02)	<0.001	[−0.15, −0.05]
Conservatism	−0.03 (0.03)	0.490	[−0.10, 0.05]	0.06 (0.03)	0.121	[−0.02, 0.13]	0.10 (0.03)	0.002	[0.04, 0.16]
Republican ID	−0.02 (0.02)	0.577	[−0.09, 0.05]	−0.05 (0.02)	0.230	[−0.12, 0.03]	0.27 (0.01)	<0.001	[0.20, 0.33]
Ideological extremism	0.02 (0.03)	0.498	[−0.04, 0.07]	0.04 (0.03)	0.146	[−0.01, 0.10]	0.08 (0.03)	<0.001	[0.04, 0.13]
Partisan extremism	−0.00 (0.03)	0.910	[−0.06, 0.05]	−0.06 (0.03)	0.046	[−0.12, −0.00]	0.03 (0.02)	0.199	[−0.02, 0.08]
RWA	0.03 (0.03)	0.279	[−0.03, 0.09]	−0.18 (0.04)	<0.001	[−0.25, −0.11]	0.15 (0.03)	<0.001	[0.09, 0.20]
SDO	0.32 (0.03)	<0.001	[0.26, 0.37]	0.04 (0.04)	0.242	[−0.02, 0.10]	0.24 (0.03)	<0.001	[0.19, 0.29]
PSJ	0.03 (0.03)	0.263	[−0.02, 0.07]	0.13 (0.03)	<0.001	[0.07, 0.19]	−0.02 (0.02)	0.449	[−0.06, 0.03]
*N*	1457			1363			1439		
R^2^_adj_	0.18			0.06			0.37		

Brackets contain standardized 95% confidence intervals.

*RWA* Right-wing authoritarianism, *SDO* Social dominance orientation, *PSJ* Political system justification.

In Step 2, liberals were less willing to use political violence than conservatives. Conservatives and those identified with the Republican Party were more willing to vote for blatantly anti-democratic candidates. These last two effects remained significant even after adjusting for demographic variables (Step 3) and extremism (Step 4). People identified with the Republican Party were also less tolerant of disliked groups in Steps 3 and 4. In Step 4, ideological extremists were more likely to endorse anti-democratic candidates.

In Step 5, conservatives and those identified with the Republican Party remained more willing to vote for anti-democratic candidates, but these effects were somewhat weaker in Step 5 than in Steps 2, 3, and 4, suggesting that the differences may be partially attributable to demographic and/or psychological variables. Respondents who scored higher on RWA were less tolerant of disliked groups and more willing to vote for anti-democratic candidates. Respondents who scored higher on SDO were also more willing to vote for anti-democratic candidates and to endorse political violence. Respondents who scored higher on PSJ were more tolerant of disliked groups.

In Step 6, we estimated the effects of political and psychological variables while adjusting for demographic variables and extremism. Conservatives and those identified with the Republican Party were more likely to vote for anti-democratic candidates, after adjusting for all other variables. Ideological extremists were also more willing to vote for anti-democratic candidates. Partisan extremists were less likely to tolerate disliked groups. Even after adjusting for all other variables, results for RWA, SDO, and PSJ found in Step 5 remained statistically significant in Step 6.

### Mediational analyses

To investigate the hypotheses that ideological asymmetries in anti-democratic tendencies are attributable to psychological differences, we conducted a Structural Equation Model. Structural Equation Modeling is sometimes confused with causal analysis^58^, but it is important to keep in mind that causal assumptions—like other philosophical assumptions—are not tied to any particular statistical technique. To be perfectly clear, we made no causal assumptions about the developmental sequence of political attitude acquisition in adolescence or adulthood. Rather, we used Structural Equation Modeling as a way of illuminating *why*, psychologically speaking, liberals and conservatives might differ in terms of anti-democratic tendencies. In other words, we inquired about the extent to which conservatives’ relative lack of commitment to democratic norms and principles was attributable, at least in part, to individual differences in RWA, SDO, and PSJ.

We focused on the five outcomes for which ideological asymmetries emerged in the hierarchical regression models, namely support for legal rights and guarantees, political equality, freedom of speech, defection from the rules of the game, and willingness to vote for anti-democratic candidates. We first created a latent variable for political orientation, comprised of liberal-conservative ideological self-placement and strength of Republican (vs. Democratic) Party identification. Next, we tested the direct, indirect, and total effects of political orientation, RWA, SDO, and PSJ on the five outcome variables. Path coefficients were accepted as significant at the .05 level, and nonsignificant mediation paths were trimmed.

**Fig. 1:**
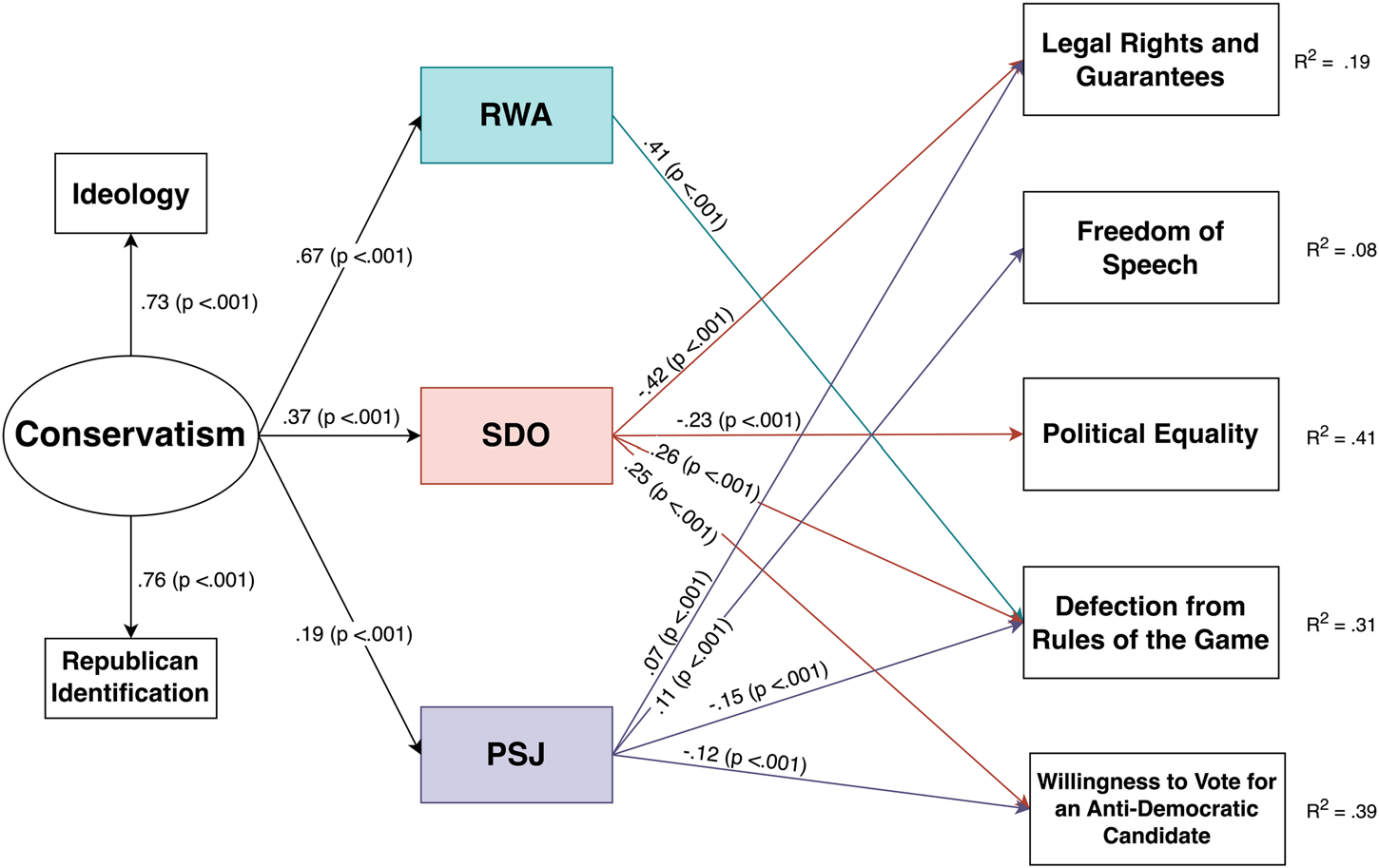
Illustration of structural equation model in which psychological variables mediated the effects of political conservatism on pro- vs. anti-democratic tendencies. Schematic representation of the mediation model, with RWA, SDO, and PSJ mediating the effects of conservatism on different pro- and anti-democratic tendencies. Values correspond to standardized estimates obtained as direct effects for each pathway and the p-values are in parentheses. R^2^ values are placed on the right of each pro- and anti-democratic tendency outcome. RWA Right-wing authoritarianism, SDO Social dominance orientation, PSJ Political system justification. *N* = 1557.

The resulting model is illustrated in Fig. 1, which provides the standardized estimates obtained as direct effects for each pathway. Because of the large sample size (*N* = 1557), chi-square was significant χ^2^ (12) = 38.438, *p* < 0.001. All other goodness-of-fit indices were very good: Comparative Fit Index (CFI) = 0.995; Tucker-Lewis Index (TLI) = 0.980; Standardized Root Mean Square Residual (SRMR) = 0.012; Root Mean-Square Error of Approximation (RMSEA) = 0.038. Table 5 displays total effects, direct effects of political orientation, and indirect effects for each psychological factor analyzed (for full results of mediation analysis, see Supplementary Note 3: Supplementary Table 51).

**Table 5:** Standardized total, direct, and indirect effects for the mediation analysis

Outcome	Model pathways	β	*P*	95% CI
Legal rights and guarantees	Total effect	−0.166	<0.001	[−0.20, −0.11]
	Direct effect of conservatism	−0.023	0.421	[−0.07, 0.03]
	Indirect effect of SDO	−0.156	<0.001	[−0.18, −0.12]
	Indirect effect of PSJ	0.014	0.002	[0.01, 0.02]
Freedom of speech	Total effect	0.265	<0.001	[0.17, 0.28]
	Direct effect of conservatism	0.244	<0.001	[0.16, 0.26]
	Indirect effect of PSJ	0.021	0.001	[0.01, 0.03]
Political equality	Total effect	−0.607	<0.001	[−0.57, −0.48]
	Direct effect of conservatism	−0.523	<0.001	[−0.49, −0.40]
	Indirect effect of SDO	−0.085	<0.001	[−0.09, −0.05]
Defection from rules of the game	Total effect	0.321	<0.001	[0.21, 0.30]
	Direct effect of conservatism	−0.023	0.601	[−0.08, 0.05]
	Indirect effect of RWA	0.277	<0.001	[0.18, 0.26]
	Indirect effect of SDO	0.096	<0.001	[0.06, 0.09]
	Indirect effect of PSJ	−0.030	<0.001	[−0.03, −0.01]
Willingness to vote for anti-democratic candidates	Total effect	0.566	<0.001	[0.47, 0.57]
	Direct effect of conservatism	0.498	<0.001	[0.41, 0.52]
	Indirect effect of SDO	0.091	<0.001	[0.06, 0.10]
	Indirect effect of PSJ	−0.024	<0.001	[−0.03, −0.01]

Brackets contain 95% confidence intervals. *N* = 1557 observations.

*RWA* Right-wing authoritarianism, *SDO* Social dominance orientation, *PSJ* Political system justification.

As shown in Fig. 1, respondents with a more politically conservative orientation were indeed higher in RWA, SDO, and PSJ. RWA was positively associated with willingness to defect from the rules of the game and a significant mediator of the effect of conservatism, consistent with (H1). SDO was positively associated with willingness to defect from the democratic rules of the game and to vote for blatantly anti-democratic candidates, and it was negatively associated with support for political equality and legal rights and guarantees. In all these cases, SDO was a significant mediator of the effect of conservatism, consistent with (H2). Thus, we obtained consistent evidence that conservatives harbored more anti-democratic tendencies than liberals and that these differences were attributable in part to the fact that they were higher in RWA and SDO.

At the same time, conservatives were also higher than liberals on PSJ, and this variable exerted an attenuating influence on anti-democratic tendencies. PSJ was positively associated with support for freedom of speech as well as legal rights and guarantees and negatively associated with willingness to defect from the rules of the game and to vote for anti-democratic candidates. In all these cases, PSJ was a significant mediator of the effect of conservatism, consistent with (H3). Thus, whereas RWA and SDO contribute to more pronounced anti-democratic tendencies among conservatives, PSJ appears to have the opposite effect.

According to the Dual Process Motivational Model, individual differences in RWA and SDO contribute to the adoption of conservative political attitudes^59^. This theoretical framework, therefore, would suggest a different Structural Equation Model than the one shown in Fig. 1. To explore this alternative model, we conducted additional analyses, which are described in the Supplementary Discussion 2 (Supplementary Fig. 2 and Supplementary Table 53). The upshot is that political conservatism significantly mediated the effects of SDO on the rejection of political equality and willingness to vote for anti-democratic candidates. It also mediated the effect of PSJ on support for freedom of speech. In addition, we also conducted additional mediation analyses adjusting for political extremism, which are presented in the Supplementary Discussion 3 (Supplementary Tables 54 and 55). Results indicated that adjusting for the effects of ideological and partisan extremism did not substantially change any of the interpretations.

### Analysis of respondents who approved vs. disapproved of the January 6 insurrectionists

We observed that 27.5% of Republican respondents (*N* = 158) and 5.96% of Democratic respondents (*N* = 40) held warm attitudes toward the January 6 insurrectionists. To explore political and psychological differences between those who approved (vs. disapproved) of the insurrectionists, we conducted within-party tests of group differences in means.

**Fig. 2:**
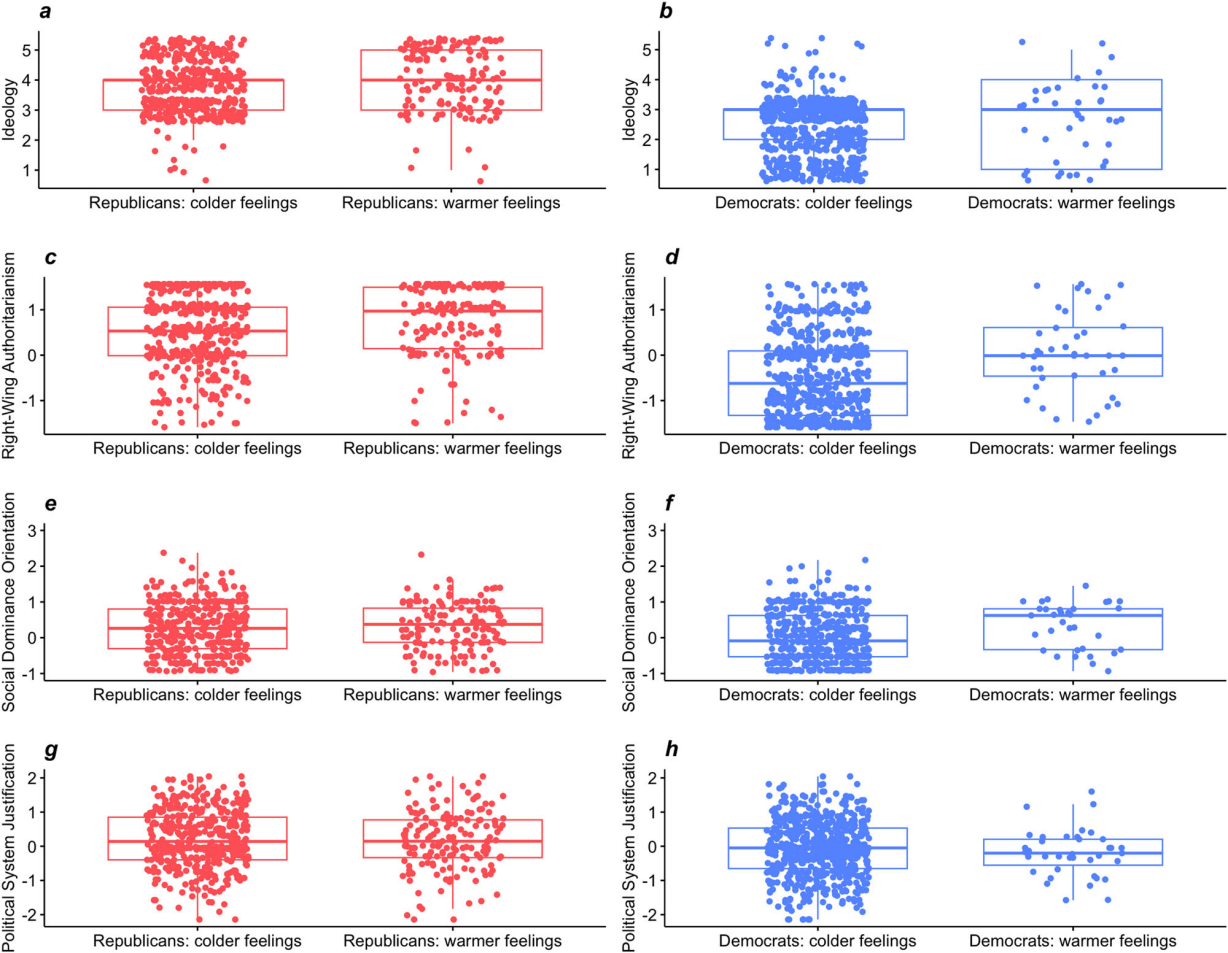
Ideology and psychological variables as a function of party affiliation and approval (vs. disapproval) of the January 6, 2021, insurrectionists. Distributions for ideology (**a** and **b**), right-wing authoritarianism (**c** and **d**), social dominance orientation (**e** and **f**), and system justification (**g** and **h**) as a function of party affiliation and approval (vs. disapproval) of January 6th, 2021, insurrectionists. Boxplots for those who identify with the Republican party are colored red and placed on the left column (*N* = 597). Boxplots for those who identify with the Democratic party are colored blue and placed on the right column (*N* = 727). Boxplots include the median in bold central line, the box representing the interquartile range, whiskers indicating 1.5 × interquartile range, and jitter dots represent data points distribution with random noise added to reduce overlapping.

Results shown in Fig. 2 revealed that Republican respondents who felt warmly toward the January 6 insurrectionists were more conservative (*Z* = 3.65, *p* < 0.001, *r* = 0.153) and higher in RWA (*Z* = 3.57, *p* < 0.001, *r* = 0.150) than Republicans who did not feel warmly toward the insurrectionists. However, we did not find evidence for statistically significant differences between the groups in terms of SDO (*Z* = 0.64, *p* = 0.520, *r* = 0.027) or PSJ (*Z* = 0.25, *p* = 0.801, *r* = 0.011). Democratic respondents who felt warmly toward the January 6 insurrectionists were also higher in RWA (*Z* = 3.69, *p* < 0.001, *r* = 0.143) and SDO (*Z* = 2.69, *p* = 0.007, *r* = 0.104) than Democrats who did not. There was no evidence for statistically significant differences among Democrats in terms of ideology (*Z* = 1.75, *p* = 0.08, *r* = 0.068) or PSJ (*Z* = 1.36, *p* = 0.175, *r* = 0.052). Although these effect sizes were not very large, we obtained consistent evidence in support of (H4), mixed evidence with respect to (H5), and no evidence in support of (H6).

To investigate the simultaneous effects of political and psychological variables, we performed hierarchical logistic regressions for the dichotomous outcome variable of approval vs. disapproval of the January 6 insurrectionists. We followed the same six steps shown in Tables 3 and 4. The results of the binomial regression are summarized in Table 6 (for complete regression results, see Supplementary Note 2: Supplementary Tables 45–50).

**Table 6:** Results of hierarchical GLM binomial regression models for approval of the January 6 insurrectionists

Independent variables	Step 1			Step 2			Step 3			Step 4			Step 5			Step 6		
	β (SE)		Std. 95% CI	*B (SE)*	*p*	Std. 95% CI	β (SE)	*p*	Std. 95% CI	β (SE)	*p*	Std. 95% CI	β (SE)	*p*	Std. 95% CI	β (SE)	p	Std. 95% CI
White	1.08 (0.26)	0.545	[0.84, 1.37]				1.00 (0.28)	0.978	[0.77, 1.29]	1.00 (0.30)	0.971	[0.76, 1.28]	1.06 (0.29)	0.646	[0.81, 1.37]	1.05 (0.29)	0.718	[0.80, 1.35]
Black	0.96 (0.32)	0.680	[0.78, 1.17]				1.08 (0.33)	0.482	[0.87, 1.33]	1.06 (0.33)	0.580	[0.86, 1.31]	1.08 (0.34)	0.480	[0.87, 1.34]	1.06 (0.34)	0.592	[0.85, 1.31]
Latino	1.04 (0.31)	0.716	[0.83, 1.29]				1.06 (0.33)	0.592	[0.85, 1.33]	1.05 (0.33)	0.666	[0.84, 1.32]	1.09 (0.34)	0.468	[.86, 1.37]	1.08 (0.34)	0.528	[0.85, 1.35]
Age	0.66 (0.00)	<0.001	[0.57, 0.76]				0.61 (0.00)	<0.001	[0.53, 0.71]	0.59 (0.00)	<0.001	[0.51, 0.69]	0.59 (0.00)	<0.001	[.51, .69]	0.58 (0.00)	<0.001	[0.49, 0.68]
Sex (1 = male, 2 = female)	1.03 (0.13)	0.684	[0.90, 1.17]				1.06 (0.14)	0.417	[0.92, 1.21]	1.04 (0.14)	0.573	[0.91, 1.19]	1.05 (0.14)	0.490	[.91, 1.21]	1.03 (0.14)	0.705	[0.89, 1.18]
Income	0.84 (0.03)	0.021	[0.72, 0.97]				0.82 (0.03)	0.012	[0.70, 0.96]	0.80 (0.03)	0.005	[0.68, 0.93]	0.82 (0.03)	0.012	[.70, .96]	0.80 (0.03)	.006	[0.68, 0.94]
Education	0.74 (0.07)	<0.001	[0.64, 0.86]				0.83 (0.07)	0.017	[0.71, 0.97]	0.83 (0.07)	0.018	[0.71, 0.97]	0.90 (0.07)	0.198	[.77, 1.06]	0.90 (0.07)	0.199	[0.77, 1.06]
Conservatism				1.17 (0.07)	0.068	[.99, 1.37]	1.17 (0.08)	0.092	[0.97, 1.40]	1.12 (0.08)	0.242	[0.93, 1.35]	1.01 (0.09)	0.945	[.83, 1.22]	0.98 (0.09)	0.804	[0.80, 1.19]
Republican ID				1.72 (0.04)	<0.001	[1.45, 2.05]	1.82 (0.04)	<0.001	[1.50, 2.20]	1.99 (0.05)	<0.001	[1.62, 2.47]	1.65 (0.05)	<0.001	[1.35, 2.02]	1.80 (0.05)	<0.001	[1.45, 2.25]
Ideological extremism										0.87 (0.09)	0.069	[0.75, 1.01]				0.89 (0.10)	0.134	[0.76 1.04]
Partisan extremism										1.23 (0.08)	0.012	[1.05, 1.44]				1.22 (0.08)	0.015	[1.04, 1.44]
RWA													1.22 (0.09)	0.021	[1.03, 1.46]	1.21 (0.09)	0.031	[1.02, 1.44]
SDO													1.37 (0.10)	<0.001	[1.17, 1.60]	1.37 (0.10)	<0.001	[1.17, 1.61]
PSJ													1.12 (0.09)	0.133	[0.97, 1.29]	1.09 (0.09)	0.249	[.94, 1.26]
*N*	1454			1440			1430			1430			1390			1390		
Pseudo R^2^	0.08			0.10			0.17			0.18			0.20			0.21		

Brackets contain standardized 95% confidence intervals.

*RWA* Right-wing authoritarianism, *SDO* Social dominance orientation, *PSJ* Political system justification.

As shown in Step 1, older respondents, wealthier respondents, and more educated respondents were more approving of the January 6 insurrectionists. In Step 2, those identified with the Republican Party were more likely to approve of the January 6 insurrectionists; this finding remained significant after adjusting for demographic variables (Step 3) and political extremism (Step 4). In Step 4, partisan extremists were more approving of the January 6 insurrectionists.

In Step 5, those identified with the Republican Party were still more likely to approve of the January 6 insurrectionists, but these effects were weaker in Step 5 than in Steps 2, 3, and 4, suggesting that the effect of partisanship may be partially attributable to demographic and/or psychological variables. Individuals who scored higher on RWA and SDO were also more approving of the insurrectionists, but no statistically significant effects were found for PSJ. After adjusting for all other variables, effects of RWA and SDO remained statistically significant.

## Discussion

The rise of political polarization and anti-democratic forms of hostility in the U.S. draws special attention because democratic support in the country used to be taken for granted both culturally and institutionally. In this research program, we sought to investigate left-right ideological asymmetries in pro- vs. anti-democratic sentiments in a nationally representative sample of U.S. adults. We also investigated whether ideological differences in anti-democratic tendencies could be explained by individual differences in psychological variables such as RWA, SDO, and PSJ^39^.

One strength of the present study is that we were able to investigate seven different indicators of pro- vs. anti-democratic sentiment ranging from adherence to general principles of liberal democracy (such as individual rights, liberties, and justice) to acceptance of explicitly anti-democratic activity (such as willingness to vote for blatantly anti-democratic candidates and engage in political violence against adversaries). This heterogeneity in terms of outcome variables enabled us to identify which specific aspects of support for democracy are affected by political orientation and psychological differences. Results of our survey revealed that, as hypothesized, self-identified conservatives (vs. liberals) and strongly identified Republicans (vs. Democrats) exhibited less support for political equality and legal rights and guarantees and greater willingness to defect from the rules of the game and to vote for blatantly anti-democratic candidates. Moreover, these effects were observed even in models that adjusted for both ideological and partisan extremism, thereby disconfirming the notion that, in the U.S. at least, extremists of the left and right are equally anti-democratic^3,7,19^. On the contrary, we observed clear evidence of an ideological asymmetry in adherence to democratic norms and principles^39^.

Most (but not all) of the observed associations between left-right political orientation and the expression of pro- vs. anti-democratic sentiment were mediated by individual differences in psychological variables. For example, RWA and SDO significantly mediated the negative effect of political conservatism on support for legal rights and guarantees and the positive effects of conservatism on willingness to defect from the rules of the game and to vote for anti-democratic candidates. All these findings were consistent with hypotheses derived from the research literature in political psychology^39,47–52^.

While RWA and SDO help to explain why anti-democratic tendencies are generally stronger among conservatives than liberals, the effect of PSJ was different. Mediational analyses indicated that conservatism was positively associated with PSJ, which was positively associated with support for free speech and legal rights and guarantees and negatively associated with willingness to vote for anti-democratic candidates and to defect from the rules of the game. In all these cases, PSJ was a significant mediator. These findings are consistent with previous evidence that system justification is linked to preferences for the political status quo^54^ and for establishment (vs. radical, anti-establishment) leaders and parties^55^. Thus, political system justification appears to play a meaningful role in maintaining popular consent with respect to democratic norms and traditions^see also 60^.

We explored similarities and differences between respondents who expressed warm feelings about the January 6, 2021, insurrectionists and those who did not. We observed that RWA and SDO were significant and robust predictors of support for the insurrectionists. Democrats and Republicans who approved of them were more conservative and higher in RWA than those who disapproved. Democrats who approved were also higher in SDO than Democrats who disapproved. However, these findings should be taken with a grain of salt, because they are based on small subsamples of Republicans and (especially) Democrats who approved of the insurrectionists.

### Limitations

There are other limitations to this study as well. For one thing, the results are based on a cross-sectional survey, so we cannot speak to causal dynamics at all. Although the Structural Equation Model illustrates possible causal pathways among variables, and our supplemental analyses testing the Dual Process Motivational Model may help to illuminate the different motivational processes underlying attitudes toward democracy, there are inferential limits that only experimental or longitudinal designs could overcome. Second, our analysis is obviously confined to American politics. Although polarization and animosity have risen very sharply in the U.S.^2^, it is certainly not the only country experiencing these phenomena^61^. The institutional conditions in the U.S. are also unique, with a two-party arrangement in the context of a majoritarian representative democracy. It would be important to investigate ideological symmetries and asymmetries—as well as the role of psychological factors such as authoritarianism, social dominance, and system justification—in other highly polarized contexts with different institutional conditions, such as proportional systems, multiparty designs, or hybrid regimes. Comparative research designs would be especially useful for exploring anti-democratic orientations that arise in both high- and low-polarization contexts. A third, related limitation is that we focused specifically on adherence to the existing liberal-democratic system in the U.S. and its foundational principles. We did not explore attitudes toward explicitly non-democratic political regimes. It is quite possible that respondents did not fully realize—and may well have denied—that they were embracing anti-democratic norms, principles, and political candidates^62^.

Despite these limitations, the results we have described testify to the relevance of a political psychological analysis of citizens’ tendencies to cooperate vs. defect from democratic norms and practices in the U.S. In particular, our study provides clear evidence of ideological asymmetries in pro- vs. anti-democratic tendencies, consistent with the theory of political conservatism as motivated social cognition^39^. Most of the findings contradict the notion that “both sides”—that is liberal-leftists and conservative-rightists—are equally to blame for the erosion of democratic norms in contemporary American politics^63–67^. This also means they cannot be explained purely in terms of generic, content-free principles of in-group identification or out-group hostility^16,32,35,38^. It would appear that liberals’ value commitments to equality, tolerance, diversity, and democracy still do count for something in American politics, at least for the time being^39^.

### Supplementary information

Supplementary Information

Peer Review File

Reporting Summary

## Data Availability

The source data underlying the figures in the main manuscript and Supplementary Information is available at Open Science Framework^68^. The DOI to access the code is 10.17605/OSF.IO/92EZY. The permanent weblink is https://osf.io/92ezy/. Full dataset for the Health of Democracy Survey is privately owned by the Rooney Center at the University of Notre Dame and is not yet publicly available, but it will be made publicly available online in April 2025 through a website for the Health of Democracy Survey.

## Code availability

All code for data analyses in R 4.2.2 associated with this manuscript is available at Open Science Framework^68^. The DOI to access the code is 10.17605/OSF.IO/92EZY. The permanent weblink is https://osf.io/92ezy/.
