# Supplementary material for: Liberal-conservative asymmetries in anti-democratic tendencies are partly explained by psychological differences in a nationally representative U.S. sample
Source: Commun Psychol. 2024 Jul 2;2:61. doi: 10.1038/s44271-024-00096-3 (PMC11332046; doi:10.1038/s44271-024-00096-3)
Supplement: Supplementary file 1 — Supplementary Information [file 44271_2024_96_MOESM1_ESM.docx]

**Liberal-conservative asymmetries in anti-democratic tendencies are partly explained by psychological differences in a nationally representative U.S. sample**

**Supplementary Information**

Débora de Oliveira Santos
Federal University of Rio Grande do Sul, Brazil

John T. Jost

New York University, USA

**Supplementary Methods**

**Supplementary Table 1: Scale items and internal consistency**

| **Variables** | **Items** | **Internal consistency** |
| --- | --- | --- |
| RWA | What our country really needs instead of more ‘civil rights’ is a good stiff dose of law and order.  What our country really needs is a strong, determined President who will crush the evil and set us on our right way again.  There is no such crime to justify the death penalty. | Cronbach’s alpha = .659 |
| SDO | An ideal society requires some groups to be on top and others to be on the bottom.  Some groups of people are simply inferior to other groups.  No one group should dominate in society (reverse-scored).  Groups at the bottom are just as deserving as groups at the top (reverse-scored). | Cronbach’s alpha = .681 |
| PSJ | The American political system is the best system there is.  Radical changes should be made in order to have a truly democratic political system in our country (reverse-scored).  The American political system is unfair and cannot be trusted (reverse-scored).  The system of checks and balances ensures that no one branch of government can ever pursue unreasonable or illegal activities. | Cronbach’s alpha = .679 |
| Legal Rights and Guarantees | The president should not be above the law.  The law should treat everyone the same, regardless of wealth or power.  Men and women should have the same legal rights and protections.  The government should never treat members of one religion differently than members of any other religion.  In order for a leader's actions to be legitimate, they need to follow the rules. | Cronbach’s alpha = .824 |
| Freedom of Speech | Everyone should be allowed to express any idea, even potentially dangerous ideas.  The government should never shut down media outlets, even if they spread disinformation.  No idea is dangerous enough to justify censorship. | Cronbach’s alpha = .708 |
| Political Equality | Everyone should be allowed to vote.  Voting should be easy.  Laws need to protect minority groups when society makes them vulnerable. | Cronbach’s alpha = .693 |
| Defection from Rules of the Game | I don't mind a politician’s methods if they manage to get the right things done.  The main trouble with democracy is that most people don’t really know what’s best for them.  The true American way of life is disappearing so fast that we may have to use force to save it. | Cronbach’s alpha = .609 |
| Willingness to Vote for Anti-democratic Candidate | My opponent is so dangerous that they deserve to go to jail.  If I lose this election, it is only because the system is rigged. I will never concede defeat to my opponent.  I don't care if the courts say this election is legitimate. I will decide whether to accept the results or not. | Cronbach’s alpha = .759 |
| Tolerance of Disliked Groups | [Least liked group] should be allowed to organize a rally in my community.  [Least liked group] should be allowed to teach in a college or university.  A book written by a member of [Least liked group] should be allowed in our local library. | Cronbach’s alpha = .864 |
| Willingness to Use Political Violence | I support the use of violence to ensure a (if Democrat, show Democratic; if Republican, show Republican; if Independent, show Democratic) party candidate wins the 2024 presidential election.  I would personally be willing to use violence to ensure that a (if Democrat, show Democratic; if Republican, show Republican; if Independent, show Democratic) party candidate wins the 2024 presidential election. | Cronbach’s alpha = .883 |

*Note*: Variables used in the analyses, the scale items corresponding to them, and their respective internal consistency results (Cronbach alpha). It is worth noting that: a) one items from the battery "Support for democratic principles" was not pertinent for any factor loadings and it was not included on the composite variables created based on factor scores (i.e. "The more people participate in politics and elections, the better"); and b) items that captured beliefs on a U.S. crisis (i.e. "The American way of life is under attack"; "The United States is on the brink of a new civil war") generated in the two-factor solution for the battery "Beliefs about American political crisis" are not included in here because (as noted by an anonymous reviewer), this variable was not unambiguously related to anti-democratic tendencies. RWA = Right-wing authoritarianism; SDO = Social dominance orientation; PSJ = Political system justification. N = 1,557.

**Supplementary Note 1: Least liked group**

**Supplementary Table 2: Least liked groups by party affiliation**

|  | Democrats | Republicans |
| --- | --- | --- |
| Black Lives Matter (BLM) supporters | 2.7% | 17.4% |
| Make America Great Again (MAGA) supporters | 27.7% | 2.6% |
| Atheists | 6.6% | 6.4% |
| Racists | 30.6% | 21.3% |
| Muslim Extremists | 32.4% | 52.3% |

*Note*: Percentages for least liked groups as a function of party affiliation. Percentages displayed refer to valid responses (N = 1,196), and missing values (N = 361) are not accounted for here.

**Supplementary Note 2: Full regression tables for pro- and anti-democratic tendencies**

**Supplementary Table 3: Full Regression Table for Step 1 - Legal Rights and Guarantees**

|  | **b** | **SE** | **β** | **t** | **p** | **Unstandardized 95% CI** | **Standardized 95% CI** |
| --- | --- | --- | --- | --- | --- | --- | --- |
| Constant | -1.26 | 0.16 |  | -7.68 | <0.001 | [-1.58,  -0.94] | [-0.05,  0.05] |
| White | 0.11 | 0.10 | 0.06 | 1.13 | 0.258 | [-0.08,  0.30] | [-0.04,  0.16] |
| Black | -0.07 | 0.12 | -0.02 | -0.54 | 0.592 | [-0.31,  0.17] | [-0.11,  0.06] |
| Latino | -0.22 | 0.12 | -0.09 | -1.83 | 0.068 | [-0.46,  0.02] | [-0.18,  0.01] |
| Age | 0.01 | 0.00 | 0.20 | 7.98 | <0.001 | [0.01,  0.01] | [0.15,  0.25] |
| Sex (1=male, 2=female) | 0.03 | 0.05 | 0.01 | 0.59 | 0.556 | [-0.06,  0.12] | [-0.03,  0.06] |
| Income | 0.04 | 0.01 | 0.09 | 3.10 | 0.002 | [0.01,  0.06] | [0.03,  0.15] |
| Education | 0.13 | 0.02 | 0.15 | 5.70 | <0.001 | [0.08,  0.17] | [0.10,  0.20] |
| N | | | | | | 1,490 | |
| R2 | | | | | | 0.126 | |
| Adjusted R2 | | | | | | 0.122 | |
| Residual SE | | | | | | 0.869  (df = 1482) | |
| F Statistic | | | | | | 30.488***  (df = 7; 1482) | |

**Supplementary Table 4: Full Regression Table for Step 2 - Legal Rights and Guarantees**

|  | **b** | **SE** | **β** | **t** | **p** | **Unstandardized 95% CI** | **Standardized 95% CI** |
| --- | --- | --- | --- | --- | --- | --- | --- |
| Constant | 0.42 | 0.05 |  | 7.71 | <0.001 | [0.31,  0.52] | [-0.05,  0.05] |
| Conservatism | -0.12 | 0.03 | -0.16 | -4.68 | <0.001 | [-0.18,  -0.07] | [-0.23,  -0.09] |
| Republican ID | -0.00 | 0.01 | -0.01 | -0.32 | 0.749 | [-0.03,  0.02] | [-0.08,  0.06] |
| N | | | | | | 1,472 | |
| R2 | | | | | | 0.028 | |
| Adjusted R2 | | | | | | 0.027 | |
| Residual SE | | | | | | 0.880  (df = 1469) | |
| F Statistic | | | | | | 21.449***  (df = 2; 1469) | |

**Supplementary Table 5: Full Regression Table for Step 3 - Legal Rights and Guarantees**

|  | **b** | **SE** | **β** | **t** | **p** | **Unstandardized 95% CI** | **Standardized 95% CI** |
| --- | --- | --- | --- | --- | --- | --- | --- |
| Constant | -0.68 | 0.16 |  | -4.19 | <0.001 | [-0.99,  -0.36] | [-0.05,  0.05] |
| White | 0.13 | 0.10 | 0.07 | 1.35 | 0.176 | [-0.06,  0.32] | [-0.03,  0.17] |
| Black | -0.05 | 0.11 | -0.02 | -0.42 | 0.677 | [-0.27,  0.17] | [-0.10,  0.06] |
| Latino | -0.24 | 0.12 | -0.10 | -2.04 | 0.042 | [-0.48,  -0.01] | [-0.19,  -0.00] |
| Age | 0.01 | 0.00 | 0.22 | 8.87 | <0.001 | [0.01,  0.01] | [0.17,  0.27] |
| Sex (1=male, 2=female) | 0.02 | 0.04 | 0.01 | 0.35 | 0.726 | [-0.07,  0.10] | [-0.04,  0.06] |
| Income | 0.04 | 0.01 | 0.09 | 3.17 | 0.002 | [0.01,  0.06] | [0.04,  0.15] |
| Education | 0.09 | 0.02 | 0.10 | 4.10 | <0.001 | [0.05,  0.13] | [0.05,  0.15] |
| Conservatism | -0.13 | 0.03 | -0.16 | -4.90 | <0.001 | [-0.18,  -0.08] | [-0.23,  -0.10] |
| Republican ID | -0.02 | 0.01 | -0.05 | -1.35 | 0.178 | [-0.05,  0.01] | [-0.12,  0.02] |
| N | | | | | | 1,463 | |
| R2 | | | | | | 0.152 | |
| Adjusted R2 | | | | | | 0.146 | |
| Residual SE | | | | | | 0.826  (df = 1453) | |
| F Statistic | | | | | | 28.86***  (df = 9; 1453) | |

**Supplementary Table 6: Full Regression Table for Step 4 - Legal Rights and Guarantees**

|  | **b** | **SE** | **β** | **t** | **p** | **Unstandardized 95% CI** | **Standardized 95% CI** |
| --- | --- | --- | --- | --- | --- | --- | --- |
| Constant | -0.76 | 0.16 |  | -4.63 | <0.001 | [-1.08,  -0.44] | [-0.05,  0.05] |
| White | 0.09 | 0.09 | 0.05 | 0.99 | 0.322 | [-0.09,  0.28] | [-0.05,  0.15] |
| Black | -0.08 | 0.11 | -0.03 | -0.73 | 0.467 | [-0.30,  0.14] | [-0.11,  0.05] |
| Latino | -0.27 | 0.12 | -0.11 | -2.28 | 0.023 | [-0.50,  -0.04] | [-0.20,  -0.02] |
| Age | 0.01 | 0.00 | 0.21 | 8.15 | <0.001 | [0.01,  0.01] | [0.16,  0.26] |
| Sex (1=male, 2=female) | 0.01 | 0.04 | 0.00 | 0.18 | 0.861 | [-0.08,  0.09] | [-0.04,  0.05] |
| Income | 0.03 | 0.01 | 0.08 | 2.94 | 0.003 | [0.01,  0.06] | [0.03,  0.14] |
| Education | 0.08 | 0.02 | 0.10 | 3.99 | <0.001 | [0.04,  0.13] | [0.05,  0.15] |
| Conservatism | -0.12 | 0.03 | -0.16 | -4.82 | <0.001 | [-0.18,  -0.07] | [-0.23,  -0.10] |
| Republican ID | -0.02 | 0.01 | -0.04 | -1.19 | 0.235 | [-0.05,  0.01] | [-0.11,  0.03] |
| Ideological extremism | 0.05 | 0.03 | 0.05 | 1.82 | 0.069 | [-0.00,  0.11] | [-0.00,  0.10] |
| Partisan extremism | 0.07 | 0.03 | 0.08 | 2.66 | 0.008 | [0.02,  0.12] | [0.02,  0.14] |
| N | | | | | | 1,463 | |
| R2 | | | | | | 0.163 | |
| Adjusted R2 | | | | | | 0.157 | |
| Residual SE | | | | | | 0.820  (df = 1451) | |
| F Statistic | | | | | | 25.78***  (df = 11; 1451) | |

**Supplementary Table 7: Full Regression Table for Step 5 - Legal Rights and Guarantees**

|  | **b** | **SE** | **β** | **t** | **p** | **Unstandardized 95% CI** | **Standardized 95% CI** |
| --- | --- | --- | --- | --- | --- | --- | --- |
| Constant | -0.65 | 0.15 |  | -4.18 | <0.001 | [-0.95,  -0.34] | [-0.04,  0.04] |
| White | 0.08 | 0.09 | 0.04 | 0.87 | 0.382 | [-0.10,  0.25] | [-0.05,  0.13] |
| Black | -0.06 | 0.11 | -0.02 | -0.54 | 0.590 | [-0.27,  0.15] | [-0.10,  0.06] |
| Latino | -0.24 | 0.11 | -0.10 | -2.19 | 0.029 | [-0.46,  -0.02] | [-0.18,  -0.01] |
| Age | 0.01 | 0.00 | 0.18 | 7.51 | <0.001 | [0.01,  0.01] | [0.14,  0.23] |
| Sex (1=male, 2=female) | 0.01 | 0.04 | 0.01 | 0.34 | 0.733 | [-0.07,  0.10] | [-0.04,  0.06] |
| Income | 0.03 | 0.01 | 0.07 | 2.64 | 0.009 | [0.01,  0.05] | [0.02,  0.13] |
| Education | 0.07 | 0.02 | 0.08 | 3.29 | 0.001 | [0.03,  0.11] | [0.03,  0.13] |
| Conservatism | -0.07 | 0.02 | -0.09 | -2.68 | 0.007 | [-0.11,  -0.02] | [-0.15,  -0.02] |
| Republican ID | -0.01 | 0.01 | -0.02 | -0.55 | 0.581 | [-0.04,  0.02] | [-0.09,  0.05] |
| RWA | 0.05 | 0.03 | 0.05 | 1.64 | 0.101 | [-0.01,  0.10] | [-0.01,  0.11] |
| SDO | -0.40 | 0.03 | -0.37 | -12.49 | <0.001 | [-0.46,  -0.34] | [-0.43,  -0.31] |
| PSJ | 0.01 | 0.02 | 0.01 | 0.39 | 0.694 | [-0.04 – 0.06] | [-0.04,  0.06] |
| N | | | | | | 1,426 | |
| R2 | | | | | | 0.265 | |
| Adjusted R2 | | | | | | 0.259 | |
| Residual SE | | | | | | 0.760  (df = 1413) | |
| F Statistic | | | | | | 42.46***  (df = 12; 1413) | |

**Supplementary Table 8: Full Regression Table for Step 6 - Legal Rights and Guarantees**

|  | **b** | **SE** | **β** | **t** | **p** | **Unstandardized 95% CI** | **Standardized 95% CI** |
| --- | --- | --- | --- | --- | --- | --- | --- |
| Constant | -0.71 | 0.16 |  | -4.53 | <0.001 | [-1.02,  -0.40] | [-0.04,  0.04] |
| White | 0.05 | 0.09 | 0.03 | 0.61 | 0.543 | [-0.12,  0.22] | [-0.06,  0.12] |
| Black | -0.08 | 0.11 | -0.03 | -0.79 | 0.430 | [-0.29,  0.13] | [-0.11,  0.05] |
| Latino | -0.26 | 0.11 | -0.10 | -2.36 | 0.018 | [-0.47,  -0.04] | [-0.19,  -0.02] |
| Age | 0.01 | 0.00 | 0.17 | 7.01 | <0.001 | [0.01,  0.01] | [0.12,  0.22] |
| Sex (1=male, 2=female) | 0.01 | 0.04 | 0.00 | 0.12 | 0.901 | [-0.08,  0.09] | [-0.04,  0.05] |
| Income | 0.03 | 0.01 | 0.07 | 2.46 | 0.014 | [0.01,  0.05] | [0.01,  0.12] |
| Education | 0.07 | 0.02 | 0.08 | 3.23 | 0.001 | [0.03,  0.11] | [0.03,  0.13] |
| Conservatism | -0.06 | 0.02 | -0.08 | -2.66 | 0.008 | [-0.11,  -0.02] | [-0.15,  -0.02] |
| Republican ID | -0.01 | 0.01 | -0.01 | -0.38 | 0.701 | [-0.03,  0.02] | [-0.08,  0.06] |
| Ideological extremism | 0.03 | 0.03 | 0.02 | 0.93 | 0.352 | [-0.03,  0.08] | [-0.03,  0.07] |
| Partisan extremism | 0.06 | 0.02 | 0.07 | 2.37 | 0.018 | [0.01,  0.11] | [0.01,  0.13] |
| RWA | 0.04 | 0.03 | 0.05 | 1.54 | 0.125 | [-0.01,  0.10] | [-0.01,  0.11] |
| SDO | -0.39 | 0.03 | -0.36 | -12.25 | <0.001 | [-0.45,  -0.33] | [-0.42,  -0.31] |
| PSJ | 0.00 | 0.02 | 0.00 | 0.12 | 0.904 | [-0.04,  0.05] | [-0.04,  0.05] |
| N | | | | | | 1,426 | |
| R2 | | | | | | 0.271 | |
| Adjusted R2 | | | | | | 0.264 | |
| Residual SE | | | | | | 0.758  (df = 1411) | |
| F Statistic | | | | | | 37.52***  (df = 14; 1411) | |

**Supplementary Table 9: Full Regression Table for Step 1 - Political Equality**

|  | **b** | **SE** | **β** | **t** | **p** | **Unstandardized 95% CI** | **Standardized 95% CI** |
| --- | --- | --- | --- | --- | --- | --- | --- |
| Constant | -0.58 | 0.14 |  | -4.20 | <0.001 | [-0.85,  -0.31] | [-0.05,  0.05] |
| White | -0.04 | 0.08 | -0.02 | -0.46 | 0.644 | [-0.20,  0.12] | [-0.11,  0.07] |
| Black | 0.28 | 0.10 | 0.11 | 2.94 | 0.003 | [0.09,  0.47] | [0.04,  0.18] |
| Latino | -0.10 | 0.10 | -0.04 | -1.07 | 0.283 | [-0.29,  0.09] | [-0.12,  0.04] |
| Age | 0.00 | 0.00 | 0.02 | 0.81 | 0.417 | [-0.00,  0.00] | [-0.03,  0.07] |
| Sex (1=male, 2=female) | 0.10 | 0.04 | 0.06 | 2.27 | 0.023 | [0.01,  0.19] | [0.01,  0.11] |
| Income | -0.00 | 0.01 | -0.01 | -0.23 | 0.818 | [-0.02,  0.02] | [-0.06,  0.05] |
| Education | 0.12 | 0.02 | 0.15 | 5.43 | <0.001 | [0.08,  0.17] | [0.10,  0.21] |
| N | | | | | | 1,490 | |
| R2 | | | | | | 0.042 | |
| Adjusted R2 | | | | | | 0.037 | |
| Residual SE | | | | | | 0.842  (df = 1482) | |
| F Statistic | | | | | | 9.25***  (df = 7; 1482) | |

**Supplementary Table 10: Full Regression Table for Step 2 - Political Equality**

|  | **b** | **SE** | **β** | **t** | **p** | **Unstandardized 95% CI** | **Standardized 95% CI** |
| --- | --- | --- | --- | --- | --- | --- | --- |
| Constant | 1.05 | 0.05 |  | 23.21 | <0.001 | [0.96,  1.13] | [-0.04,  0.04] |
| Conservatism | -0.20 | 0.02 | -0.27 | -9.01 | <0.001 | [-0.24,  -0.15] | [-0.33,  -0.21] |
| Republican ID | -0.12 | 0.01 | -0.29 | -9.53 | <0.001 | [-0.14,  -0.09] | [-0.35,  -0.23] |
| N | | | | | | 1,472 | |
| R2 | | | | | | 0.261 | |
| Adjusted R2 | | | | | | 0.260 | |
| Residual SE | | | | | | 0.732  (df = 1469) | |
| F Statistic | | | | | | 258.77***  (df = 2; 1469) | |

**Supplementary Table 11: Full Regression Table for Step 3 - Political Equality**

|  | **b** | **SE** | **β** | **t** | **p** | **Unstandardized 95% CI** | **Standardized 95% CI** |
| --- | --- | --- | --- | --- | --- | --- | --- |
| Constant | 0.59 | 0.13 |  | 4.43 | <0.001 | [0.33,  0.85] | [-0.04,  0.04] |
| White | 0.09 | 0.08 | 0.05 | 1.15 | 0.248 | [-0.06,  0.24] | [-0.03,  0.13] |
| Black | 0.19 | 0.09 | 0.07 | 2.13 | 0.033 | [0.01,  0.36] | [0.01,  0.14] |
| Latino | -0.11 | 0.09 | -0.05 | -1.21 | 0.227 | [-0.28,  0.07] | [-0.12,  0.03] |
| Age | 0.00 | 0.00 | 0.07 | 3.02 | 0.003 | [0.00,  0.01] | [0.02,  0.11] |
| Sex (1=male, 2=female) | 0.06 | 0.04 | 0.03 | 1.48 | 0.139 | [-0.02,  0.13] | [-0.01,  0.08] |
| Income | -0.00 | 0.01 | -0.00 | -0.15 | 0.878 | [-0.02,  0.02] | [-0.05,  0.04] |
| Education | 0.04 | 0.02 | 0.05 | 2.22 | 0.026 | [0.01,  0.08] | [0.01,  0.10] |
| Conservatism | -0.20 | 0.02 | -0.27 | -8.89 | <0.001 | [-0.25,  -0.16] | [-0.33,  -0.21] |
| Republican ID | -0.11 | 0.01 | -0.28 | -8.87 | <0.001 | [-0.14,  -0.09] | [-0.35,  -0.22] |
| N | | | | | | 1,463 | |
| R2 | | | | | | 0.280 | |
| Adjusted R2 | | | | | | 0.275 | |
| Residual SE | | | | | | 0.724  (df = 1453) | |
| F Statistic | | | | | | 62.7***  (df = 9; 1453) | |

**Supplementary Table 12: Full Regression Table for Step 4 - Political Equality**

|  | **b** | **SE** | **β** | **t** | **p** | **Unstandardized 95% CI** | **Standardized 95% CI** |
| --- | --- | --- | --- | --- | --- | --- | --- |
| Constant | 0.56 | 0.14 |  | 4.09 | <0.001 | [0.29,  0.82] | [-0.04,  0.04] |
| White | 0.07 | 0.08 | 0.04 | 0.91 | 0.362 | [-0.08,  0.22] | [-0.04,  0.12] |
| Black | 0.17 | 0.09 | 0.06 | 1.87 | 0.062 | [-0.01,  0.34] | [-0.00,  0.13] |
| Latino | -0.12 | 0.09 | -0.05 | -1.36 | 0.174 | [-0.30,  0.05] | [-0.13,  0.02] |
| Age | 0.00 | 0.00 | 0.06 | 2.47 | 0.014 | [0.00,  0.01] | [0.01,  0.10] |
| Sex (1=male, 2=female) | 0.05 | 0.04 | 0.03 | 1.28 | 0.200 | [-0.03,  0.12] | [-0.02,  0.07] |
| Income | -0.00 | 0.01 | -0.01 | -0.44 | 0.662 | [-0.02,  0.01] | [-0.06,  0.04] |
| Education | 0.04 | 0.02 | 0.05 | 2.19 | 0.029 | [0.00,  0.08] | [0.01,  0.10] |
| Conservatism | -0.20 | 0.02 | -0.28 | -8.71 | <0.001 | [-0.25,  -0.16] | [-0.34,  -0.21] |
| Republican ID | -0.11 | 0.01 | -0.28 | -8.41 | <0.001 | [-0.13,  -0.08] | [-0.34,  -0.21] |
| Ideological extremism | 0.00 | 0.03 | 0.00 | 0.03 | 0.976 | [-0.05,  0.05] | [-0.05,  0.05] |
| Partisan extremism | 0.05 | 0.02 | 0.06 | 2.34 | 0.020 | [0.01,  0.10] | [0.01,  0.12] |
| N | | | | | | 1,463 | |
| R2 | | | | | | 0.284 | |
| Adjusted R2 | | | | | | 0.278 | |
| Residual SE | | | | | | 0.722  (df = 1451) | |
| F Statistic | | | | | | 52.22***  (df = 11; 1451) | |

**Supplementary Table 13: Full Regression Table for Step 5 - Political Equality**

|  | **b** | **SE** | **β** | **t** | **p** | **Unstandardized 95% CI** | **Standardized 95% CI** |
| --- | --- | --- | --- | --- | --- | --- | --- |
| Constant | 0.48 | 0.13 |  | 3.73 | <0.001 | [0.23,  0.73] | [-0.04,  0.04] |
| White | -0.00 | 0.07 | -0.00 | -0.03 | 0.973 | [-0.13,  0.13] | [-0.07,  0.07] |
| Black | 0.13 | 0.08 | 0.05 | 1.58 | 0.113 | [-0.03,  0.30] | [-0.01,  0.11] |
| Latino | -0.16 | 0.08 | -0.07 | -1.90 | 0.058 | [-0.32,  0.01] | [-0.13,  0.00] |
| Age | 0.00 | 0.00 | 0.06 | 2.81 | 0.005 | [0.00,  0.01] | [0.02,  0.11] |
| Sex (1=male, 2=female) | 0.06 | 0.04 | 0.03 | 1.55 | 0.121 | [-0.02,  0.13] | [-0.01,  0.08] |
| Income | -0.01 | 0.01 | -0.01 | -0.62 | 0.533 | [-0.02,  0.01] | [-0.06,  0.03] |
| Education | 0.00 | 0.02 | 0.00 | 0.18 | 0.861 | [-0.03,  0.04] | [-0.04,  0.05] |
| Conservatism | -0.12 | 0.02 | -0.17 | -5.36 | <0.001 | [-0.17,  -0.08] | [-0.23,  -0.11] |
| Republican ID | -0.08 | 0.01 | -0.21 | -6.38 | <0.001 | [-0.11,  -0.06] | [-0.27,  -0.15] |
| RWA | -0.13 | 0.02 | -0.15 | -5.19 | <0.001 | [-0.18,  -0.08] | [-0.20,  -0.09] |
| SDO | -0.27 | 0.03 | -0.26 | -10.08 | <0.001 | [-0.32,  -0.21] | [-0.31,  -0.21] |
| PSJ | -0.03 | 0.02 | -0.04 | -1.55 | 0.120 | [-0.08,  0.01] | [-0.08,  0.01] |
| N | | | | | | 1,426 | |
| R2 | | | | | | 0.364 | |
| Adjusted R2 | | | | | | 0.359 | |
| Residual SE | | | | | | 0.675  (df = 1413) | |
| F Statistic | | | | | | 67.49***  (df = 12; 1413) | |

**Supplementary Table 14: Full Regression Table for Step 6 - Political Equality**

|  | **b** | **SE** | **β** | **t** | **p** | **Unstandardized 95% CI** | **Standardized 95% CI** |
| --- | --- | --- | --- | --- | --- | --- | --- |
| Constant | 0.45 | 0.13 |  | 3.46 | 0.001 | [0.20,  0.71] | [-0.04,  0.04] |
| White | -0.01 | 0.07 | -0.01 | -0.22 | 0.829 | [-0.15,  0.12] | [-0.08,  0.07] |
| Black | 0.11 | 0.08 | 0.04 | 1.31 | 0.191 | [-0.05,  0.27] | [-0.02,  0.10] |
| Latino | -0.16 | 0.08 | -0.07 | -2.00 | 0.046 | [-0.32,  0.00] | [-0.14,  - 0.00] |
| Age | 0.00 | 0.00 | 0.06 | 2.44 | 0.015 | [0.00,  0.00] | [0.01,  0.10] |
| Sex (1=male, 2=female) | 0.05 | 0.04 | 0.03 | 1.25 | 0.213 | [-0.03,  0.12] | [-0.02,  0.07] |
| Income | -0.01 | 0.01 | -0.02 | -0.93 | 0.354 | [-0.03,  0.01] | [-0.07,  0.02] |
| Education | 0.00 | 0.02 | 0.00 | 0.20 | 0.844 | [-0.03,  0.04] | [-0.04,  0.05] |
| Conservatism | -0.12 | 0.02 | -0.17 | -5.36 | <0.001 | [-0.17,  -0.08] | [-0.23,  -0.11] |
| Republican ID | -0.08 | 0.01 | -0.20 | -5.83 | <0.001 | [-0.10,  -0.05] | [-0.26,  -0.13] |
| Ideological extremism | -0.03 | 0.02 | -0.03 | -1.18 | 0.236 | [-0.08,  0.02] | [-0.08,  0.02] |
| Partisan extremism | 0.05 | 0.02 | 0.07 | 2.46 | 0.014 | [0.01,  0.09] | [0.01,  0.12] |
| RWA | -0.13 | 0.02 | -0.15 | -5.37 | <0.001 | [-0.18,  -0.08] | [-0.20,  -0.10] |
| SDO | -0.26 | 0.03 | -0.26 | -9.96 | <0.001 | [-0.31,  -0.21] | [-0.31,  -0.21] |
| PSJ | -0.04 | 0.02 | -0.04 | -1.87 | 0.062 | [-0.09,  0.00] | [-0.09,  0.00] |
| N | | | | | | 1,426 | |
| R2 | | | | | | 0.368 | |
| Adjusted R2 | | | | | | 0.361 | |
| Residual SE | | | | | | 0.674  (df = 1411) | |
| F Statistic | | | | | | 58.6***  (df = 14; 1411) | |

**Supplementary Table 15: Full Regression Table for Step 1 - Freedom of Speech**

|  | **b** | **SE** | **β** | **t** | **p** | **Unstandardized 95% CI** | **Standardized 95% CI** |
| --- | --- | --- | --- | --- | --- | --- | --- |
| Constant | 0.44 | 0.14 |  | 3.05 | 0.002 | [0.16,  0.72] | [-0.05,  0.05] |
| White | 0.04 | 0.09 | 0.02 | 0.44 | 0.660 | [-0.14,  0.22] | [-0.08,  0.12] |
| Black | -0.14 | 0.11 | -0.05 | -1.25 | 0.213 | [-0.35,  0.08] | [-0.13,  0.03] |
| Latino | 0.16 | 0.10 | 0.07 | 1.50 | 0.133 | [-0.05,  0.36] | [-0.02,  0.15] |
| Age | -0.00 | 0.00 | -0.02 | -0.90 | 0.370 | [-0.00,  0.00] | [-0.07,  0.03] |
| Sex (1=male, 2=female) | -0.23 | 0.04 | -0.13 | -5.04 | <0.001 | [-0.31,  -0.14] | [-0.18,  -0.08] |
| Income | -0.01 | 0.01 | -0.02 | -0.85 | 0.393 | [-0.03,  0.01] | [-0.08,  0.03] |
| Education | -0.01 | 0.02 | -0.01 | -0.28 | 0.782 | [-0.05,  0.04] | [-0.06,  0.05] |
| N | | | | | | 1,490 | |
| R2 | | | | | | 0.026 | |
| Adjusted R2 | | | | | | 0.021 | |
| Residual SE | | | | | | 0.847  (df = 1482) | |
| F Statistic | | | | | | 5.650***  (df = 7; 1482) | |

**Supplementary Table 16: Full Regression Table for Step 2 - Freedom of Speech**

|  | **b** | **SE** | **β** | **t** | **p** | **Unstandardized 95% CI** | **Standardized 95% CI** |
| --- | --- | --- | --- | --- | --- | --- | --- |
| Constant | -0.40 | 0.06 |  | -6.22 | <0.001 | [-0.52,  -0.27] | [-0.05,  0.05] |
| Conservatism | 0.05 | 0.03 | 0.07 | 1.91 | 0.056 | [-0.00,  0.10] | [-0.00,  0.13] |
| Republican ID | 0.07 | 0.01 | 0.17 | 5.31 | <0.001 | [0.04,  0.09] | [0.11,  0.24] |
| N | | | | | | 1,472 | |
| R2 | | | | | | 0.049 | |
| Adjusted R2 | | | | | | 0.047 | |
| Residual SE | | | | | | 0.839  (df = 1469) | |
| F Statistic | | | | | | 37.535***  (df = 2; 1469) | |

**Supplementary Table 17: Full Regression Table for Step 3 - Freedom of Speech**

|  | **b** | **SE** | **β** | **t** | **p** | **Unstandardized 95% CI** | **Standardized 95% CI** |
| --- | --- | --- | --- | --- | --- | --- | --- |
| Constant | 0.01 | 0.16 |  | 0.07 | 0.948 | [-0.30,  0.32] | [-0.05,  0.05] |
| White | -0.02 | 0.09 | -0.01 | -0.20 | 0.841 | [-0.20,  0.16] | [-0.11,  0.09] |
| Black | -0.04 | 0.11 | -0.02 | -0.36 | 0.718 | [-0.26,  0.18] | [-0.10,  0.07] |
| Latino | 0.16 | 0.10 | 0.07 | 1.56 | 0.119 | [-0.04,  0.37] | [-0.02,  0.15] |
| Age | -0.00 | 0.00 | -0.05 | -2.01 | 0.045 | [-0.01,  0.00] | [-0.10,  0.00] |
| Sex (1=male, 2=female) | -0.20 | 0.04 | -0.12 | -4.57 | <0.001 | [-0.29,  -0.12] | [-0.17,  -0.07] |
| Income | -0.01 | 0.01 | -0.03 | -1.20 | 0.232 | [-0.03,  0.01] | [-0.09,  0.02] |
| Education | 0.02 | 0.02 | 0.03 | 1.08 | 0.279 | [-0.02,  0.07] | [-0.02,  0.09] |
| Conservatism | 0.05 | 0.03 | 0.07 | 2.06 | 0.040 | [0.00,  0.10] | [0.00,  0.14] |
| Republican ID | 0.07 | 0.01 | 0.17 | 5.02 | <0.001 | [0.04,  0.10] | [0.11,  0.24] |
| N | | | | | | 1,463 | |
| R2 | | | | | | 0.072 | |
| Adjusted R2 | | | | | | 0.066 | |
| Residual SE | | | | | | 0.831  (df = 1453) | |
| F Statistic | | | | | | 12.5***  (df = 9; 1453) | |

**Supplementary Table 18: Full Regression Table for Step 4 - Freedom of Speech**

|  | **b** | **SE** | **β** | **t** | **p** | **Unstandardized 95% CI** | **Standardized 95% CI** |
| --- | --- | --- | --- | --- | --- | --- | --- |
| Constant | -0.01 | 0.16 |  | -0.05 | 0.964 | [-0.32,  0.30] | [-0.05,  0.05] |
| White | -0.02 | 0.09 | -0.01 | -0.22 | 0.824 | [-0.20,  0.16] | [-0.11,  0.09] |
| Black | -0.03 | 0.11 | -0.01 | -0.28 | 0.778 | [-0.25,  0.19] | [-0.09,  0.07] |
| Latino | 0.16 | 0.10 | 0.07 | 1.55 | 0.121 | [-0.04,  0.37] | [-0.02,  0.15] |
| Age | -0.00 | 0.00 | -0.05 | -1.80 | 0.072 | [-0.01,  0.00] | [-0.10,  0.00] |
| Sex (1=male, 2=female) | -0.19 | 0.04 | -0.11 | -4.41 | <0.001 | [-0.28,  -0.11] | [-0.16,  -0.06] |
| Income | -0.01 | 0.01 | -0.03 | -0.98 | 0.326 | [-0.03,  0.01] | [-0.08,  0.03] |
| Education | 0.02 | 0.02 | 0.03 | 1.00 | 0.319 | [-0.02,  0.07] | [-0.03,  0.08] |
| Conservatism | 0.06 | 0.03 | 0.08 | 2.22 | 0.027 | [0.01,  0.11] | [0.01,  0.15] |
| Republican ID | 0.07 | 0.01 | 0.16 | 4.67 | <0.001 | [0.04,  0.09] | [0.09,  0.23] |
| Ideological extremism | 0.06 | 0.03 | 0.06 | 1.93 | 0.054 | [-0.00,  0.12] | [-0.00,  0.11] |
| Partisan extremism | -0.03 | 0.02 | -0.04 | -1.24 | 0.217 | [-0.08,  0.02] | [-0.09,  0.02] |
| N | | | | | | 1,463 | |
| R2 | | | | | | 0.075 | |
| Adjusted R2 | | | | | | 0.068 | |
| Residual SE | | | | | | 0.830  (df = 1451) | |
| F Statistic | | | | | | 10.64***  (df = 11; 1451) | |

**Supplementary Table 19: Full Regression Table for Step 5 - Freedom of Speech**

|  | **b** | **SE** | **β** | **t** | **p** | **Unstandardized 95% CI** | **Standardized 95% CI** |
| --- | --- | --- | --- | --- | --- | --- | --- |
| Constant | 0.20 | 0.16 |  | 1.27 | 0.204 | [-0.11,  0.51] | [-0.05,  0.05] |
| White | -0.00 | 0.10 | -0.00 | -0.01 | 0.995 | [-0.19,  0.19] | [-0.10,  0.10] |
| Black | -0.04 | 0.11 | -0.01 | -0.34 | 0.735 | [-0.26,  0.19] | [-0.10,  0.07] |
| Latino | 0.15 | 0.11 | 0.06 | 1.44 | 0.151 | [-0.06,  0.36] | [-0.02,  0.15] |
| Age | -0.00 | 0.00 | -0.09 | -3.47 | 0.001 | [-0.01,  -0.00] | [-0.14,  -0.04] |
| Sex (1=male, 2=female) | -0.19 | 0.04 | -0.11 | -4.20 | <0.001 | [-0.28,  -0.10] | [-0.16,  -0.06] |
| Income | -0.02 | 0.01 | -0.04 | -1.51 | 0.132 | [-0.04,  0.00] | [-0.10,  0.01] |
| Education | 0.03 | 0.02 | 0.04 | 1.38 | 0.168 | [-0.01,  0.08] | [-0.02,  0.10] |
| Conservatism | 0.03 | 0.03 | 0.04 | 0.98 | 0.328 | [-0.03,  0.08] | [-0.04,  0.11] |
| Republican ID | 0.06 | 0.01 | 0.14 | 3.86 | <0.001 | [0.03,  0.09] | [0.07,  0.22] |
| RWA | 0.07 | 0.03 | 0.08 | 2.25 | 0.025 | [0.01,  0.13] | [0.01,  0.15] |
| SDO | -0.00 | 0.03 | -0.00 | -0.06 | 0.949 | [-0.06,  0.06] | [-0.06,  0.06] |
| PSJ | 0.14 | 0.03 | 0.14 | 4.82 | <0.001 | [0.08,  0.20] | [0.08,  0.20] |
| N | | | | | | 1,426 | |
| R2 | | | | | | 0.095 | |
| Adjusted R2 | | | | | | 0.087 | |
| Residual SE | | | | | | 0.820  (df = 1413) | |
| F Statistic | | | | | | 12.29***  (df = 12; 1413) | |

**Supplementary Table 20: Full Regression Table for Step 6 - Freedom of Speech**

|  | **b** | **SE** | **β** | **t** | **p** | **Unstandardized 95% CI** | **Standardized 95% CI** |
| --- | --- | --- | --- | --- | --- | --- | --- |
| Constant | 0.20 | 0.16 |  | 1.23 | 0.220 | [-0.12,  0.51] | [-0.05,  0.05] |
| White | 0.00 | 0.10 | 0.00 | 0.02 | 0.986 | [-0.19,  0.19] | [-0.10,  0.11] |
| Black | -0.02 | 0.12 | -0.01 | -0.17 | 0.863 | [-0.25,  0.21] | [-0.09,  0.08] |
| Latino | 0.16 | 0.11 | 0.06 | 1.45 | 0.147 | [-0.05,  0.37] | [-0.02,  0.15] |
| Age | -0.00 | 0.00 | -0.09 | -3.25 | 0.001 | [-0.01,  -0.00] | [-0.14,  -0.03] |
| Sex (1=male, 2=female) | -0.18 | 0.04 | -0.10 | -3.93 | <0.001 | [-0.26,  -0.09] | [-0.15,  -0.05] |
| Income | -0.01 | 0.01 | -0.03 | -1.19 | 0.233 | [-0.03,  0.01] | [-0.09,  0.02] |
| Education | 0.03 | 0.02 | 0.04 | 1.30 | 0.195 | [-0.02,  0.08] | [-0.02,  0.09] |
| Conservatism | 0.03 | 0.03 | 0.04 | 1.11 | 0.269 | [-0.02,  0.08] | [-0.03,  0.11] |
| Republican ID | 0.05 | 0.01 | 0.13 | 3.39 | 0.001 | [0.02,  0.08] | [0.05,  0.20] |
| Ideological extremism | 0.08 | 0.03 | 0.07 | 2.51 | 0.012 | [0.02,  0.14] | [0.02,  0.13] |
| Partisan extremism | -0.05 | 0.02 | -0.06 | -1.95 | 0.052 | [-0.09,  0.00] | [-0.12,  0.00] |
| RWA | 0.08 | 0.03 | 0.08 | 2.42 | 0.016 | [0.01,  0.14] | [0.02,  0.15] |
| SDO | -0.00 | 0.03 | -0.00 | -0.01 | 0.991 | [-0.06,  0.06] | [-0.06,  0.06] |
| PSJ | 0.15 | 0.03 | 0.15 | 5.10 | <0.001 | [0.09,  0.21] | [0.09,  0.21] |
| N | | | | | | 1,426 | |
| R2 | | | | | | 0.100 | |
| Adjusted R2 | | | | | | 0.091 | |
| Residual SE | | | | | | 0.820  (df = 1411) | |
| F Statistic | | | | | | 11.15***  (df = 14; 1411) | |

**Supplementary Table 21: Full Regression Table for Step 1 - Defection from Rules of the Game**

|  | **b** | **SE** | **β** | **t** | **p** | **Unstandardized 95% CI** | **Standardized 95% CI** |
| --- | --- | --- | --- | --- | --- | --- | --- |
| Constant | 0.87 | 0.12 |  | 7.11 | <0.001 | [0.63,  1.12] | [-0.05,  0.05] |
| White | -0.15 | 0.08 | -0.09 | -1.91 | 0.056 | [-0.31,  -0.00] | [-0.19,  0.00] |
| Black | 0.04 | 0.10 | 0.02 | 0.38 | 0.702 | [-0.15,  0.22] | [-0.06,  0.09] |
| Latino | 0.09 | 0.09 | 0.04 | 0.96 | 0.338 | [-0.09,  0.27] | [-0.04,  0.12] |
| Age | -0.00 | 0.00 | -0.05 | -2.17 | 0.031 | [-0.00,  -0.00] | [-0.10,  -0.01] |
| Sex (1=male, 2=female) | 0.02 | 0.04 | 0.01 | 0.53 | 0.593 | [-0.06,  0.10] | [-0.03,  0.06] |
| Income | -0.01 | 0.01 | -0.03 | -1.21 | 0.225 | [-0.03,  0.01] | [-0.09,  0.02] |
| Education | -0.19 | 0.02 | -0.26 | -9.81 | <0.001 | [-0.23,  -0.16] | [-0.31,  -0.21] |
| N | | | | | | 1,534 | |
| R2 | | | | | | 0.112 | |
| Adjusted R2 | | | | | | 0.108 | |
| Residual SE | | | | | | 0.750  (df = 1526) | |
| F Statistic | | | | | | 27.605***  (df = 7; 1526) | |

**Supplementary Table 22: Full Regression Table for Step 2 - Defection from Rules of the Game**

|  | **b** | **SE** | **β** | **t** | **p** | **Unstandardized 95% CI** | **Standardized 95% CI** |
| --- | --- | --- | --- | --- | --- | --- | --- |
| Constant | -0.51 | 0.06 |  | -8.82 | <0.001 | [-0.62,  -0.39] | [-0.05,  0.05] |
| Conservatism | 0.10 | 0.02 | 0.14 | 4.04 | <0.001 | [0.05,  0.15] | [0.07,  0.21] |
| Republican ID | 0.05 | 0.01 | 0.14 | 4.14 | <0.001 | [0.03,  0.08] | [0.08,  0.21] |
| N | | | | | | 1,516 | |
| R2 | | | | | | 0.068 | |
| Adjusted R2 | | | | | | 0.067 | |
| Residual SE | | | | | | 0.769  (df = 1513) | |
| F Statistic | | | | | | 55.602***  (df = 2; 1513) | |

**Supplementary Table 23: Full Regression Table for Step 3 - Defection from Rules of the Game**

|  | **b** | **SE** | **β** | **t** | **p** | **Unstandardized 95% CI** | **Standardized 95% CI** |
| --- | --- | --- | --- | --- | --- | --- | --- |
| Constant | 0.33 | 0.14 |  | 2.43 | 0.015 | [0.06,  0.59] | [-0.05,  0.05] |
| White | -0.23 | 0.08 | -0.13 | -2.82 | 0.005 | [-0.38,  -0.07] | [-0.23,  -0.04] |
| Black | 0.15 | 0.10 | 0.06 | 1.50 | 0.135 | [-0.05,  0.34] | [-0.02,  0.14] |
| Latino | 0.07 | 0.09 | 0.03 | 0.80 | 0.423 | [-0.11,  0.25] | [-0.05,  0.11] |
| Age | -0.00 | 0.00 | -0.09 | -3.65 | <0.001 | [-0.01,  -0.00] | [-0.14,  -0.04] |
| Sex (1=male, 2=female) | 0.05 | 0.04 | 0.03 | 1.34 | 0.179 | [-0.02,  0.12] | [-0.01,  0.08] |
| Income | -0.01 | 0.01 | -0.03 | -1.35 | 0.178 | [-0.03,  0.01] | [-0.09,  0.02] |
| Education | -0.16 | 0.02 | -0.21 | -8.09 | <0.001 | [-0.20,  -0.12] | [-0.26,  -0.16] |
| Conservatism | 0.08 | 0.02 | 0.11 | 3.34 | 0.001 | [0.03,  0.13] | [0.05,  0.18] |
| Republican ID | 0.07 | 0.01 | 0.20 | 5.98 | <0.001 | [0.05,  0.10] | [0.13,  0.27] |
| N | | | | | | 1,506 | |
| R2 | | | | | | 0.189 | |
| Adjusted R2 | | | | | | 0.184 | |
| Residual SE | | | | | | 0.719  (df = 1496) | |
| F Statistic | | | | | | 38.66***  (df = 9; 1496) | |

**Supplementary Table 24: Full Regression Table for Step 4 - Defection from Rules of the Game**

|  | **b** | **SE** | **β** | **t** | **p** | **Unstandardized 95% CI** | **Standardized 95% CI** |
| --- | --- | --- | --- | --- | --- | --- | --- |
| Constant | 0.34 | 0.13 |  | 2.46 | 0.014 | [0.07,  0.60] | [-0.05,  0.05] |
| White | -0.23 | 0.08 | -0.14 | -2.82 | 0.005 | [-0.39,  -0.07] | [-0.23,  -0.04] |
| Black | 0.13 | 0.10 | 0.05 | 1.37 | 0.171 | [-0.06,  0.33] | [-0.02,  0.13] |
| Latino | 0.07 | 0.09 | 0.03 | 0.76 | 0.446 | [-0.11,  0.25] | [-0.05,  0.11] |
| Age | -0.00 | 0.00 | -0.09 | -3.81 | <0.001 | [-0.01,  -0.00] | [-0.14,  -0.05] |
| Sex (1=male, 2=female) | 0.04 | 0.04 | 0.03 | 1.13 | 0.257 | [-0.03,  0.12] | [-0.02,  0.07] |
| Income | -0.01 | 0.01 | -0.04 | -1.59 | 0.112 | [-0.03,  0.00] | [-0.09,  0.01] |
| Education | -0.16 | 0.02 | -0.21 | -7.98 | <0.001 | [-0.20,  -0.12] | [-0.26,  -0.16] |
| Conservatism | 0.07 | 0.02 | 0.11 | 3.15 | 0.002 | [0.03,  0.12] | [0.04,  0.18] |
| Republican ID | 0.08 | 0.01 | 0.21 | 6.21 | <0.001 | [0.05,  0.10] | [0.14,  0.28] |
| Ideological extremism | -0.05 | 0.03 | -0.05 | -1.81 | 0.070 | [-0.10,  0.00] | [-0.10,  0.00] |
| Partisan extremism | 0.03 | 0.02 | 0.05 | 1.68 | 0.093 | [-0.01,  0.08] | [-0.01,  0.10] |
| N | | | | | | 1,506 | |
| R2 | | | | | | 0.191 | |
| Adjusted R2 | | | | | | 0.185 | |
| Residual SE | | | | | | 0.719  (df = 1494) | |
| F Statistic | | | | | | 32.15***  (df = 11; 1494) | |

**Supplementary Table 25: Full Regression Table for Step 5 - Defection from Rules of the Game**

|  | **b** | **SE** | **β** | **t** | **p** | **Unstandardized 95% CI** | **Standardized 95% CI** |
| --- | --- | --- | --- | --- | --- | --- | --- |
| Constant | 0.44 | 0.13 |  | 3.46 | 0.001 | [0.19,  0.69] | [-0.04,  0.04] |
| White | -0.19 | 0.08 | -0.11 | -2.53 | 0.012 | [-0.34,  -0.04] | [-0.20,  -0.03] |
| Black | 0.07 | 0.09 | 0.03 | 0.79 | 0.432 | [-0.11,  0.25] | [-0.04,  0.10] |
| Latino | 0.05 | 0.09 | 0.02 | 0.55 | 0.579 | [-0.12,  0.22] | [-0.05,  0.10] |
| Age | -0.00 | 0.00 | -0.06 | -2.71 | 0.007 | [-0.01,  -0.00] | [-0.11,  -0.02] |
| Sex (1=male, 2=female) | 0.01 | 0.03 | 0.01 | 0.32 | 0.746 | [-0.06,  0.08] | [-0.04,  0.05] |
| Income | -0.00 | 0.01 | -0.00 | -0.15 | 0.882 | [-0.02,  0.01] | [-0.05,  0.04] |
| Education | -0.08 | 0.02 | -0.11 | -4.47 | <0.001 | [-0.11,  -0.04] | [-0.15,  -0.06] |
| Conservatism | 0.00 | 0.02 | 0.00 | 0.01 | 0.989 | [-0.04,  0.04] | [-0.06,  0.06] |
| Republican ID | 0.02 | 0.01 | 0.05 | 1.58 | 0.115 | [-0.00,  0.04] | [-0.01,  0.11] |
| RWA | 0.31 | 0.02 | 0.37 | 12.51 | <0.001 | [0.26,  0.36] | [0.31,  0.43] |
| SDO | 0.21 | 0.02 | 0.22 | 8.93 | <0.001 | [0.17,  0.26] | [0.17,  0.27] |
| PSJ | -0.11 | 0.02 | -0.12 | -4.82 | <0.001 | [-0.15,  -0.06] | [-0.16,  -0.07] |
| N | | | | | | 1,462 | |
| R2 | | | | | | 0.362 | |
| Adjusted R2 | | | | | | 0.357 | |
| Residual SE | | | | | | 0.635  (df = 1449) | |
| F Statistic | | | | | | 68.62***  (df = 12; 1449) | |

**Supplementary Table 26: Full Regression Table for Step 6 - Defection from Rules of the Game**

|  | **b** | **SE** | **β** | **t** | **p** | **Unstandardized 95% CI** | **Standardized 95% CI** |
| --- | --- | --- | --- | --- | --- | --- | --- |
| Constant | 0.42 | 0.13 |  | 3.26 | 0.001 | [0.17,  0.67] | [-0.04,  0.04] |
| White | -0.20 | 0.08 | -0.12 | -2.62 | 0.009 | [-0.35,  -0.05] | [-0.21,  -0.03] |
| Black | 0.05 | 0.09 | 0.02 | 0.60 | 0.548 | [-0.12,  0.23] | [-0.05,  0.10] |
| Latino | 0.04 | 0.09 | 0.02 | 0.48 | 0.632 | [-0.13,  0.21] | [-0.06,  0.09] |
| Age | -0.00 | 0.00 | -0.07 | -2.92 | 0.004 | [-0.01,  -0.00] | [-0.12,  -0.02] |
| Sex (1=male, 2=female) | 0.00 | 0.03 | 0.00 | 0.10 | 0.923 | [-0.06,  0.07] | [-0.04,  0.04] |
| Income | -0.00 | 0.01 | -0.01 | -0.39 | 0.700 | [-0.02,  0.01] | [-0.05,  0.04] |
| Education | -0.08 | 0.02 | -0.11 | -4.44 | <0.001 | [-0.11,  -0.04] | [-0.15,  -0.06] |
| Conservatism | -0.00 | 0.02 | -0.00 | -0.03 | 0.979 | [-0.04,  0.04] | [-0.06,  0.06] |
| Republican ID | 0.02 | 0.01 | 0.06 | 1.79 | 0.074 | [-0.00,  0.05] | [-0.01,  0.12] |
| Ideological extremism | -0.02 | 0.02 | -0.02 | -0.67 | 0.506 | [-0.06,  0.03] | [-0.06,  0.03] |
| Partisan extremism | 0.04 | 0.02 | 0.05 | 1.91 | 0.056 | [0.00,  0.07] | [-0.00,  0.10] |
| RWA | 0.31 | 0.02 | 0.37 | 12.42 | <0.001 | [0.26,  0.35] | [0.31,  0.43] |
| SDO | 0.21 | 0.02 | 0.22 | 8.95 | <0.001 | [0.17,  0.26] | [0.17,  0.27] |
| PSJ | -0.11 | 0.02 | -0.12 | -5.03 | <0.001 | [-0.15,  -0.07] | [-0.17,  -0.07] |
| N | | | | | | 1,462 | |
| R2 | | | | | | 0.364 | |
| Adjusted R2 | | | | | | 0.358 | |
| Residual SE | | | | | | 0.634  (df = 1447) | |
| F Statistic | | | | | | 59.2***  (df = 14; 1447) | |

**Supplementary Table 27: Full Regression Table for Step 1 - Willingness to Use Violence**

|  | **b** | **SE** | **β** | **t** | **p** | **Unstandardized 95% CI** | **Standardized 95% CI** |
| --- | --- | --- | --- | --- | --- | --- | --- |
| Constant | 1.04 | 0.16 |  | 6.35 | <0.001 | [0.72,  1.36] | [-0.05,  0.05] |
| White | -0.09 | 0.09 | -0.05 | -0.97 | 0.334 | [-0.27,  0.09] | [-0.14,  0.05] |
| Black | 0.25 | 0.13 | 0.08 | 1.95 | 0.052 | [-0.00,  0.49] | [-0.00,  0.17] |
| Latino | 0.14 | 0.11 | 0.05 | 1.24 | 0.214 | [-0.08,  0.36] | [-0.03,  0.14] |
| Age | -0.01 | 0.00 | -0.16 | -6.07 | <0.001 | [-0.01,  -0.01] | [-0.21,  -0.11] |
| Sex (1=male, 2=female) | 0.00 | 0.05 | 0.00 | 0.03 | 0.972 | [-0.09,  0.09] | [-0.05,  0.05] |
| Income | -0.04 | 0.01 | -0.09 | -3.10 | 0.002 | [-0.06,  -0.01] | [-0.15,  -0.03] |
| Education | -0.12 | 0.02 | -0.13 | -5.11 | <0.001 | [-0.16,  -0.07] | [-0.18,  -0.08] |
| N | | | | | | 1,519 | |
| R2 | | | | | | 0.099 | |
| Adjusted R2 | | | | | | 0.095 | |
| Residual SE | | | | | | 0.896  (df = 1511) | |
| F Statistic | | | | | | 23.821***  (df = 7; 1511) | |

**Supplementary Table 28: Full Regression Table for Step 2 - Willingness to Use Violence**

|  | **b** | **SE** | **β** | **t** | **p** | **Unstandardized 95% CI** | **Standardized 95% CI** |
| --- | --- | --- | --- | --- | --- | --- | --- |
| Constant | -0.16 | 0.06 |  | -2.52 | 0.011 | [-0.29,  -0.04] | [-0.05,  0.05] |
| Conservatism | 0.06 | 0.03 | 0.07 | 2.05 | 0.040 | [0.00,  0.11] | [0.01,  0.14] |
| Republican ID | -0.01 | 0.02 | -0.02 | -0.45 | 0.655 | [-0.04,  0.02] | [-0.09,  0.05] |
| N | | | | | | 1,506 | |
| R2 | | | | | | 0.004 | |
| Adjusted R2 | | | | | | 0.003 | |
| Residual SE | | | | | | 0.929  (df = 1503) | |
| F Statistic | | | | | | 3.021**  (df = 2; 1503) | |

**Supplementary Table 29: Full Regression Table for Step 3 - Willingness to Use Violence**

|  | **b** | **SE** | **β** | **t** | **p** | **Unstandardized 95% CI** | **Standardized 95% CI** |
| --- | --- | --- | --- | --- | --- | --- | --- |
| Constant | 0.78 | 0.18 |  | 4.42 | <0.001 | [0.43,  1.12] | [-0.05,  0.05] |
| White | -0.11 | 0.09 | -0.06 | -1.20 | 0.232 | [-0.30,  0.07] | [-0.15,  0.04] |
| Black | 0.26 | 0.13 | 0.09 | 2.01 | 0.044 | [0.01,  0.51] | [0.00,  0.18] |
| Latino | 0.13 | 0.11 | 0.05 | 1.18 | 0.237 | [-0.09,  0.36] | [-0.03,  0.14] |
| Age | -0.01 | 0.00 | -0.17 | -6.42 | <0.001 | [-0.01,  -0.01] | [-0.22,  -0.12] |
| Sex (1=male, 2=female) | 0.01 | 0.05 | 0.01 | 0.27 | 0.784 | [-0.08,  0.10] | [-0.04,  0.06] |
| Income | -0.04 | 0.01 | -0.09 | -3.02 | 0.003 | [-0.06,  -0.01] | [-0.15,  -0.03] |
| Education | -0.10 | 0.02 | -0.11 | -4.21 | <0.001 | [-0.14,  -0.05] | [-0.16,  -0.06] |
| Conservatism | 0.05 | 0.03 | 0.06 | 1.70 | 0.089 | [-0.01,  0.10] | [-0.01,  0.13] |
| Republican ID | 0.02 | 0.02 | 0.04 | 1.07 | 0.285 | [-0.01,  0.05] | [-0.03,  0.11] |
| N | | | | | | 1,496 | |
| R2 | | | | | | 0.102 | |
| Adjusted R2 | | | | | | 0.097 | |
| Residual SE | | | | | | 0.887  (df = 1486) | |
| F Statistic | | | | | | 18.76***  (df = 9; 1486) | |

**Supplementary Table 30: Full Regression Table for Step 4 - Willingness to Use Violence**

|  | **b** | **SE** | **β** | **t** | **p** | **Unstandardized 95% CI** | **Standardized 95% CI** |
| --- | --- | --- | --- | --- | --- | --- | --- |
| Constant | 0.79 | 0.18 |  | 4.34 | <0.001 | [0.43,  1.14] | [-0.05,  0.05] |
| White | -0.11 | 0.09 | -0.06 | -1.17 | 0.243 | [-0.29,  0.07] | [-0.15,  0.04] |
| Black | 0.26 | 0.13 | 0.09 | 2.03 | 0.042 | [0.01,  0.51] | [0.00,  0.18] |
| Latino | 0.14 | 0.11 | 0.05 | 1.19 | 0.233 | [-0.09,  0.36] | [-0.03,  0.14] |
| Age | -0.01 | 0.00 | -0.17 | -6.37 | <0.001 | [-0.01,  -0.01] | [-0.22,  -0.12] |
| Sex (1=male, 2=female) | 0.01 | 0.05 | 0.01 | 0.27 | 0.791 | [-0.08,  0.10] | [-0.04,  0.06] |
| Income | -0.04 | 0.01 | -0.09 | -2.99 | 0.003 | [-0.06,  -0.01] | [-0.15,  -0.03] |
| Education | -0.10 | 0.02 | -0.11 | -4.21 | <0.001 | [-0.14,  -0.05] | [-0.16,  -0.06] |
| Conservatism | 0.05 | 0.03 | 0.06 | 1.67 | 0.094 | [-0.01,  0.10] | [-0.01,  0.13] |
| Republican ID | 0.02 | 0.02 | 0.04 | 1.08 | 0.280 | [-0.01,  0.05] | [-0.03,  0.11] |
| Ideological extremism | -0.01 | 0.03 | -0.01 | -0.34 | 0.737 | [-0.07,  0.05] | [-0.07,  0.05] |
| Partisan extremism | -0.00 | 0.02 | -0.00 | -0.05 | 0.958 | [-0.05,  0.05] | [-0.06,  0.06] |
| N | | | | | | 1,496 | |
| R2 | | | | | | 0.102 | |
| Adjusted R2 | | | | | | 0.095 | |
| Residual SE | | | | | | 0.887  (df = 1484) | |
| F Statistic | | | | | | 15.35***  (df = 11; 1484) | |

**Supplementary Table 31: Full Regression Table for Step 5 - Willingness to Use Violence**

|  | **b** | **SE** | **β** | **t** | **p** | **Unstandardized 95% CI** | **Standardized 95% CI** |
| --- | --- | --- | --- | --- | --- | --- | --- |
| Constant | 0.88 | 0.17 |  | 5.04 | <0.001 | [0.54,  1.22] | [-0.05,  0.05] |
| White | -0.07 | 0.09 | -0.04 | -0.82 | 0.414 | [-0.25,  0.10] | [-0.13,  0.05] |
| Black | 0.21 | 0.12 | 0.07 | 1.72 | 0.086 | [-0.03,  0.45] | [-0.01,  0.16] |
| Latino | 0.13 | 0.11 | 0.05 | 1.23 | 0.218 | [-0.08,  0.34] | [-0.03,  0.13] |
| Age | -0.01 | 0.00 | -0.15 | -5.59 | <0.001 | [-0.01,  -0.01] | [-0.20,  -0.10] |
| Sex (1=male, 2=female) | 0.01 | 0.04 | 0.00 | 0.15 | 0.882 | [-0.08,  0.09] | [-0.04,  0.05] |
| Income | -0.03 | 0.01 | -0.08 | -2.71 | 0.007 | [-0.05,  -0.01] | [-0.13,  -0.02] |
| Education | -0.06 | 0.02 | -0.07 | -2.75 | 0.006 | [-0.11,  -0.02] | [-0.13,  -0.02] |
| Conservatism | -0.02 | 0.03 | -0.03 | -0.72 | 0.473 | [-0.08,  0.04] | [-0.10,  0.05] |
| Republican ID | -0.01 | 0.02 | -0.02 | -0.49 | 0.621 | [-0.04,  0.02] | [-0.09,  0.05] |
| RWA | 0.03 | 0.03 | 0.03 | 1.05 | 0.292 | [-0.03,  0.09] | [-0.03,  0.09] |
| SDO | 0.36 | 0.03 | 0.32 | 11.14 | <0.001 | [0.29,  0.42] | [0.26,  0.37] |
| PSJ | 0.03 | 0.02 | 0.02 | 1.10 | 0.271 | [-0.02,  0.08] | [-0.02,  0.07] |
| N | | | | | | 1,457 | |
| R2 | | | | | | 0.193 | |
| Adjusted R2 | | | | | | 0.186 | |
| Residual SE | | | | | | 0.837  (df = 1444) | |
| F Statistic | | | | | | 28.738***  (df = 12; 1444) | |

**Supplementary Table 32: Full Regression Table for Step 6 - Willingness to Use Violence**

|  | **b** | **SE** | **β** | **t** | **p** | **Unstandardized 95% CI** | **Standardized 95% CI** |
| --- | --- | --- | --- | --- | --- | --- | --- |
| Constant | 0.87 | 0.18 |  | 4.87 | <0.001 | [0.52,  1.22] | [-0.05,  0.05] |
| White | -0.07 | 0.09 | -0.04 | -0.84 | 0.401 | [-0.25,  0.10] | [-0.13,  0.05] |
| Black | 0.21 | 0.12 | 0.07 | 1.72 | 0.085 | [-0.03,  0.45] | [-0.01,  0.16] |
| Latino | 0.13 | 0.11 | 0.05 | 1.22 | 0.224 | [-0.08,  0.34] | [-0.03,  0.13] |
| Age | -0.01 | 0.00 | -0.15 | -5.61 | <0.001 | [-0.01,  -0.01] | [-0.20,  -0.10] |
| Sex (1=male, 2=female) | 0.01 | 0.05 | 0.00 | 0.18 | 0.857 | [-0.08,  0.10] | [-0.04,  0.05] |
| Income | -0.03 | 0.01 | -0.07 | -2.65 | 0.008 | [-0.05,  -0.01] | [-0.13,  -0.02] |
| Education | -0.06 | 0.02 | -0.07 | -2.78 | 0.005 | [-0.11,  -0.02] | [-0.13,  -0.02] |
| Conservatism | -0.02 | 0.03 | -0.03 | -0.69 | 0.490 | [-0.08,  0.04] | [-0.10,  0.05] |
| Republican ID | -0.01 | 0.02 | -0.02 | -0.56 | 0.577 | [-0.04,  0.02] | [-0.09,  0.05] |
| Ideological extremism | 0.02 | 0.03 | 0.02 | 0.68 | 0.498 | [-0.04,  0.08] | [-0.04,  0.07] |
| Partisan extremism | -0.00 | 0.03 | -0.00 | -0.11 | 0.910 | [-0.05,  0.05] | [-0.06,  0.05] |
| RWA | 0.03 | 0.03 | 0.03 | 1.08 | 0.279 | [-0.03,  0.09] | [-0.03,  0.09] |
| SDO | 0.36 | 0.03 | 0.32 | 11.13 | <0.001 | [0.39,  0.42] | [0.26,  0.37] |
| PSJ | 0.03 | 0.03 | 0.03 | 1.12 | 0.263 | [-0.02,  0.08] | [-0.02,  0.07] |
| N | | | | | | 1,457 | |
| R2 | | | | | | 0.193 | |
| Adjusted R2 | | | | | | 0.185 | |
| Residual SE | | | | | | 0.837  (df = 1444) | |
| F Statistic | | | | | | 28.738***  (df = 12; 1444) | |

**Supplementary Table 33: Full Regression Table for Step 1 - Tolerance of Disliked Group**

|  | **b** | **SE** | **β** | **t** | **p** | **Unstandardized 95% CI** | **Standardized 95% CI** |
| --- | --- | --- | --- | --- | --- | --- | --- |
| Constant | -0.10 | 0.16 |  | -0.59 | 0.557 | [-0.42,  0.23] | [-0.05,  0.05] |
| White | 0.15 | 0.11 | 0.08 | 1.43 | 0.154 | [-0.06,  0.37] | [-0.03,  0.18] |
| Black | -0.07 | 0.12 | -0.02 | -0.58 | 0.565 | [-0.32,  0.17] | [-0.11,  0.06] |
| Latino | 0.06 | 0.12 | 0.02 | 0.48 | 0.631 | [-0.18,  0.30] | [-0.07,  0.11] |
| Age | 0.00 | 0.00 | 0.02 | 0.64 | 0.525 | [-0.00,  0.00] | [-0.04,  0.07] |
| Sex (1=male, 2=female) | -0.20 | 0.05 | -0.10 | -3.88 | <0.001 | [-0.29,  -0.10] | [-0.16,  -0.05] |
| Income | 0.00 | 0.01 | 0.00 | 0.06 | 0.953 | [-0.02,  0.03] | [-0.06,  0.06] |
| Education | 0.07 | 0.03 | 0.08 | 2.73 | 0.006 | [0.02,  0.12] | [0.02,  0.14] |
| N | | | | | | 1,407 | |
| R2 | | | | | | 0.028 | |
| Adjusted R2 | | | | | | 0.023 | |
| Residual SE | | | | | | 0.930  (df = 1399) | |
| F Statistic | | | | | | 5.768***  (df = 7; 1399) | |

**Supplementary Table 34: Full Regression Table for Step 2 - Tolerance of Disliked Group**

|  | **b** | **SE** | **β** | **t** | **p** | **Unstandardized 95% CI** | **Standardized 95% CI** |
| --- | --- | --- | --- | --- | --- | --- | --- |
| Constant | 0.07 | 0.08 |  | 0.92 | 0.360 | [-0.08,  0.22] | [-0.05,  0.05] |
| Conservatism | -0.01 | 0.03 | -0.01 | -0.22 | 0.822 | [-0.06,  0.05] | [-0.08,  0.06] |
| Republican ID | -0.01 | 0.01 | -0.03 | -0.90 | 0.366 | [-0.04,  0.02] | [-0.10,  0.04] |
| N | | | | | | 1,402 | |
| R2 | | | | | | 0.001 | |
| Adjusted R2 | | | | | | 0.000 | |
| Residual SE | | | | | | 0.941  (df = 1399) | |
| F Statistic | | | | | | 0.912  (df = 2; 1399) | |

**Supplementary Table 35: Full Regression Table for Step 3 - Tolerance of Disliked Group**

|  | **b** | **SE** | **β** | **t** | **p** | **Unstandardized 95% CI** | **Standardized 95% CI** |
| --- | --- | --- | --- | --- | --- | --- | --- |
| Constant | -0.02 | 0.18 |  | -0.12 | 0.903 | [-0.38,  0.34] | [-0.05,  0.05] |
| White | 0.20 | 0.11 | 0.10 | 1.84 | 0.066 | [-0.01,  0.42] | [-0.01,  0.21] |
| Black | -0.08 | 0.13 | -0.03 | -0.60 | 0.552 | [-0.33,  0.18] | [-0.11,  0.06] |
| Latino | 0.08 | 0.13 | 0.03 | 0.64 | 0.523 | [-0.17,  0.33] | [-0.06,  0.12] |
| Age | 0.00 | 0.00 | 0.02 | 0.73 | 0.468 | [-0.00,  0.00] | [-0.03,  0.07] |
| Sex (1=male, 2=female) | -0.21 | 0.05 | -0.11 | -4.12 | <0.001 | [-0.31,  -0.11] | [-0.16,  -0.06] |
| Income | 0.00 | 0.01 | 0.01 | 0.17 | 0.866 | [-0.02,  0.03] | [-0.05,  0.06] |
| Education | 0.06 | 0.03 | 0.07 | 2.34 | 0.019 | [0.01,  0.12] | [0.01,  0.13] |
| Conservatism | 0.01 | 0.03 | 0.02 | 0.45 | 0.653 | [-0.04,  0.07] | [-0.05,  0.09] |
| Republican ID | -0.03 | 0.02 | -0.07 | -2.04 | 0.041 | [-0.06,  -0.00] | [-0.14,  -0.00] |
| N | | | | | | 1,393 | |
| R2 | | | | | | 0.033 | |
| Adjusted R2 | | | | | | 0.027 | |
| Residual SE | | | | | | 0.930  (df = 1383) | |
| F Statistic | | | | | | 5.23***  (df = 9; 1383) | |

**Supplementary Table 36: Full Regression Table for Step 4 - Tolerance of Disliked Group**

|  | **b** | **SE** | **β** | **t** | **p** | **Unstandardized 95% CI** | **Standardized 95% CI** |
| --- | --- | --- | --- | --- | --- | --- | --- |
| Constant | -0.03 | 0.19 |  | -0.14 | 0.889 | [-0.39,  0.34] | [-0.05,  0.05] |
| White | 0.21 | 0.11 | 0.10 | 1.87 | 0.062 | [-0.01,  0.43] | [-0.01,  0.21] |
| Black | -0.06 | 0.13 | -0.02 | -0.45 | 0.651 | [-0.32,  0.20] | [-0.11,  0.07] |
| Latino | 0.08 | 0.13 | 0.03 | 0.67 | 0.503 | [-0.16,  0.33] | [-0.06,  0.12] |
| Age | 0.00 | 0.00 | 0.03 | 0.94 | 0.347 | [-0.00,  0.00] | [-0.03,  0.08] |
| Sex (1=male, 2=female) | -0.20 | 0.05 | -0.11 | -3.89 | <0.001 | [-0.30,  -0.10] | [-0.16,  -0.05] |
| Income | 0.01 | 0.01 | 0.01 | 0.41 | 0.685 | [-0.02,  0.03] | [-0.05,  0.07] |
| Education | 0.06 | 0.03 | 0.07 | 2.29 | 0.022 | [0.01,  0.12] | [0.01,  0.13] |
| Conservatism | 0.02 | 0.03 | 0.02 | 0.61 | 0.541 | [-0.04,  0.08] | [-0.05,  0.09] |
| Republican ID | -0.04 | 0.02 | -0.08 | -2.36 | 0.018 | [-0.07,  -0.01] | [-0.15,  -0.01] |
| Ideological extremism | 0.05 | 0.03 | 0.05 | 1.54 | 0.125 | [-0.01,  0.12] | [-0.01,  0.10] |
| Partisan extremism | -0.05 | 0.03 | -0.05 | -1.74 | 0.082 | [-0.10,  0.01] | [-0.11,  0.01] |
| N | | | | | | 1,393 | |
| R2 | | | | | | 0.036 | |
| Adjusted R2 | | | | | | 0.028 | |
| Residual SE | | | | | | 0.929  (df = 1381) | |
| F Statistic | | | | | | 4.65***  (df = 11; 1381) | |

**Supplementary Table 37: Full Regression Table for Step 5 - Tolerance of Disliked Group**

|  | **b** | **SE** | **β** | **t** | **p** | **Unstandardized 95% CI** | **Standardized 95% CI** |
| --- | --- | --- | --- | --- | --- | --- | --- |
| Constant | -0.02 | 0.19 |  | -0.12 | 0.903 | [-0.39,  0.34] | [-0.05,  0.05] |
| White | 0.20 | 0.11 | 0.10 | 1.84 | 0.066 | [-0.01,  0.42] | [-0.01,  0.21] |
| Black | -0.10 | 0.13 | -0.03 | -0.76 | 0.447 | [-0.35,  0.16] | [-0.12,  0.05] |
| Latino | 0.10 | 0.13 | 0.04 | 0.82 | 0.411 | [-0.14,  0.35] | [-0.05,  0.13] |
| Age | 0.00 | 0.00 | 0.01 | 0.22 | 0.825 | [-0.00,  0.00] | [-0.05,  0.06] |
| Sex (1=male, 2=female) | -0.18 | 0.05 | -0.10 | -3.62 | <0.001 | [-0.28,  -0.08] | [-0.15,  -0.04] |
| Income | -0.00 | 0.01 | -0.01 | -0.31 | 0.758 | [-0.03,  0.02] | [-0.07,  0.05] |
| Education | 0.03 | 0.03 | 0.03 | 0.99 | 0.320 | [-0.03,  0.08] | [-0.03,  0.09] |
| Conservatism | 0.04 | 0.03 | 0.05 | 1.46 | 0.144 | [-0.02,  0.10] | [-0.02,  0.13] |
| Republican ID | -0.01 | 0.02 | -0.03 | -0.87 | 0.386 | [-0.05,  0.02] | [-0.11,  0.04] |
| RWA | -0.18 | 0.04 | -0.18 | -5.15 | <0.001 | [-0.25,  -0.11] | [-0.25,  -0.11] |
| SDO | 0.04 | 0.03 | 0.04 | 1.18 | 0.238 | [-0.03,  0.11] | [-0.02,  0.10] |
| PSJ | 0.13 | 0.03 | 0.12 | 4.02 | <0.001 | [0.07,  0.20] | [0.06,  0.18] |
| N | | | | | | 1,363 | |
| R2 | | | | | | 0.071 | |
| Adjusted R2 | | | | | | 0.063 | |
| Residual SE | | | | | | 0.912  (df = 1350) | |
| F Statistic | | | | | | 8.580***  (df = 12; 1350) | |

**Supplementary Table 38: Full Regression Table for Step 6 - Tolerance of Disliked Group**

|  | **b** | **SE** | **β** | **t** | **p** | **Unstandardized 95% CI** | **Standardized 95% CI** |
| --- | --- | --- | --- | --- | --- | --- | --- |
| Constant | -0.01 | 0.19 |  | -0.04 | 0.968 | [-0.38,  0.36] | [-0.05,  0.05] |
| White | 0.21 | 0.11 | 0.10 | 1.91 | 0.056 | [-0.01,  0.43] | [-0.00,  0.21] |
| Black | -0.07 | 0.13 | -0.03 | -0.56 | 0.574 | [-0.33,  0.18] | [-0.11,  0.06] |
| Latino | 0.11 | 0.13 | 0.04 | 0.86 | 0.389 | [-0.14,  0.36] | [-0.05,  0.13] |
| Age | 0.00 | 0.00 | 0.01 | 0.41 | 0.683 | [-0.00,  0.00] | [-0.05,  0.07] |
| Sex (1=male, 2=female) | -0.17 | 0.05 | -0.09 | -3.31 | 0.001 | [-0.27,  -0.07] | [-0.14,  -0.04] |
| Income | -0.00 | 0.01 | -0.00 | -0.05 | 0.958 | [-0.03,  0.02] | [-0.06,  0.06] |
| Education | 0.03 | 0.03 | 0.03 | 0.97 | 0.334 | [-0.03,  0.08] | [-0.03,  0.09] |
| Conservatism | 0.05 | 0.03 | 0.06 | 1.55 | 0.121 | [-0.01,  0.11] | [-0.02,  0.13] |
| Republican ID | -0.02 | 0.02 | -0.05 | -1.20 | 0.230 | [-0.05,  0.01] | [-0.12,  0.03] |
| Ideological extremism | 0.05 | 0.03 | 0.04 | 1.46 | 0.146 | [-0.02,  0.11] | [-0.01,  0.10] |
| Partisan extremism | -0.05 | 0.03 | -0.06 | -2.00 | 0.046 | [-0.11,  -0.00] | [-0.12,  -0.00] |
| RWA | -0.18 | 0.04 | -0.18 | -5.00 | <0.001 | [-0.25,  -0.11] | [-0.25,  -0.11] |
| SDO | 0.04 | 0.04 | 0.04 | 1.17 | 0.242 | [-0.03,  0.11] | [-0.02,  0.10] |
| PSJ | 0.14 | 0.03 | 0.13 | 4.24 | <0.001 | [0.08,  0.21] | [0.07,  0.19] |
| N | | | | | | 1,363 | |
| R2 | | | | | | 0.074 | |
| Adjusted R2 | | | | | | 0.064 | |
| Residual SE | | | | | | 0.911  (df = 1348) | |
| F Statistic | | | | | | 7.70***  (df = 14; 1348) | |

**Supplementary Table 39: Full Regression Table for Step 1 - Willingness to Vote for Anti-democratic Candidate**

|  | **b** | **SE** | **β** | **t** | **p** | **Unstandardized 95% CI** | **Standardized 95% CI** |
| --- | --- | --- | --- | --- | --- | --- | --- |
| Constant | 0.80 | 0.14 |  | 5.54 | <0.001 | [0.52,  1.08] | [-0.05,  0.05] |
| White | 0.11 | 0.08 | 0.06 | 1.29 | 0.198 | [-0.06,  0.28] | [-0.03,  0.14] |
| Black | 0.08 | 0.10 | 0.03 | 0.74 | 0.458 | [-0.13,  0.28] | [-0.05,  0.10] |
| Latino | 0.19 | 0.10 | 0.07 | 1.85 | 0.064 | [-0.01,  0.39] | [-0.00,  0.15] |
| Age | -0.00 | 0.00 | -0.07 | -2.84 | 0.005 | [-0.01,  -0.00] | [-0.12,  -0.02] |
| Sex (1=male, 2=female) | 0.03 | 0.05 | 0.02 | 0.71 | 0.479 | [-0.06,  0.12] | [-0.03,  0.07] |
| Income | -0.01 | 0.01 | -0.03 | -1.10 | 0.273 | [-0.03,  0.01] | [-0.09,  0.02] |
| Education | -0.21 | 0.02 | -0.25 | -9.22 | <0.001 | [-0.26,  -0.17] | [-0.30,  -0.19] |
| N | | | | | | 1,502 | |
| R2 | | | | | | 0.082 | |
| Adjusted R2 | | | | | | 0.077 | |
| Residual SE | | | | | | 0.882  (df = 1494) | |
| F Statistic | | | | | | 18.969***  (df = 7; 1494) | |

**Supplementary Table 40: Full Regression Table for Step 2 - Willingness to Vote for Anti-democratic Candidate**

|  | **b** | **SE** | **β** | **t** | **p** | **Unstandardized 95% CI** | **Standardized 95% CI** |
| --- | --- | --- | --- | --- | --- | --- | --- |
| Constant | -0.99 | 0.05 |  | -18.54 | <0.001 | [-1.09,  -0.88] | [-0.05,  0.05] |
| Conservatism | 0.16 | 0.02 | 0.20 | 6.43 | <0.001 | [0.11,  0.21] | [0.14,  0.26] |
| Republican ID | 0.13 | 0.01 | 0.31 | 9.70 | <0.001 | [0.11,  0.16] | [0.25,  0.37] |
| N | | | | | | 1,489 | |
| R2 | | | | | | 0.217 | |
| Adjusted R2 | | | | | | 0.216 | |
| Residual SE | | | | | | 0.811  (df = 1486) | |
| F Statistic | | | | | | 206.074***  (df = 2; 1486) | |

**Supplementary Table 41: Full Regression Table for Step 3 - Willingness to Vote for Anti-democratic Candidate**

|  | **b** | **SE** | **β** | **t** | **p** | **Unstandardized 95% CI** | **Standardized 95% CI** |
| --- | --- | --- | --- | --- | --- | --- | --- |
| Constant | -0.35 | 0.14 |  | -2.39 | 0.017 | [-0.63,  -0.06] | [-0.04,  0.04] |
| White | -0.00 | 0.08 | -0.00 | -0.01 | 0.990 | [-0.16,  0.16] | [-0.08,  0.08] |
| Black | 0.29 | 0.10 | 0.10 | 2.80 | 0.005 | [0.09,  0.50] | [0.03,  0.18] |
| Latino | 0.21 | 0.10 | 0.08 | 2.19 | 0.028 | [0.02,  0.41] | [0.01,  0.16] |
| Age | -0.01 | 0.00 | -0.12 | -5.45 | <0.001 | [-0.01,  -0.00] | [-0.17,  -0.08] |
| Sex (1=male, 2=female) | 0.09 | 0.04 | 0.05 | 2.16 | 0.031 | [0.01,  0.17] | [0.00,  0.09] |
| Income | -0.02 | 0.01 | -0.04 | -1.79 | 0.073 | [-0.04,  0.00] | [-0.09,  0.00] |
| Education | -0.13 | 0.02 | -0.15 | -6.28 | <0.001 | [-0.17,  -0.09] | [-0.20,  -0.10] |
| Conservatism | 0.15 | 0.02 | 0.18 | 6.00 | <0.001 | [0.10,  0.19] | [0.12,  0.24] |
| Republican ID | 0.15 | 0.01 | 0.35 | 11.18 | <0.001 | [0.12,  0.18] | [0.29,  0.42] |
| N | | | | | | 1,479 | |
| R2 | | | | | | 0.298 | |
| Adjusted R2 | | | | | | 0.293 | |
| Residual SE | | | | | | 0.770  (df = 1469) | |
| F Statistic | | | | | | 69.17***  (df = 9; 1469) | |

**Supplementary Table 42: Full Regression Table for Step 4 - Willingness to Vote for Anti-democratic Candidate**

|  | **b** | **SE** | **β** | **t** | **p** | **Unstandardized 95% CI** | **Standardized 95% CI** |
| --- | --- | --- | --- | --- | --- | --- | --- |
| Constant | -0.41 | 0.15 |  | -2.75 | 0.006 | [-0.69,  -0.12] | [-0.04,  0.04] |
| White | -0.02 | 0.08 | -0.01 | -0.27 | 0.789 | [-0.19,  0.14] | [-0.10,  0.07] |
| Black | 0.28 | 0.11 | 0.10 | 2.64 | 0.008 | [0.07,  0.48] | [0.03,  0.17] |
| Latino | 0.20 | 0.10 | 0.08 | 2.05 | 0.041 | [0.01,  0.39] | [0.01,  0.15] |
| Age | -0.01 | 0.00 | -0.13 | -5.80 | <0.001 | [-0.01,  -0.00] | [-0.17,  -0.09] |
| Sex (1=male, 2=female) | 0.09 | 0.04 | 0.05 | 2.12 | 0.034 | [0.01,  0.17] | [0.00,  0.09] |
| Income | -0.02 | 0.01 | -0.05 | -1.89 | 0.059 | [-0.04,  0.00] | [-0.10,  0.00] |
| Education | -0.13 | 0.02 | -0.15 | -6.38 | <0.001 | [-0.17,  -0.09] | [-0.20,  -0.11] |
| Conservatism | 0.15 | 0.02 | 0.19 | 6.04 | <0.001 | [0.10,  0.20] | [0.13,  0.25] |
| Republican ID | 0.15 | 0.01 | 0.35 | 10.84 | <0.001 | [0.12,  0.18] | [0.29,  0.42] |
| Ideological extremism | 0.06 | 0.03 | 0.06 | 2.32 | 0.021 | [0.01,  0.12] | [0.01,  0.10] |
| Partisan extremism | 0.03 | 0.02 | 0.03 | 1.22 | 0.222 | [-0.02,  0.07] | [-0.02,  0.08] |
| N | | | | | | 1,479 | |
| R2 | | | | | | 0.303 | |
| Adjusted R2 | | | | | | 0.298 | |
| Residual SE | | | | | | 0.767  (df = 1467) | |
| F Statistic | | | | | | 57.97***  (df = 11; 1467) | |

**Supplementary Table 43: Full Regression Table for Step 5 - Willingness to Vote for Anti-democratic Candidate**

|  | **b** | **SE** | **β** | **t** | **p** | **Unstandardized 95% CI** | **Standardized 95% CI** |
| --- | --- | --- | --- | --- | --- | --- | --- |
| Constant | -0.20 | 0.14 |  | -1.39 | 0.163 | [-0.48,  0.08] | [-0.04,  0.04] |
| White | 0.03 | 0.08 | 0.01 | 0.35 | 0.729 | [-0.13,  0.19] | [-0.07,  0.10] |
| Black | 0.28 | 0.10 | 0.10 | 2.64 | 0.008 | [0.07,  0.48] | [0.02,  0.17] |
| Latino | 0.19 | 0.10 | 0.07 | 1.99 | 0.047 | [0.00,  0.38] | [0.00,  0.15] |
| Age | -0.01 | 0.00 | -0.11 | -4.73 | <0.001 | [-0.01,  -0.00] | [-0.15,  -0.06] |
| Sex (1=male, 2=female) | 0.06 | 0.04 | 0.04 | 1.67 | 0.096 | [-0.01,  0.14] | [-0.01,  0.08] |
| Income | -0.01 | 0.01 | -0.03 | -1.39 | 0.163 | [-0.03,  0.01] | [-0.08,  0.01] |
| Education | -0.08 | 0.02 | -0.10 | -4.08 | <0.001 | [-0.12,  -0.04] | [-0.14,  -0.05] |
| Conservatism | 0.07 | 0.03 | 0.09 | 2.92 | 0.004 | [0.02,  0.12] | [0.03,  0.15] |
| Republican ID | 0.12 | 0.01 | 0.27 | 8.62 | <0.001 | [0.09,  0.14] | [0.21,  0.34] |
| RWA | 0.14 | 0.03 | 0.15 | 5.17 | <0.001 | [0.09,  0.19] | [0.09,  0.20] |
| SDO | 0.26 | 0.03 | 0.24 | 9.26 | <0.001 | [0.21,  0.32] | [0.19,  0.29] |
| PSJ | -0.02 | 0.02 | -0.02 | -0.71 | 0.475 | [-0.07,  0.03] | [-0.06,  0.03] |
| N | | | | | | 1,439 | |
| R2 | | | | | | 0.372 | |
| Adjusted R2 | | | | | | 0.366 | |
| Residual SE | | | | | | 0.725  (df = 1426) | |
| F Statistic | | | | | | 70.271***  (df = 12; 1426) | |

**Supplementary Table 44: Full Regression Table for Step 6 - Willingness to Vote for Anti-democratic Candidate**

|  | **b** | **SE** | **β** | **t** | **p** | **Unstandardized 95% CI** | **Standardized 95% CI** |
| --- | --- | --- | --- | --- | --- | --- | --- |
| Constant | -0.28 | 0.14 |  | -1.93 | 0.053 | [-0.56,  -0.00] | [-0.04,  0.04] |
| White | 0.01 | 0.08 | 0.00 | 0.06 | 0.951 | [-0.16,  0.17] | [-0.08,  0.09] |
| Black | 0.26 | 0.11 | 0.09 | 2.47 | 0.014 | [0.05,  0.47] | [0.02,  0.16] |
| Latino | 0.18 | 0.10 | 0.07 | 1.82 | 0.068 | [-0.01,  0.37] | [-0.01,  0.14] |
| Age | -0.01 | 0.00 | -0.12 | -5.10 | <0.001 | [-0.01,  -0.00] | [-0.16,  -0.07] |
| Sex (1=male, 2=female) | 0.07 | 0.04 | 0.04 | 1.67 | 0.094 | [-0.01,  0.14] | [-0.01,  0.08] |
| Income | -0.01 | 0.01 | -0.03 | -1.42 | 0.154 | [-0.03,  0.01] | [-0.08,  0.01] |
| Education | -0.09 | 0.02 | -0.10 | -4.22 | <0.001 | [-0.13,  -0.05] | [-0.15,  -0.05] |
| Conservatism | 0.08 | 0.03 | 0.10 | 3.06 | 0.002 | [0.03,  0.13] | [0.04,  0.16] |
| Republican ID | 0.11 | 0.01 | 0.27 | 8.19 | <0.001 | [0.09,  0.14] | [0.20,  0.33] |
| Ideological extremism | 0.09 | 0.03 | 0.08 | 3.49 | <0.001 | [0.04,  0.14] | [0.04,  0.13] |
| Partisan extremism | 0.03 | 0.02 | 0.03 | 1.28 | 0.199 | [-0.01,  0.07] | [-0.02,  0.08] |
| RWA | 0.14 | 0.03 | 0.15 | 5.24 | <0.001 | [0.09,  0.20] | [0.09,  0.20] |
| SDO | 0.27 | 0.03 | 0.24 | 9.60 | <0.001 | [0.21,  0.32] | [0.19,  0.29] |
| PSJ | -0.02 | 0.02 | -0.02 | -0.76 | 0.449 | [-0.06,  0.03] | [-0.06,  0.03] |
| N | | | | | | 1,439 | |
| R2 | | | | | | 0.381 | |
| Adjusted R2 | | | | | | 0.375 | |
| Residual SE | | | | | | 0.720  (df = 1424) | |
| F Statistic | | | | | | 69.59***  (df = 14; 1424) | |

**Supplementary Table 45: Full Regression Table for Step 1 - Approval of the January 6 insurrectionists**

|  | **b** | **SE** | **β** | **t** | **p** | **Unstandardized 95% CI** | **Standardized 95% CI** |
| --- | --- | --- | --- | --- | --- | --- | --- |
| Constant | 42.36 | 0.46 | 3.94 | 8.11 | 0.000 | [17.42,  106.71] | [3.45,  4.52] |
| White | 1.17 | 0.26 | 1.08 | 0.61 | 0.545 | [0.69,  1.94] | [0.84,  1.37] |
| Black | 0.88 | 0.32 | 0.96 | -0.41 | 0.680 | [0.47,  1.62] | [0.78,  1.17] |
| Latino | 1.12 | 0.31 | 1.04 | 0.36 | 0.716 | [0.60,  2.06] | [0.83,  1.29] |
| Age | 0.98 | 0.00 | 0.66 | -5.93 | 0.000 | [0.97,  0.98] | [0.57,  0.76] |
| Sex (1=male, 2=female) | 1.06 | 0.13 | 1.03 | 0.41 | 0.684 | [0.81,  1.37] | [0.90,  1.17] |
| Income | 0.93 | 0.03 | 0.84 | -2.30 | 0.021 | [0.87,  0.99] | [0.72,  0.97] |
| Education | 0.76 | 0.07 | 0.74 | -4.02 | 0.000 | [0.66,  0.87] | [0.64,  0.86] |
| N | | | | | | 1,454 | |
| Pseudo R2 | | | | | | 0.08 | |
| AIC | | | | | | 1464.40 | |
| BIC | | | | | | 1506.66 | |

**Supplementary Table 46: Full Regression Table for Step 2 - Approval of the January 6 insurrectionists**

|  | **b** | **SE** | **β** | **t** | **p** | **Unstandardized 95% CI** | **Standardized 95% CI** |
| --- | --- | --- | --- | --- | --- | --- | --- |
| Constant | 1.01 | 0.18 | 3.96 | 0.04 | 0.969 | [0.71,  1.42] | [3.46,  4.56] |
| Conservatism | 1.14 | 0.07 | 1.17 | 1.82 | 0.068 | [0.99,  1.32] | [0.99,  1.37] |
| Republican ID | 1.29 | 0.04 | 1.72 | 6.17 | 0.000 | [1.19,  1.40] | [1.45,  2.05] |
| N | | | | | | 1,440 | |
| Pseudo R2 | | | | | | 0.10 | |
| AIC | | | | | | 1433.12 | |
| BIC | | | | | | 1448.96 | |

**Supplementary Table 47: Full Regression Table for Step 3 - Approval of the January 6 insurrectionists**

|  | **b** | **SE** | **β** | **t** | **p** | **Unstandardized 95% CI** | **Standardized 95% CI** |
| --- | --- | --- | --- | --- | --- | --- | --- |
| Constant | 9.93 | 0.51 | 4.36 | 4.48 | 0.000 | [3.68,  27.51] | [3.77,  5.07] |
| White | 1.01 | 0.28 | 1.00 | 0.03 | 0.978 | [0.57,  1.71] | [0.77,  1.29] |
| Black | 1.26 | 0.33 | 1.08 | 0.70 | 0.482 | [0.65,  2.41] | [0.87,  1.33] |
| Latino | 1.19 | 0.33 | 1.06 | 0.54 | 0.592 | [0.62,  2.26] | [0.85,  1.33] |
| Age | 0.97 | 0.00 | 0.61 | -6.58 | 0.000 | [0.96,  0.98] | [0.53,  0.71] |
| Sex (1=male, 2=female) | 1.12 | 0.14 | 1.06 | 0.81 | 0.417 | [0.85,  1.47] | [0.92,  1.21] |
| Income | 0.92 | 0.03 | 0.82 | -2.52 | 0.012 | [0.86,  0.98] | [0.70,  0.96] |
| Education | 0.84 | 0.07 | 0.83 | -2.39 | 0.017 | [0.73,  0.97] | [0.71,  0.97] |
| Conservatism | 1.15 | 0.08 | 1.17 | 1.68 | 0.092 | [0.98,  1.34] | [0.97,  1.40] |
| Republican ID | 1.32 | 0.04 | 1.82 | 6.13 | 0.000 | [1.21,  1.44] | [1.50,  2.20] |
| N | | | | | | 1,430 | |
| Pseudo R2 | | | | | | 0.17 | |
| AIC | | | | | | 1355.86 | |
| BIC | | | | | | 1408.52 | |

**Supplementary Table 48: Full Regression Table for Step 4 - Approval of the January 6 insurrectionists**

|  | **b** | **SE** | **β** | **t** | **p** | **Unstandardized 95% CI** | **Standardized 95% CI** |
| --- | --- | --- | --- | --- | --- | --- | --- |
| Constant | 9.63 | 0.53 | 4.45 | 4.31 | 0.000 | [3.48,  27.36] | [3.84,  5.19] |
| White | 0.99 | 0.30 | 1.00 | -0.04 | 0.971 | [0.56,  1.68] | [0.76,  1.28] |
| Black | 1.20 | 0.33 | 1.06 | 0.55 | 0.580 | [0.62,  2.30] | [0.86,  1.31] |
| Latino | 1.15 | 0.33 | 1.05 | 0.43 | 0.666 | [0.60,  2.18] | [0.84,  1.32] |
| Age | 0.97 | 0.00 | 0.59 | -6.89 | 0.000 | [0.96,  0.98] | [0.51,  0.69] |
| Sex (1=male, 2=female) | 1.08 | 0.14 | 1.04 | 0.56 | 0.573 | [0.82,  1.42] | [0.91,  1.19] |
| Income | 0.91 | 0.03 | 0.80 | -2.80 | 0.005 | [0.85,  0.97] | [0.68,  0.93] |
| Education | 0.84 | 0.07 | 0.83 | -2.37 | 0.018 | [0.73,  0.97] | [0.71,  0.97] |
| Conservatism | 1.10 | 0.08 | 1.12 | 1.17 | 0.242 | [0.94,  1.30] | [0.93,  1.35] |
| Republican ID | 1.38 | 0.05 | 1.99 | 6.45 | 0.000 | [1.25,  1.52] | [1.62,  2.47] |
| Ideological extremism | 0.84 | 0.09 | 0.87 | -1.82 | 0.069 | [0.70,  1.01] | [0.75,  1.01] |
| Partisan extremism | 1.21 | 0.08 | 1.23 | 2.53 | 0.012 | [1.04,  1.41] | [1.05,  1.44] |
| N | | | | | | 1,430 | |
| Pseudo R2 | | | | | | 0.18 | |
| AIC | | | | | | 1352.66 | |
| BIC | | | | | | 1415.84 | |

**Supplementary Table 49: Full Regression Table for Step 5 - Approval of the January 6 insurrectionists**

|  | **b** | **SE** | **β** | **t** | **p** | **Unstandardized 95% CI** | **Standardized 95% CI** |
| --- | --- | --- | --- | --- | --- | --- | --- |
| Constant | 14.11 | 0.54 | 4.38 | 4.94 | 0.000 | [5.00,  40.94] | [3.77,  5.11] |
| White | 1.14 | 0.29 | 1.06 | 0.46 | 0.646 | [0.64,  1.96] | [0.81,  1.37] |
| Black | 1.27 | 0.34 | 1.08 | 0.71 | 0.480 | [0.65,  2.46 | [0.87,  1.34] |
| Latino | 1.28 | 0.34 | 1.09 | 0.73 | 0.468 | [0.65,  2.45] | [0.86,  1.37] |
| Age | 0.97 | 0.00 | 0.59 | -6.47 | 0.000 | [0.96,  0.98] | [0.51,  0.69] |
| Sex (1=male, 2=female) | 1.10 | 0.14 | 1.05 | 0.69 | 0.490 | [0.84,  1.46] | [0.91,  1.21] |
| Income | 0.92 | 0.03 | 0.82 | -2.50 | 0.012 | [0.85,  0.98] | [0.70,  0.96] |
| Education | 0.91 | 0.07 | 0.90 | -1.29 | 0.198 | [0.78,  1.05] | [0.77,  1.06] |
| Conservatism | 1.01 | 0.09 | 1.01 | 0.07 | 0.945 | [0.85,  1.19] | [0.83,  1.22] |
| Republican ID | 1.26 | 0.05 | 1.65 | 4.84 | 0.000 | [1.15,  1.38] | [1.35,  2.02] |
| RWA | 1.24 | 0.09 | 1.22 | 2.32 | 0.021 | [1.03,  1.48] | [1.03,  1.46] |
| SDO | 1.46 | 0.10 | 1.37 | 3.84 | 0.000 | [1.20,  1.77] | [1.17,  1.60] |
| PSJ | 1.14 | 0.09 | 1.12 | 1.50 | 0.133 | [0.96,  1.35] | [0.97,  1.29] |
| N | | | | | | 1,390 | |
| Pseudo R2 | | | | | | 0.20 | |
| AIC | | | | | | 1312.65 | |
| BIC | | | | | | 1380.73 | |

**Supplementary Table 50: Full Regression Table for Step 6 - Approval of the January 6 insurrectionists**

|  | **b** | **SE** | **β** | **t** | **p** | **Unstandardized 95% CI** | **Standardized 95% CI** |
| --- | --- | --- | --- | --- | --- | --- | --- |
| Constant | 12.96 | 0.55 | 4.46 | 4.67 | 0.000 | [4.47,  38.53] | [3.83,  5.23] |
| White | 1.11 | 0.29 | 1.05 | 0.36 | 0.718 | [0.62,  1.91] | [0.80,  1.35] |
| Black | 1.20 | 0.34 | 1.06 | 0.54 | 0.592 | [0.61,  2.33] | [0.85,  1.31] |
| Latino | 1.24 | 0.34 | 1.08 | 0.63 | 0.528 | [0.63,  2.38] | [0.85,  1.35] |
| Age | 0.97 | 0.00 | 0.58 | -6.68 | 0.000 | [0.96,  0.98] | [0.49,  0.68] |
| Sex (1=male, 2=female) | 1.06 | 0.14 | 1.03 | 0.38 | 0.705 | [0.80,  1.40] | [0.89,  1.18] |
| Income | 0.91 | 0.03 | 0.80 | -2.76 | 0.006 | [0.85,  0.97] | [0.68,  0.94] |
| Education | 0.91 | 0.07 | 0.90 | -1.29 | 0.199 | [0.78,  1.05] | [0.77,  1.06] |
| Conservatism | 0.98 | 0.09 | 0.98 | -0.25 | 0.804 | [0.82,  1.16] | [0.80,  1.19] |
| Republican ID | 1.31 | 0.05 | 1.80 | 5.24 | 0.000 | [1.19,  1.46] | [1.45,  2.25] |
| Ideological extremism | 0.87 | 0.10 | 0.89 | -1.50 | 0.134 | [0.72,  1.05] | [0.76,  1.04] |
| Partisan extremism | 1.21 | 0.08 | 1.22 | 2.43 | 0.015 | [1.04,  1.42] | [1.04,  1.44] |
| RWA | 1.22 | 0.09 | 1.21 | 2.15 | 0.031 | [1.02,  1.46] | [1.02,  1.44] |
| SDO | 1.47 | 0.10 | 1.37 | 3.86 | 0.000 | [1.21,  1.78] | [1.17,  1.61] |
| PSJ | 1.11 | 0.09 | 1.09 | 1.15 | 0.249 | [0.93,  1.31] | [0.94,  1.26] |
| N | | | | | | 1,390 | |
| Pseudo R2 | | | | | | 0.21 | |
| AIC | | | | | | 1310.41 | |
| BIC | | | | | | 1388.97 | |

**Supplementary Note 3: Results of Mediation Analysis**

**Supplementary Table 51: Results of Mediation Analysis using RWA, SDO, and PSJ as Mediators**

|  | **Legal Rights and Guarantees** | | | **Freedom of Speech** | | | **Political Equality** | | | **Defection from Rules of the Game** | | | **Willingness to vote for anti-democratic candidates** | | |
| --- | --- | --- | --- | --- | --- | --- | --- | --- | --- | --- | --- | --- | --- | --- | --- |
|  | **β**  **(SE)** | **p** | **95% CI¹** | **β**  **(SE)** | **p** | **95% CI¹** | **β**  **(SE)** | **p** | **95% CI¹** | **β**  **(SE)** | **p** | **95% CI¹** | **β**  **(SE)** | **p** | **95% CI¹** |
| **Political conservatism direct effect** | -.023  (.027) | .421 | [-.07, .03] | .244  (.027) | <.001 | [.16, .26] | -.523  (.024) | <.001 | [-.49,  -.40] | -.023  (.034) | .601 | [-.08,  .05] | .498  (.028) | <.001 | [.41,  .52] |
| **RWA direct effect** | - | - | - | - | - | - | - | - | - | .412  (.031) | <.001 | [.38,  .55] | - | - | - |
| **RWA indirect effect** | - | - | - | - | - | - | - | - | - | .277  (.022) | <.001 | [.18, .26] | - | - | - |
| **SDO direct effect** | -.422  (.035) | <.001 | [.-54, -.40] | - | - | - | -.226  (.029) | <.001 | [.-29, .-18] | .256  (.023) | <.001 | [.18, .28] | .247  (.030) | <.001 | [.21, .32] |
| **SDO indirect effect** | -.156  (.016) | <.001 | [-.18, -.12] | - | - | - | -.085  (.009) | <.001 | [-.09, -.05] | .096  (.009) | <.001 | [.06, .09] | .091  (.010) | <.001 | [.06, .10] |
| **PSJ direct effect** | .073  (.022) | <.001 | [.04, .12] | .107  (.028) | <.001 | [.05, .16] | - | - | - | -.155  (.022) | <.001 | [-.21,  -.10] | -.123  (.025) | <.001 | [-.18, -.09] |
| **PSJ indirect effect** | .014  (.004) | .002 | [.01, .02] | .021  (.005) | .001 | [.01, .03] | - | - | - | -.030  (.005) | <.001 | [-.03, -.01] | -.024  (.006) | <.001 | [-.03, -.01] |
| **Total effect** | -.166  (.023) | <.001 | [-.20, -.11] | .265  (.027) | <.001 | [.17,  .28] | -.607  (.022) | <.001 | [-.57,  -.48] | .321  (.023) | <.001 | [.21,  .30] | .566  (.024) | <.001 | [.47,  .57] |

*Note:* RWA = Right-wing authoritarianism; SDO = Social dominance orientation; PSJ = Political system justification; blank spaces = non-significant paths trimmed from the analysis. ¹ Unstandardized 95% Confidence Intervals. N = 1,557.

**Supplementary Discussion 1: Affective polarization**

We used feeling-thermometer items to gauge participants' attitudes towards six political entities: the Democratic Party; the Republican Party; Joe Biden; Donald Trump; Black Lives Matter; and the January 6, 2021, insurrectionists. Participants were asked to rate their feelings about each subject from 0 (“Cold”) to 100 (“Warm”). For the six targets under investigation, we first rescaled the variables to range from 0 to 1. We calculated descriptive statistics for each one of those political entities: the Democratic Party (Mean = .492, SD = .322); the Republican Party (Mean = .427, SD = .313); Joe Biden (Mean = .449, SD = .347); Donald Trump (Mean = .348, SD = .367); Black Lives Matter (Mean = .437, SD = .342); and the January 6, 2021, insurrectionists (Mean = .217, SD = .279). The survey also solicited feeling thermometer ratings for Bernie Sanders (who ran unsuccessfully for the Democratic presidential nomination in 2020) and Mitt Romney (who ran unsuccessfully for the presidency in 2012), but we judged that these two individuals as not especially relevant to affective polarization in the fall of 2022, so we excluded them from analysis.

For the purposes of analyzing affective polarization, we created a new binary party affiliation variable by dummy-coding the 7-point party identification variable as follows: 1 = Democratic (“Strong Democrat,” “Not so strong Democrat,” and “Lean Democrat”) and 2 = Republican (“Strong Republican,” “Not so strong Republican,” and “Lean Republican”). Respondents who chose “Don't Lean” (n = 231) were excluded from the analyses pertaining to affective polarization.

We analyzed feeling-thermometer scores for the six attitude objects separately for Democratic and Republican respondents based on the dummy-coded party affiliation variable. We also performed Mann-Whitney-Wilcoxon tests to establish that differences between respondents from the two parties were significant (i.e., that affective polarization was present). Results shown in Supplementary Figure 1 confirm that Democrats felt more warmly toward the attitude objects on the political left (Democratic Party, Joe Biden, and Black Lives Matter) than they did toward the attitude objects on the political right (Republican Party, Donald Trump, January 6 insurrectionists), whereas Republicans felt more warmly toward the attitude objects on the political right than the political left.

**Supplementary Figure 1: Feeling Thermometer Responses by Party Affiliation**


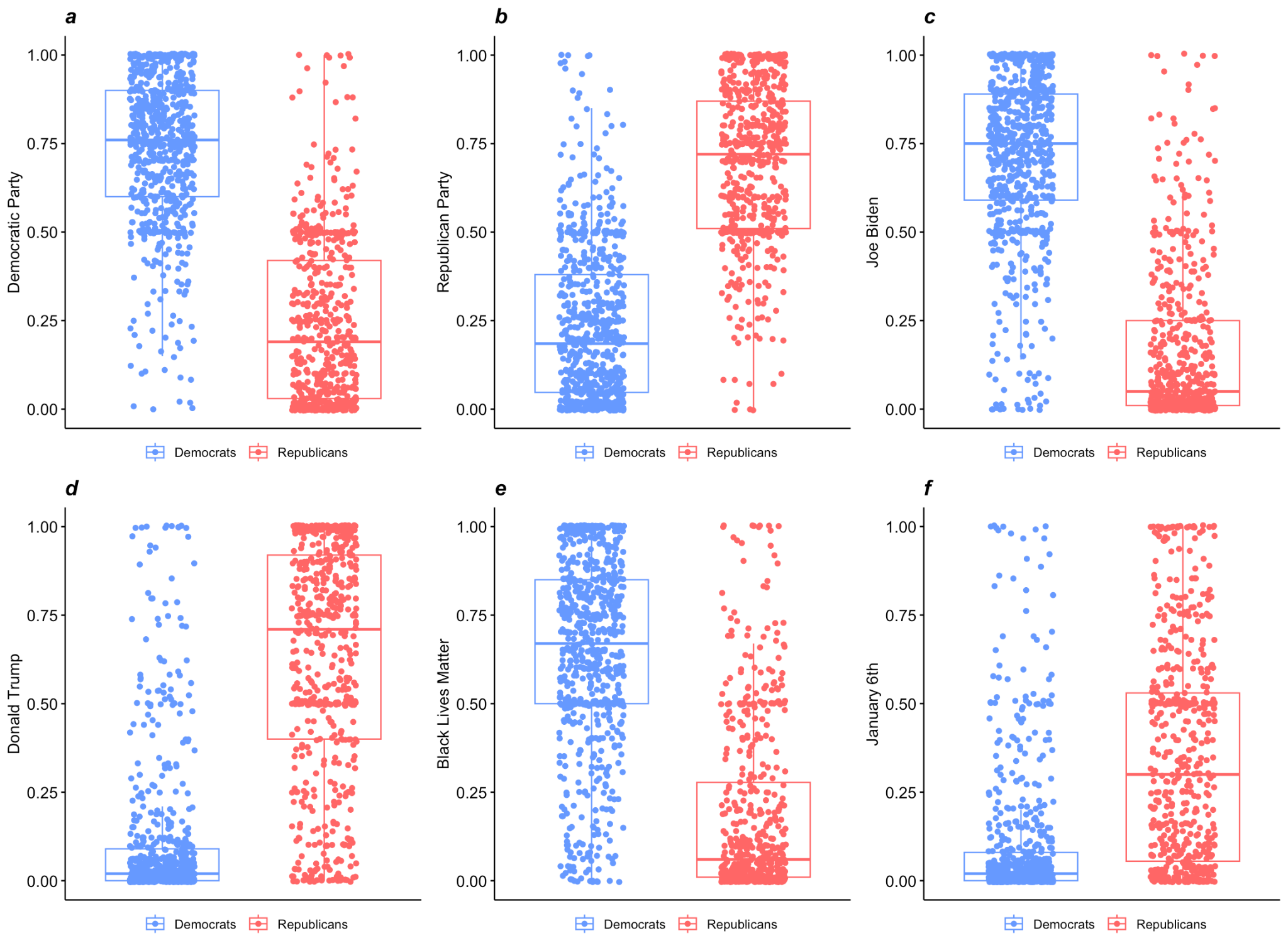


*Note:* Feeling thermometer responses by party affiliation for the following political objects: Democratic Party (**a**); Republican Party (**b**); Joe Biden (**c**); Donald Trump (**d**); Black Lives Matter supporters (**e**); and January 6th insurrectionists (**f**). Higher values indicate warmer feelings. Boxplots for those who identify with the Democratic party are colored blue and placed on the left (N = 727), and boxplots for those who identify with the Republican party are colored red and placed on the right (N = 597). Boxplots include the median in bold central line, the box representing the interquartile range, whiskers indicating 1.5 × interquartile range, and jitter dots represent data points distribution with random noise added to reduce overlapping.

Democrats evaluated the Democratic Party (Mean = .734, SD = .204), Joe Biden (Mean = .707, SD = .236), and the Black Lives Matter movement (Mean = .636, SD = .272) much more warmly than Republicans did (Democratic Party: Mean = .241, SD = .230; Joe Biden: Mean = .163, SD = .225; Black Lives Matter: Mean = .183, SD = .249). We found evidence that these differences on the evaluation of the Democratic Party (*Z* = 26.55, *p* < .001, *r* = .739), Joe Biden (*Z* = 26.15, *p* < .001, *r* = .731), and the Black Lives Matter movement (*Z* = 23.12, *p* < .001, *r* = .648) were statistically significant. On the other hand, Republicans evaluated the Republican Party (Mean = .692, SD = .219), Donald Trump (Mean = .6278, SD = .325), and the January 6th insurrectionists (Mean = .347, SD = .308) more warmly than Democrats did (Republican Party: Mean = .231, SD = .221; Donald Trump: Mean = .111, SD = .216; January 6th insurrectionists: Mean = .102, SD = .200). We found evidence that these differences on the evaluation of the Republican Party (*Z* = 25.73, *p* < .001, *r* = .719), Donald Trump (*Z* = 24.04, *p* < .001, *r* = .677), and the January 6th insurrectionists (*Z* = 16.34, *p* < .001, *r* = .463) were statistically significant.

Attitudes toward the January 6th insurrectionists are of special interest to the issue of anti-democratic sentiment. In the main text, we explore the ideological and psychological differences of Republicans and Democrats who expressed warm feelings toward January 6 comparing them with their partisan peers, testing the significance of the differences, with Mann-Whitney-Wilcoxon tests.

Then, we calculated affective polarization scores by taking the difference between in-party feelings and out-party feelings rates^1^. For Democratic respondents, affective polarization scores reflect the difference in warmth ratings toward left (vs. right) attitude objects, and for Republican respondents, affective polarization scores reflect the difference in warmth ratings toward right (vs. left) attitude objects. Higher scores indicate high in-party warm feelings and low out-party warm feelings. Results for mean scores and SD’s by party affiliation are shown in Supplementary Table 52.

**Supplementary Table 52: Mean affective polarization scores by object and party affiliation.**

|  | Party | Candidates | Movement |
| --- | --- | --- | --- |
| Republicans | .449  (*SD* = .351) | .456  (*SD* = .471) | .163  (*SD* = .396) |
| Democrats | .503  (*SD* = .318) | .598  (*SD* = .358) | .534  (*SD* = .345) |

*Note:* Mean affective polarization scores by political objects being evaluated and by party affiliation. Values in parentheses correspond to the standard deviation. N = 1,324.

As we can observe, both Democrats and Republicans are affectively polarized in terms of political parties and candidates in the 2020’s presidential elections. But in terms of political movements (namely, Black Lives Matter and January 6th insurrection), only Democrats are highly affectively polarized, whereas Republicans are not. All the different scores are statically significant (Parties: Z = 2.76, p < .01, r = .78; Candidates: Z = 4.14, p < .001, r = .12; Political Movements: Z = 15.94, p < .001, r = .45).

**Supplementary Discussion 2: Political conservatism as a mediator of the effects of RWA, SDO, and PSJ on pro- vs. anti-democratic tendencies**

In the main analyses, we tested Right-Wing Authoritarianism (RWA), Social Dominance Orientation (SDO), and Political System Justification (PSJ) as potential mediators of the effects of political conservatism. Rather than assuming a causal, developmental sequence, our mediational analyses focused on psychological underpinnings that could explain the ideological asymmetry in pro- vs. anti-democratic tendencies. In the supplementary analyses presented here, we test the Dual Process Motivational (DPM) model,^2, 3^ which suggests a different ordering.

According to the DPM model, RWA and SDO originate from distinctive personality dispositions and socialized worldviews, generating different motivational goals and values, although both contribute to right-wing conservatism. While RWA is a threat-driven motivation based on the perception of a dangerous worldview and needs for security and social cohesion^2, 3, 4^, SDO is an attitudinal orientation based on a competitive worldview and linked to preferences for hierarchy and support for inequality^2, 3, 5^. Although the DPM model does not account for the effects of system justification, it would predict that RWA and SDO shape political orientation, which would then affect pro- vs. anti-democratic tendencies.

To investigate this alternative DPM model, we conducted a Structural Equation Model using R’s “lavaan” package^6^. Path coefficients were accepted as significant at the .05 level, and nonsignificant mediation paths were trimmed. Supplementary Figure 2 illustrates this model, which provides the standardized estimates obtained as direct effects for each pathway. Because of the large sample size (*N* = 1,557), chi-square was significant χ2 (12) = 38.438, p < .001. All other goodness-of-fit indices were very good: Comparative Fit Index (CFI) = .995; Tucker-Lewis Index (TLI) = .980; Standardized Root Mean Square Residual (SRMR) = .012; Root Mean-Square Error of Approximation (RMSEA) = .038. Supplementary Table 53 displays the total effects, direct effects of political orientation, and indirect effects for each psychological factor analyzed.

**Supplementary Figure 2: Illustration of structural equation model in which political conservatism mediated the effects of psychological variables on pro- vs. anti-democratic tendencies.**


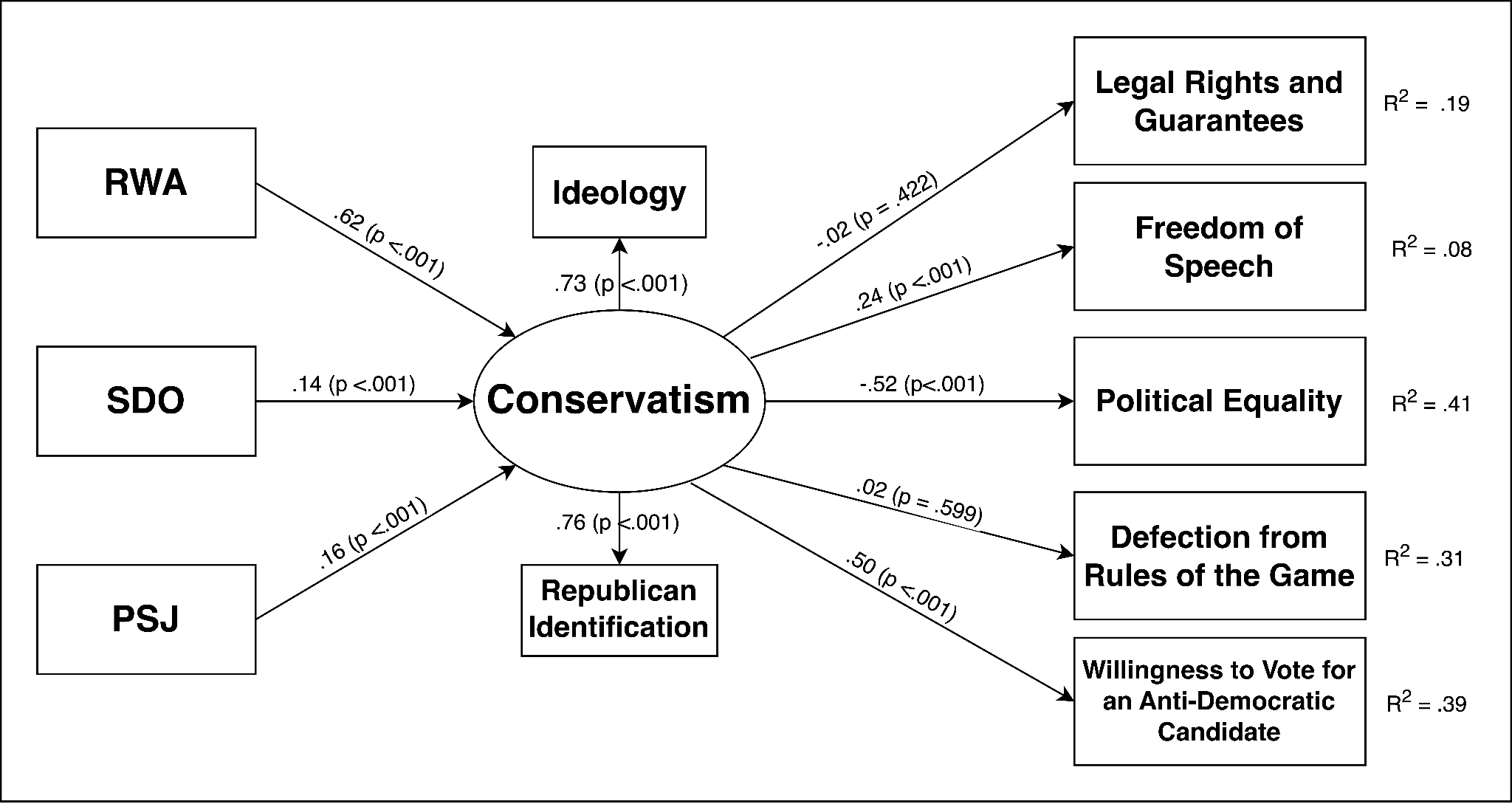


*Note*: Schematic representation of the mediation model, with conservatism mediating the effects of RWA, SDO, and PSJ on different pro- and anti-democratic tendencies. Values correspond to standardized estimates obtained as direct effects for each pathway and the p-values are in parentheses. R^2^ values are placed on the right of each pro- and anti-democratic tendency outcome. RWA = Right-wing authoritarianism; SDO = Social dominance orientation; PSJ = Political system justification. N = 1,557.

As shown in Supplementary Figure 2, RWA, SDO, and PSJ were positively associated with conservatism. In turn, conservatism was negatively associated with political equality and positively associated with willingness to vote for an anti-democratic candidate and support for freedom of speech. However, it was unrelated to support for legal rights and guarantees and defection from the rules of the game. Conservatism significantly mediated the effects of SDO on the rejection of political equality and willingness to vote for anti-democratic candidates, as well as the effects of PSJ on support for freedom of speech and willingness to vote for anti-democratic candidates (see Supplementary Table 53). Conservatism did not significantly mediate any effects of RWA.

**Supplementary Discussion 3: Mediation analyses with political extremism**

We also ran Structural Equation Models, using R’s “lavaan” package^6^, to determine whether the direct and indirect effects described in the main text were altered by adjusting for the effects of ideological and partisan extremism. In both models, path coefficients were accepted as significant at the .05 level, and nonsignificant mediation paths were trimmed.

First, we re-ran the model presented in the main text, in which the effects of conservatism are mediated by RWA, SDO, and PSJ. Because of the large sample size (*N* = 1,557), chi-square was significant χ2 (12) = 34.692, p < .001. All other goodness-of-fit indices were very good: Comparative Fit Index (CFI) = .996; Tucker-Lewis Index (TLI) = .977; Standardized Root Mean Square Residual (SRMR) = .010; Root Mean-Square Error of Approximation (RMSEA) = .035. Results for this analysis are shown in Supplementary Table 54.

As before, respondents with a more conservative orientation were higher in RWA, SDO, and PSJ. RWA was again positively associated with willingness to defect from the rules of the game and was a significant mediator of the effect of conservatism. SDO was again positively associated with willingness to defect from the democratic rules of the game and to vote for blatantly anti-democratic candidates, and it was negatively associated with support for political equality and legal rights and guarantees. In all cases, SDO was again a significant mediator of the effects of conservatism. PSJ was again positively associated with support for freedom of speech as well as legal rights and guarantees and negatively associated with willingness to defect from the rules of the game and to vote for anti-democratic candidates. In all cases, PSJ was again a significant mediator of the effects of conservatism. The total effects for legal rights and guarantees were not statistically significant in this model, which departs from the results described in the main text. However, even after adjusting for ideological and partisan extremism, we obtained consistent evidence that: (a) conservatives harbored more anti-democratic tendencies than liberals and that these differences were attributable in part to the fact that they were higher in RWA and SDO; and (b) conservatives were higher than liberals on PSJ, and PSJ exerted an attenuating influence on anti-democratic tendencies.

Finally, we tested the alternative DPM model while adjusting for political extremity. Because of the large sample size (*N* = 1,557), chi-square was significant χ2 (12) = 34.890, p < .001. All other goodness-of-fit indices were very good: Comparative Fit Index (CFI) = .996; Tucker-Lewis Index (TLI) = .977; Standardized Root Mean Square Residual (SRMR) = .010; Root Mean-Square Error of Approximation (RMSEA) = .035. The results for this analysis are shown in Supplementary Table 55.

Again, RWA, SDO, and PSJ were positively associated with political conservatism. As in the mediational analyses without extremism (see Supplementary Figure 2), conservatism significantly mediated the effects of SDO on the rejection of political equality and willingness to vote for anti-democratic candidates, as well as the effects of PSJ on support for freedom of speech and willingness to vote for anti-democratic candidates. Thus, adjusting for the effects of ideological and partisan extremism did not substantially change any of the interpretations.

**Supplementary Table 53: Results of Mediation Analysis using Political Conservatism as Mediator**

|  | **Legal Rights and Guarantees** | | | **Freedom of Speech** | | | **Political Equality** | | | **Defection from Rules of the Game** | | | **Willingness to vote for anti-democratic candidates** | | |
| --- | --- | --- | --- | --- | --- | --- | --- | --- | --- | --- | --- | --- | --- | --- | --- |
|  | **β**  **(SE)** | **p** | **95% CI¹** | **β**  **(SE)** | **p** | **95% CI¹** | **β**  **(SE)** | **p** | **95% CI¹** | **β**  **(SE)** | **p** | **95% CI¹** | **β**  **(SE)** | **p** | **95% CI¹** |
| **RWA direct effect** | - | - | - | - | - | - | - | - | - | .411 (.031) | < .001 | [.28,  .40] | - | - | - |
| **SDO direct effect** | -.421  (.034) | < .001 | [-.54,  -.40] | - | - | - | -.228 (.029) | < .001 | [-.29,  -.18] | .259  (.023) | < .001 | [.20,  .30] | .246 (.030) | < .001 | [.21,  .33] |
| **PSJ direct effect** | .073  (.022) | < .001 | [.04,  .12] | .107 (.028) | < .001 | [.05,  .16] | - | - | - | -.155  (.022) | < .001 | [-.19, -.10] | -.123  (.025) | < .001 | [-.18,  -.09] |
| **Political conservatism direct effect** | -.023  (.019) | .422 | [.-05, .02] | .244 (.019) | < .001 | [.11,  .19] | -.523  (.018) | < .001 | [.-35, .-28] | -.023  (.024) | .599 | [-.06, .03] | .498  (.020) | < .001 | [.28, .36] |
| **Political conservatism indirect effect - RWA** | - | - | - | - | - | - | - | - | - | -.014  (.022) | .602 | [-.05, .03] | - | - | - |
| **Political conservatism indirect effect - SDO** | -.003  (.005) | .438 | [-.01, .01] | - | - | - | -.072 (.017) | < .001 | [-.11,  -.04] | -.003  (.006) | .614 | [-.01,  .01] | .069  (.017) | < .001 | [.04, .11] |
| **Political conservatism indirect effect - PSJ** | -.004  (.005) | .427 | [-.01, .01] | .040  (.009) | < .001 | [.02, .06] | - | - | - | -.004  (.007) | .608 | [-.02, .01] | .081  (.015) | < .001 | [.06, .12] |
| **Total effect** | -.355  (.040) | < .001 | [-.48, -.32] | .146  (.029) | < .001 | [.09,  .20] | -.299  (.026) | < .001 | [-.36,  -.26] | .494  (.031) | < .001 | [.37,  .49] | .272  (.037) | < .001 | [.23,  .38] |

*Note:* RWA = Right-wing authoritarianism; SDO = Social dominance orientation; PSJ = Political system justification; blank spaces = non-significant paths trimmed from the analysis. ¹ Unstandardized 95% Confidence Intervals. N = 1,557.

**Supplementary Table 54: Results of Mediation Analysis using RWA, SDO, and PSJ as Mediators adjusting for ideological and partisan extremism**

|  | **Legal Rights and Guarantees** | | | **Freedom of Speech** | | | **Political Equality** | | | **Defection from Rules of the Game** | | | **Willingness to vote for anti-democratic candidates** | | |
| --- | --- | --- | --- | --- | --- | --- | --- | --- | --- | --- | --- | --- | --- | --- | --- |
|  | **β**  **(SE)** | **p** | **95% CI¹** | **β**  **(SE)** | **p** | **95% CI¹** | **β**  **(SE)** | **p** | **95% CI¹** | **β**  **(SE)** | **p** | **95% CI¹** | **β**  **(SE)** | **p** | **95% CI¹** |
| **Political conservatism direct effect** | -.022  (.026) | .446 | [-.07, .03] | .236  (.027) | <.001 | [.15, .25] | -.522  (.024) | <.001 | [-.49,  -.40] | -.015  (.035) | .743 | [-.08,  .06] | .497  (.028) | <.001 | [.40,  0.51] |
| **RWA direct effect** | - | - | - | - | - | - | - | - | - | .405  (.032) | <.001 | [.28,  .40] | - | - | - |
| **RWA indirect effect** | - | - | - | - | - | - | - | - | - | .276  (.022) | <.001 | [.18, .26] | - | - | - |
| **SDO direct effect** | -.403  (.034) | <.001 | [.-52, -.39] | - | - | - | -.214  (.029) | <.001 | [.-28, .-16] | .259  (.024) | <.001 | [.20, .30] | .250  (.031) | <.001 | [.22, .34] |
| **SDO indirect effect** | -.152  (.016) | <.001 | [-.17, -.11] | - | - | - | -.081  (.009) | <.001 | [-.09, -.05] | .098  (.010) | <.001 | [.06, .10] | .094  (.010) | <.001 | [.07, .11] |
| **PSJ direct effect** | .066  (.022) | <.001 | [.03, .11] | .119  (.028) | <.001 | [.06, .17] | - | - | - | -.159  (.022) | <.001 | [-.19,  -.10] | -.124  (.025) | <.001 | [-.18, -.09] |
| **PSJ indirect effect** | .013  (.004) | .003 | [.00, .02] | .024  (.006) | .001 | [.01, .03] | - | - | - | -.032  (.006) | <.001 | [-.04, -.01] | -.025  (.006) | <.001 | [-.04, -.01] |
| **Total effect** | -.105  (.036) | .643 | [-.09, .05] | .252  (.041) | <.001 | [.15,  .31] | -.524  (.040) | <.001 | [-.54,  -.39] | .324  (.033) | <.001 | [.19,  .31] | .635  (.041) | <.001 | [.52,  .68] |

*Note:* RWA = Right-wing authoritarianism; SDO = Social dominance orientation; PSJ = Political system justification; blank spaces = non-significant paths trimmed from the analysis. ¹ Unstandardized 95% Confidence Intervals. N = 1,557.

**Supplementary Table 55: Results of Mediation Analysis using Political Conservatism as Mediator adjusting for ideological and partisan extremism**

|  | **Legal Rights and Guarantees** | | | **Freedom of Speech** | | | **Political Equality** | | | **Defection from Rules of the Game** | | | **Willingness to vote for anti-democratic candidates** | | |
| --- | --- | --- | --- | --- | --- | --- | --- | --- | --- | --- | --- | --- | --- | --- | --- |
|  | **β**  **(SE)** | **p** | **95% CI¹** | **β**  **(SE)** | **p** | **95% CI¹** | **β**  **(SE)** | **p** | **95% CI¹** | **β**  **(SE)** | **p** | **95% CI¹** | **β**  **(SE)** | **p** | **95% CI¹** |
| **RWA direct effect** | - | - | - | - | - | - | - | - | - | .405 (.032) | < .001 | [.28,  .40] | - | - | - |
| **SDO direct effect** | -.404  (.034) | < .001 | [-.52,  -.39] | - | - | - | -.216 (.029) | < .001 | [-.28,  -.17] | .259  (.024) | < .001 | [.20,  .30] | .252 (.030) | < .001 | [.22,  .34] |
| **PSJ direct effect** | .066  (.022) | < .001 | [.03,  .12] | .119 (.028) | < .001 | [.06,  .17] | - | - | - | -.159  (.022) | < .001 | [-.19, -.10] | -.125  (.025) | < .001 | [-.18,  -.09] |
| **Political conservatism direct effect** | -.021  (.018) | .523 | [.-05, .02] | .236 (.019) | < .001 | [.10,  .18] | -.522  (.018) | < .001 | [.-35, .-28] | -.015  (.025) | .743 | [-.06, .04] | .497  (.020) | < .001 | [.28, .36] |
| **Political conservatism indirect effect - RWA** | - | - | - | - | - | - | - | - | - | .009  (.023) | .746 | [-.05, .04] | - | - | - |
| **Political conservatism indirect effect - SDO** | -.003  (.005) | .469 | [-.01, .01] | - | - | - | -.073 (.017) | < .001 | [-.11,  -.04] | .002  (.006) | .752 | [-.01,  .01] | .070  (.017) | < .001 | [.04, .11] |
| **Political conservatism indirect effect - PSJ** | -.004  (.005) | .459 | [-.01, .01] | .041  (.009) | < .001 | [.03, .06] | - | - | - | .002  (.007) | .749 | [-.02, .01] | .085  (.015) | < .001 | [.06, .12] |
| **Total effect** | -.199  (.048) | < .001 | [-.35, -.16] | .162  (.040) | < .001 | [.10,  .26] | -.233  (.040) | < .001 | [-.35,  -.19] | .487  (.040) | < .001 | [.34,  .49] | .371  (.048) | < .001 | [.32,  .51] |

*Note:* RWA = Right-wing authoritarianism; SDO = Social dominance orientation; PSJ = Political system justification; blank spaces = non-significant paths trimmed from the analysis. ¹ Unstandardized 95% Confidence Intervals. N = 1,557.

**Supplementary References**

1. Iyengar, S., Sood, G. & Lelkes, Y. Affect, not Ideology: a Social Identity Perspective on Polarization. Public Opin. Quart. 76, 405–431 (2012). <https://doi.org/10.1093/poq/nfs038>
2. Duckitt, J. & Sibley, C. G. The dual process motivational model of ideology and prejudice. in *The Cambridge handbook of the psychology of prejudice* (ed. Sibley, C. G. & Barlow, F. K.) 188–221 (Cambridge Univ. Press, 2017).
3. Duckitt, J. & Sibley, C. G. Personality, ideology, prejudice, and politics: a dual process motivational model. *J. Pers.* 78, 1861–1893 (2010). <https://doi.org/10.1111/j.1467-6494.2010.00672.x>
4. Altemeyer, B. The other authoritarian personality. in *Advances in experimental social psychology* (ed. Zanna, M. P.) 47-92 (Academic Press, San Diego, 1998).
5. Pratto, F., Sidanius, J., Stallworth, L. M. & Malle, B. F. Social dominance orientation: A personality variable predicting social and political attitudes. *J. Pers. Soc. Psychol.* 67, 741–763 (1994). <https://doi.org/10.1037/0022-3514.67.4.741>
6. Rosseel Y. “lavaan: An R Package for Structural Equation Modeling.” *Stat. Softw.* 48, 1–36 (2012). <https://doi.org/10.18637/jss.v048.i02>
