# Supplementary material for: Liberal-conservative asymmetries in anti-democratic tendencies are partly explained by psychological differences in a nationally representative U.S. sample
Source: Commun Psychol. 2024 Jul 2;2:61. doi: 10.1038/s44271-024-00096-3 (PMC11332046; doi:10.1038/s44271-024-00096-3)
Supplement: Supplementary file 3 — Reporting Summary [file 44271_2024_96_MOESM3_ESM.pdf]

nature portfolio

Corresponding author(s): Débora de Oliveira Santos

Last updated by author(s): Mar 19, 2024

Reporting Summary

Nature Portfolio wishes to improve the reproducibility of the work that we publish. This form provides structure for consistency and transparency in reporting. For further information on Nature Portfolio policies, see our Editorial Policies and the Editorial Policy Checklist.

Statistics

For all statistical analyses, confirm that the following items are present in the figure legend, table legend, main text, or Methods section.

n/a	Confirmed	
☐	☒	The exact sample size (*n*) for each experimental group/condition, given as a discrete number and unit of measurement
☐	☒	A statement on whether measurements were taken from distinct samples or whether the same sample was measured repeatedly
☐	☒	The statistical test(s) used AND whether they are one- or two-sided<br>*Only common tests should be described solely by name; describe more complex techniques in the Methods section.*
☐	☒	A description of all covariates tested
☐	☒	A description of any assumptions or corrections, such as tests of normality and adjustment for multiple comparisons
☐	☒	A full description of the statistical parameters including central tendency (e.g. means) or other basic estimates (e.g. regression coefficient) AND variation (e.g. standard deviation) or associated estimates of uncertainty (e.g. confidence intervals)
☐	☒	For null hypothesis testing, the test statistic (e.g. F, t, r) with confidence intervals, effect sizes, degrees of freedom and P value noted<br>*Give P values as exact values whenever suitable.*
☒	☐	For Bayesian analysis, information on the choice of priors and Markov chain Monte Carlo settings
☐	☒	For hierarchical and complex designs, identification of the appropriate level for tests and full reporting of outcomes
☐	☒	Estimates of effect sizes (e.g. Cohen's *d*, Pearson's *r*), indicating how they were calculated

Our web collection on statistics for biologists contains articles on many of the points above.

Software and code

Policy information about availability of computer code

Data collection	All data used in this article come from the Health of Democracy Survey, commissioned by the University of Notre Dame and carried out by the National Opinion Research Center (NORC) at the University of Chicago in connection with the AmeriSpeak® Panel. The survey was took online through the AmeriSpeak® Mobile App, the password-protected AmeriSpeak® Web portal, or by following a link in the email invitation.
Data analysis	For the data analysis, we used both SPSS and R. SPSS 25 was used for the factorial analysis. R version 4.2.2 was used for descriptive statistics, correlation tests, hierarchical regressions, mediational analyses, and group comparisons. Detailed information about the code used in R can be accessed at: https://osf.io/92ezy/.

For manuscripts utilizing custom algorithms or software that are central to the research but not yet described in published literature, software must be made available to editors and reviewers. We strongly encourage code deposition in a community repository (e.g. GitHub). See the Nature Portfolio guidelines for submitting code & software for further information.

Data

Policy information about availability of data

All manuscripts must include a data availability statement. This statement should provide the following information, where applicable:

- Accession codes, unique identifiers, or web links for publicly available datasets
- A description of any restrictions on data availability
- For clinical datasets or third party data, please ensure that the statement adheres to our policy

All data used in this article come from the Health of Democracy Survey, commissioned by the University of Notre Dame and carried out by the National Opinion Research Center (NORC) at the University of Chicago in connection with the AmeriSpeak® Panel. The data set is privately owned by the Rooney Center at the University of Notre Dame and is not yet publicly available, but it will be made publicly available online in April 2025 through a website for the Health of Democracy Survey.

Human research participants

Policy information about studies involving human research participants and Sex and Gender in Research.

Reporting on sex and gender	The Health of Democracy Survey accounted for sex (female and male). Since the NORC's AmeriSpeak® Panel was the sample source, participants recruited were asked to state their own sex (self-reporting) prior to the survey being carried out and this information was included into the survey data. Our hierarchical regression analyses accounted for sex in Steps 1, 3, 4, 5, and 6.
Population characteristics	The Health of Democracy survey targeted the U.S. general adult population aged above 18 years old and it was designed to be a nationally representative sample. The final sample had 1,557 observation (Mean age: 52; Race/Ethnicity: 65.8% Non-Hispanic White, 12.4% Non-Hispanic Black, 15% Hispanic, 3.02% Asian, 2.57% 2+ races Non-Hispanic, 1.22% Other Non-Hispanic; Sex: 49% Male Respondents and 51% Female Respondents).
Recruitment	6,509 panelists from the NORC's AmeriSpeak® Panel were invited to the Health of Democracy Survey. The recruitment for the NORC's AmeriSpeak Panel is based on household level. For the Health of Democracy Survey, the 6,509 panelists were initially sampled and received an invitation from NORC as well as email reminders to take the survey online through the AmeriSpeak® Mobile App, the password-protected AmeriSpeak® Web portal, or by following a link in the email invitation. To incentive participation, panelists initially sampled were offered equivalent of $4.00 for completing the survey.
Ethics oversight	The Health of Democracy Survey was commissioned by the University of Notre Dame and carried out by the National Opinion Research Center (NORC) at the University of Chicago in connection with the AmeriSpeak® Panel and following AAPOR's Transparency Initiative.

Note that full information on the approval of the study protocol must also be provided in the manuscript.

Field-specific reporting

Please select the one below that is the best fit for your research. If you are not sure, read the appropriate sections before making your selection.

☐ Life sciences ☒ Behavioural & social sciences ☐ Ecological, evolutionary & environmental sciences

For a reference copy of the document with all sections, see nature.com/documents/nr-reporting-summary-flat.pdf

Behavioural & social sciences study design

All studies must disclose on these points even when the disclosure is negative.

Study description	It is a quantitative correlational study on ideological asymmetries and psychological differences in orientations towards democracy.
Research sample	The data used in the study comes from the Health of Democracy Survey, commissioned by the University of Notre Dame and carried out by the National Opinion Research Center (NORC) at the University of Chicago in connection with the AmeriSpeak® Panel. The survey was based on a nationally representative sample of U.S. general population over 18 years old from NORC's AmeriSpeak Panel. Overall, the purpose of the survey was measuring public opinion on American democratic institutions as well as reasons for how their attitudes were developed.
Sampling strategy	The NORC's AmeriSpeak® Panel was the sample source for the Health of Democracy Survey. The sample was selected using sampling strata based on age, race/ethnicity, education, and gender (48 sampling strata in total). Sample selection took into account the expected differential survey completion rates across the sampling strata. The size of the selected sample per stratum was determined by complete surveys across the strata matching the target population represented by census data. A initial sample of 6,509 individuals panelists at the NORC's AmeriSpeak® Panel were first selected to compose the study sample with the equivalent of

$4.00 incentive to complete the survey. The final analytic sample was composed of 1,557 observations, considering those that accepted and completed the survey and after removing 78 observations due to data quality parameters.

Data collection	The survey was self-administered by the respondent online via the Web and it was offered in English and Spanish.
Timing	From October 18, 2022 to October 26, 2022.
Data exclusions	From the democratic principles battery, we excluded the item "Two adults who love each other should be allowed to get married, regardless of sexual orientation" from the analysis. We excluded it because we expected that it would be strongly correlated with ideological self-placement for reasons that may or may not have to do with anti-democratic sentiment.<br>From the beliefs about a U.S. political crisis, we excluded the item "Joe Biden and the Democrats stole the 2020 presidential election" from the analysis. We excluded it because it contained explicit political content that would obviously be more agreeable to Republicans than Democrats. The second factor generated from the factor analysis on the battery of questions that captured beliefs about an American political crisis was also excluded, because (as noted by an anonymous reviewer) the second factor was not unambiguously related to anti-democratic tendencies, but rather to beliefs about an American crisis.<br>From the feeling thermometer battery, we excluded the items about (who ran unsuccessfully for the Democratic presidential nomination in 2020) and Mitt Romney (who ran unsuccessfully for the presidency in 2012). We judged that these two individuals as not especially relevant to affective polarization in the fall of 2022.
Non-participation	A initial sample of 6,509 individuals were first selected to compose the study sample The final analytic sample was composed of 1,557 observations, considering completed the survey and after removing 78 observations due to data quality parameters.
Randomization	Participants were not allocated into experimental groups.

Reporting for specific materials, systems and methods

We require information from authors about some types of materials, experimental systems and methods used in many studies. Here, indicate whether each material, system or method listed is relevant to your study. If you are not sure if a list item applies to your research, read the appropriate section before selecting a response.

Materials & experimental systems

n/a	Involved in the study	
☒	☐	Antibodies
☒	☐	Eukaryotic cell lines
☒	☐	Palaeontology and archaeology
☒	☐	Animals and other organisms
☒	☐	Clinical data
☒	☐	Dual use research of concern

Methods

n/a	Involved in the study	
☒	☐	ChIP-seq
☒	☐	Flow cytometry
☒	☐	MRI-based neuroimaging
