# Supplementary material for: Liberal-conservative asymmetries in anti-democratic tendencies are partly explained by psychological differences in a nationally representative U.S. sample
Source: Commun Psychol. 2024 Jul 2;2:61. doi: 10.1038/s44271-024-00096-3 (PMC11332046; doi:10.1038/s44271-024-00096-3)
Supplement: Supplementary file 2 — Peer Review File [file 44271_2024_96_MOESM2_ESM.pdf]

nature portfolio

Peer Review File

Liberal-conservative asymmetries in anti-democratic tendencies are partly explained by psychological differences in a nationally representative U.S. sample

Open Access This file is licensed under a Creative Commons Attribution 4.0 International License, which permits use, sharing, adaptation, distribution and reproduction in any medium or format, as long as you give appropriate credit to the original author(s) and the source, provide a link to the Creative Commons license, and indicate if changes were made. In the cases where the authors are anonymous, such as is the case for the reports of anonymous peer reviewers, author attribution should be to 'Anonymous Referee' followed by a clear attribution to the source work. The images or other third party material in this file are included in the article's Creative Commons license, unless indicated otherwise in a credit line to the material. If material is not included in the article's Creative Commons license and your intended use is not permitted by statutory regulation or exceeds the permitted use, you will need to obtain permission directly from the copyright holder. To view a copy of this license, visit http://creativecommons.org/licenses/by/4.0/.

Web links to the author's journal account have been redacted from the decision letters as indicated to maintain confidentiality

16th Nov 23

Dear Ms de Oliveira Santos,

Thank you for your patience during the peer-review process. Your manuscript titled "A Political Psychological Analysis of Ideological Asymmetries in Anti-Democratic Tendencies in the U.S." has now been seen by 3 reviewers, and I include their comments at the end of this message. They find your work of interest, but raised some important points. We are interested in the possibility of publishing your study in Communications Psychology, but would like to consider your responses to these concerns and assess a revised manuscript before we make a final decision on publication.

We therefore invite you to revise and resubmit your manuscript, along with a point-by-point response to the reviewers. Please highlight all changes in the manuscript text file.

In response to the concerns voiced by the reviewers, we ask that you conduct the recommended additional analysis (which should be in the main manuscript), ensure that the rationale for each measurement/analysis choice is sufficiently clear, and that important limitations, such as those pertaining to validity and alternative interpretations are discussed transparently in your limitations section in the Discussion.

We are committed to providing a fair and constructive peer-review process. Please don't hesitate to contact us if you wish to discuss the revision in more detail.

Please note that your revised manuscript must comply with our formatting and reporting requirements, which are summarized on the following checklist: Communications Psychology formatting checklist and also in our style and formatting guide Communications Psychology formatting guide .

We emphasize that failure to comply with our guidelines, in particular with regard to the manuscript section order, statistics reporting, and interpretation may mean that the work is returned to you prior to re-review, as these issues frequently lead to delays if not addressed in the first revision stage.

Please note that our guidelines contain specific recommendations for the reporting of sex and gender (which Reviewer #2 refers to). You will find these linked in the Reporting Summary and on our policy pages. We expect all manuscripts to contain a preregistration statement (which also means that if no preregistration was present, this should be stated).

Please use the following link to submit your revised manuscript, point-by-point response to the referees' comments (which should be in a separate document to any cover letter) and the completed checklist:
[Link redacted]

** This url links to your confidential home page and associated information about manuscripts you may have submitted or be reviewing for us. If you wish to forward this email to co-authors, please delete the link to your homepage first **

We hope to receive your revised paper within 8 weeks; please let us know if you aren't able to submit it within this time so that we can discuss how best to proceed. If we don't hear from you, and the revision process takes significantly longer, we may close your file. In this event, we will still be happy to reconsider your paper at a later date, provided it still presents a significant contribution to the

literature at that stage.

We would appreciate it if you could keep us informed about an estimated timescale for resubmission, to facilitate our planning.

Please do not hesitate to contact me if you have any questions or would like to discuss these revisions further. We look forward to seeing the revised manuscript and thank you for the opportunity to review your work.

Best regards,

Antonia Eisenkoeck

Antonia Eisenkoeck
Senior Editor
Communications Psychology

EDITORIAL POLICIES AND FORMATTING

We ask that you ensure your manuscript complies with our editorial policies. Please ensure that the following formatting requirements are met, and any checklist relevant to your research is completed and uploaded as a Related Manuscript file type with the revised article.

Editorial Policy: Policy requirements (Download the link to your computer as a PDF.)

* TRANSPARENT PEER REVIEW: Communications Psychology uses a transparent peer review system. This means that we publish the editorial decision letters including Reviewers' comments to the authors and the author rebuttal letters online as a supplementary peer review file. However, on author request, confidential information and data can be removed from the published reviewer reports and rebuttal letters prior to publication. If your manuscript has been previously reviewed at another journal, those Reviewers' comments would not form part of the published peer review file.

* CODE AVAILABILITY: All Communications Psychology manuscripts must include a section titled "Code Availability" at the end of the methods section. In the event of publication, we require that the custom analysis code supporting your conclusions is made available in a publicly accessible repository; at publication, we ask you to choose a repository that provides a DOI for the code; the link to the repository and the DOI will need to be included in the Code Availability statement. Publication as Supplementary Information will not suffice. We ask you to prepare code at this stage, to avoid delays later on in the process.

* DATA AVAILABILITY:
All Communications Psychology manuscripts must include a section titled "Data Availability" at the end of the Methods section or main text (if no Methods). More information on this policy, is available at http://www.nature.com/authors/policies/data/data-availability-statements-data-citations.pdf.

At a minimum the Data availability statement must explain how the data can be obtained and whether there are any restrictions on data sharing. Communications Psychology strongly endorses open sharing of data. If you do make your data openly available, please include in the statement:

- Unique identifiers (such as DOIs and hyperlinks for datasets in public repositories)
- Accession codes where appropriate
- If applicable, a statement regarding data available with restrictions
- If a dataset has a Digital Object Identifier (DOI) as its unique identifier, we strongly encourage including this in the Reference list and citing the dataset in the Data Availability Statement.

We recommend submitting the data to discipline-specific, community-recognized repositories, where possible and a list of recommended repositories is provided at http://www.nature.com/sdata/policies/repositories.

If a community resource is unavailable, data can be submitted to generalist repositories such as figshare or Dryad Digital Repository. Please provide a unique identifier for the data (for example a DOI or a permanent URL) in the data availability statement, if possible. If the repository does not provide identifiers, we encourage authors to supply the search terms that will return the data. For data that have been obtained from publicly available sources, please provide a URL and the specific data product name in the data availability statement. Data with a DOI should be further cited in the methods reference section.

Please refer to our data policies at http://www.nature.com/authors/policies/availability.html.

REVIEWERS' EXPERTISE:

Reviewer #1: polarization in US
Reviewer #2: polarization in US, democratic beliefs
Reviewer #3: democratic beliefs

REVIEWERS' COMMENTS:

Reviewer #2 (Remarks to the Author):

the aim of this paper is to analyse anti-democratic tendencies from a psychological perspective in a representative sample of US adults. The findings are consistent with the previous studies and help disentangle the role of ideological differences. Both Democrats and Republicans who supported the attack on the US Capitol had higher RWA and SDO scores than those who did not. Moreover, the PSJ, which is associated with the status quo, is a significant mediator on all issues related to system stability.
The authors clearly explain the limitations of this survey, but they discuss very deeply the psychological implication underlying the transformation of the political scenario, i.e. the simple dicothom.
the paper is very well written and the contribution to the field very relevant. The analyses explained accurately. It is ready to be published.

Reviewer #3 (Remarks to the Author):

There is much to like about the paper “A Political Psychological Analysis of Ideological Asymmetries in Anti-Demographic Tendencies in the U.S”. The authors explore an important and timely research question, use a nationally representative sample from a politically polarized country, and explore their research questions in a context where anti-democratic attitudes have risen. Nevertheless, I have several questions about some of the choices the authors made.

I agree that political conservatism (identity, self-placement), RWA, SDO, and system justification are relevant to understanding anti-democratic attitudes. However, as the paper stands, it is not entirely clear or fully explained why the authors do. First, the reason for the particular ordering of these variables in the present paper is unclear (I'm not necessarily suggesting that the authors are wrong – more so that the rationale for their ordering is absent from their paper and therefore it's difficult to evaluate). As one example, the Dual Process Model (Duckitt, 2001) positions RWA and SDO as predictors of conservative attitudes and self-placement rather than vice versa. Other research has used measures of RWA, SDO, and system justification as measures of conservatism (e.g. Jost et al., 2003) (for what it's worth, I disagree that these are synonymous with conservatism; see also e.g. Stenner, 2005 – and therefore it makes sense to separate these constructs).

Second, the paper could benefit from additional rationale for why RWA, SDO, and system justification specifically beyond a single sentence on page 2 (lines 76-77) that says "… RWA and SDO are both associated with decreased commitment to basic democratic norms and principles". Adding some information about these constructs would help to appreciate why these are relevant and relate to anti-democratic attitudes, and why they might be positioned as mediators – especially given that this is at odds with some widely accepted models and conceptualizations of RWA, SDO, SJPs and conservatism.

If the authors wish to tackle the asymmetry question, I was surprised to not see an exploration of a potential curvilinear relationship, for instance, to potentially more directly evaluate whether the extremes are more anti-democratic. I was surprised to not see more mention of research that might argue for a curvilinear relationship to situate the research in more broadly (e.g. van Prooijen & Krouwel, 2019; but see also Palaiologou, 2019, Adler, 2018). For example, maybe the extremes are less democratic, but the relationship is stronger on the conservative side than the liberal side, or for particular participants (Democrats, Republicans, those that supported vs. opposed the insurrection).

Apologies if I'm just missing this, but were the hypotheses pre-registered? Or is it just that the data is available?

Were the options for gender female/male? These terms are typically reserved for sex with man/woman typically being used for gender. Were other gender options available?

Could the authors add the R2 values to the figures?

Could the authors include the 95% confidence intervals?

I appreciate the potential value in focusing on participants who approved vs. disapproved of the January 6 insurrection; however, drawing conclusions from samples of 40, 19, or 47 participants has some challenges – even with a representative sample. I would encourage the authors to, at a minimum, remind readers of the limitations of these types of comparisons; and potentially, present these analyses as more descriptive.

Reviewer #4 (Remarks to the Author):

This paper examines the relationship between ideology and support for democracy in the United States, using a wide variety of measures of the latter. It does so under the backdrop of a rise in polarization and anti-democratic extremism in the United States, as exemplified by the January 6th Capitol attacks.

In terms of raw correlations , conservatism was associated with anti-democratic outcomes, including lower support for legal rights and guarantees, lower support for political equality, higher tolerance for defection from rules of the game, higher willingness to use political violence, and higher willingness to vote for an anti-democratic candidate. At the same time, conservatism was also associated with higher support for free speech and unassociated with tolerance for disliked groups. These associations tend to remain after controlling for party affiliation and several demographic variables, but weaken or disappear after controlling for right-wing authoritarianism (RWA), social dominance orientation (SDO), and political system justification (PSJ). In other words, the balance of evidence supports the assertion that conservatism is associated with higher endorsement of anti-democratic outcomes.

Using a more complex SEM in which conservatism (as measured via ideology and party identification) affects RWA, SDO, and PSJ, which each in turn affect anti-democratic outcomes, a more complex picture emerges. Conservatism, as mediated by RWA and SDO, tends to wither away support for democracy and promote support for anti-democratic extremism. However, conservatism, as mediated by political system justification, has the opposite effect.

This work is competently done, using a large, nationally representative sample. They incorporate appropriate psychological variables as predictors. While most of the scales examining attitudes toward democracy are not established scales, they are face-valid and the authors, through factor analysis, demonstrate a clear need for separate measures. With the exception of one small critique raised below, the statistical analyses were appropriate and valid. The level of specificity in the methods section also makes clear exactly how to reproduce the findings even without access to code. Given that understanding anti-democratic sentiment in democratic countries is a research area in its infancy, this paper has the potential to be quite citable.

That said, there are a few things that I think the authors should address in order to make the paper the strongest it can be. First, while I am sympathetic to the ideology as motivated social cognition framework, there is always the counter-hypothesis that it is ideological extremity, either in addition to or instead of conservatism, that drives anti-democratic sentiment. There is enough literature on ideological extremity's correlates to at least merit testing with a quadratic term for conservatism. It is up to the authors whether they would want to include it in the main models or relegate these to the Appendix.

Second, it is not clear that one of the outcome variables the authors use - perceptions of the U.S. being in a crisis - is inherently a variable about support for democracy. Of course, people who think America's way of life is under attack may support anti-democratic measures to preserve their way of life. However, Americans who favor democratic institutions can still think existing institutions are under attack as a result of numerous efforts to restrict voting, for example. The authors should justify using this as an outcome variable.

Third, the authors correctly point out in the discussion section that the mediation model should not be taken as causal. However, they should go one step further. The mediation model itself is not even necessarily in the correct direction of causation. The Dual Process Model of ideology and prejudice (Duckitt & Sibley, 2017), for example, would suggest that RWA and SDO are precursors, not outcomes, of conservatism. Rather, this mediation model is simply the best way of showing what happens to the association between conservatism and other outcomes when controlling for various motivational processes associated with conservatism. The authors should make the more specific point, then, that this model is mainly for illustrative purposes rather than drawing any conclusions on causal direction.

Fourth, the analyses which assess how Democrats and Republicans who support vs. oppose the insurrection (Figure 2) or support vs. oppose anti-democratic candidates (Figure 3) compare on conservatism and associated motivational processes use simple difference sin means. However, couldn't support for the insurrection be modeled as a dependent variable using a similar regression as that for prior variables? Additionally, since willingness to vote for an anti-democratic candidate is already modeled earlier, what does Figure 3 add that cannot be gleaned by prior analyses?

All in all, though, this was smart work that makes important conclusions. It was also a pleasure to read. The issues with this paper are completely surmountable.

References:
Duckitt, J., & Sibley, C. G. (2017). The dual process motivational model of ideology and prejudice. In C. G. Sibley & F. K. Barlow (Eds.), The Cambridge handbook of the psychology of prejudice (pp. 188–221). Cambridge University Press. https://doi.org/10.1017/9781316161579.009

January 26, 2024

Ref.: COMMSPSYCHOL-23-0303

Authors' response to reviews for "A Political Psychological Analysis of Anti-Democratic Tendencies in the United States."

We thank the reviewers and the editor for their extremely helpful and constructive comments and suggestions. We have now revised the manuscript to address each of the comments enumerated below.

REVIEWERS' COMMENTS

Reviewer #2

(1) The aim of this paper is to analyse anti-democratic tendencies from a psychological perspective in a representative sample of US adults. The findings are consistent with the previous studies and help disentangle the role of ideological differences. Both Democrats and Republicans who supported the attack on the US Capitol had higher RWA and SDO scores than those who did not. Moreover, the PSJ, which is associated with the status quo, is a significant mediator on all issues related to system stability.

The authors clearly explain the limitations of this survey, but they discuss very deeply the psychological implication underlying the transformation of the political scenario, i.e. the simple dichotomy.

The paper is very well written and the contribution to the field very relevant. The analyses [are] explained accurately. It is ready to be published.

Response: We are extremely grateful to this reviewer for sharing this very positive feedback!

Reviewer #3

(2) There is much to like about the paper "A Political Psychological Analysis of Ideological Asymmetries in Anti-Demographic Tendencies in the U.S". The authors explore an important and timely research question, use a nationally representative sample from a politically polarized country, and explore their research questions in a context where anti-democratic attitudes have risen.

Response: We thank the reviewer for the positive feedback and generous comments on our work.

(3) I agree that political conservatism (identity, self-placement), RWA, SDO, and system justification are relevant to understanding anti-democratic attitudes. However, as the paper

stands, it is not entirely clear or fully explained why the authors do. First, the reason for the particular ordering of these variables in the present paper is unclear (I'm not necessarily suggesting that the authors are wrong – more so that the rationale for their ordering is absent from their paper and therefore it's difficult to evaluate). As one example, the Dual Process Model (Duckitt, 2001) positions RWA and SDO as predictors of conservative attitudes and self-placement rather than vice versa. Other research has used measures of RWA, SDO, and system justification as measures of conservatism (e.g. Jost et al., 2003) (for what it's worth, I disagree that these are synonymous with conservatism; see also e.g. Stenner, 2005 – and therefore it makes sense to separate these constructs).

Response: This is an excellent point, and we are grateful to the reviewer for raising this question. Indeed, the DPM model suggests a causal/developmental sequence for ideology (and other social and political attitudes) that implicates a different variable ordering than the one we have used in our mediational analyses. However, in our analysis, we did not attempt to make any causal or developmental assumptions about pro- vs. anti-democratic tendencies. Rather, our analysis aimed to elucidate the possible reasons why liberals and conservatives differ in their pro- and anti-democratic positions; in other words, whether these differences are observed because liberals and conservatives also differ in their levels of RWA, SDO, and PSJ. We recognize that, in the previous version of our submission, the theoretical rationale was not fully spelled out. So, we appreciate the reviewer raising this question because it led us to revise the presentation of these results as follows.

Changes in the manuscript:

a. In the "Mediation analyses" section, we now explicitly state that we are not attempting to test causal assumptions and further elaborate on the theoretical rationale for the ordering of variables in our Structural Equation Models (pp. 14-16).

b. In the "General Discussion" section, we now explicitly acknowledge our inability to draw causal conclusions as a limitation of this study (p. 19).
c. We also conducted additional analyses to explore the DPM Model, with the re-ordering of the variables suggested by the reviewer. The results of this alternative model are now briefly described the results in the "Mediation analyses" section in the main text (p. 16), and we now include the full analyses in the supplementary information.

(4) The paper could benefit from additional rationale for why RWA, SDO, and system justification specifically beyond a single sentence on page 2 (lines 76-77) that says "RWA and SDO are both associated with decreased commitment to basic democratic norms and principles." Adding some information about these constructs would help to appreciate why these are relevant and relate to anti-democratic attitudes, and why they might be positioned as mediators – especially given that this is at odds with some widely accepted models and conceptualizations of RWA, SDO, SJPs and conservatism.

Response: We appreciate the reviewer highlighting this issue. We agree with the recommendation to elaborate further on why RWA, SDO, and PSJ are relevant for understanding pro- vs. anti-democratic tendencies.

Changes in the manuscript:

In the introduction, we now cite evidence from prior research that has explicitly linked RWA and SDO to anti-democratic positions (p. 3) and explicitly state how and why PSJ is expected to be related to support for the democratic status quo (p. 4).

(5) If the authors wish to tackle the asymmetry question, I was surprised to not see an exploration of a potential curvilinear relationship, for instance, to potentially more directly evaluate whether the extremes are more anti-democratic. I was surprised to not see more mention of research that might argue for a curvilinear relationship to situate the research in more broadly (e.g. van Prooijen & Krouwel, 2019; but see also Palaiologou, 2019, Adler, 2018). For example, maybe the extremes are less democratic, but the relationship is stronger on the conservative side than the liberal side, or for particular participants (Democrats, Republicans, those that supported vs. opposed the insurrection).

Response: This is another great point, and we are grateful to the reviewer for bringing the debate between Adler (2018) and Palaiologou (2019) to our attention. We now test the extremism hypothesis directly by including statistical terms for the absolute value of distance from the midpoint of the ideology and strength of party identification scales. Importantly, the inclusion of these variables did not substantively change the picture: we still observe robust ideological asymmetries in all these models.

Changes in the manuscript:

a. We have now added two extra steps to our hierarchical regression models to investigate how ideological extremism and partisan extremism predict the outcome variables, how they affect the other predictors (if at all), and how much additional statistical variability (R^2) is accounted for by including extremity.

2. We conducted supplemental analyses for the Structural Equation Models adjusting for ideological and partisan extremism; these are described in the supplementary materials. Once again, no substantive changes were observed.

(6) Apologies if I'm just missing this, but were the hypotheses pre-registered? Or is it just that the data is available?

Response: Our hypotheses were not pre-registered, because they were derived from previously published works by Jost, including the (2021) *Left & Right* book, which discusses at length the roles of political conservatism, RWA, SDO, and system justification in authoritarian vs. democratic attitudes and behaviors. The second author (Jost) also participated in the survey design. The sample size is large enough to safeguard against "false positives" (i.e., Type I errors).

(7) Were the options for gender female/male? These terms are typically reserved for sex with man/woman typically being used for gender. Were other gender options available?

Response: The survey options listed were only female vs. male, so in the main text and in the supplementary materials we have changed the relevant descriptor from "gender" to "sex," as the reviewer rightly recommended.

(8) Could the authors add the R2 values to the figures? Could the authors include the 95% confidence intervals?

Response: Yes, we have now included the R^2 values in the Structural Equation Model figures and added the 95% standardized confidence intervals to the hierarchical regression tables. We are grateful to the reviewer for these recommendations.

(9) I appreciate the potential value in focusing on participants who approved vs. disapproved of the January 6 insurrection; however, drawing conclusions from samples of 40, 19, or 47 participants has some challenges – even with a representative sample. I would encourage the authors to, at a minimum, remind readers of the limitations of these types of comparisons; and potentially, present these analyses as more descriptive.

Response: We thank the reviewer for raising this point. We agree that it is important to be cautious about drawing conclusions with small (sub)samples and that limitations should be clearly stated in the manuscript. We have now mentioned this limitation in the General Discussion: "However, these findings should be taken with a grain of salt, because they are based on small subsamples of Republicans and (especially) Democrats who approved of the insurrectionists" (p. 19)

Reviewer #4 (Remarks to the Author):

(10) This work is competently done, using a large, nationally representative sample. They incorporate appropriate psychological variables as predictors. While most of the scales examining attitudes toward democracy are not established scales, they are face-valid and the authors, through factor analysis, demonstrate a clear need for separate measures. With the exception of one small critique raised below, the statistical analyses were appropriate and valid. The level of specificity in the methods section also makes clear exactly how to reproduce the findings even without access to code. Given that understanding anti-democratic sentiment in democratic countries is a research area in its infancy, this paper has the potential to be quite citable.

Response: We are grateful to the reviewer for sharing these very encouraging comments.

(11) First, while I am sympathetic to the ideology as motivated social cognition framework, there is always the counter-hypothesis that it is ideological extremity, either in addition to or instead of conservatism, that drives anti-democratic sentiment. There is enough literature on ideological extremity's correlates to at least merit testing with a quadratic term for conservatism. It is up to the authors whether they would want to include it in the main models or relegate these to the Appendix.

Response: This is a great point, and we thank the reviewer for also bringing it to our attention. As detailed in our response to Reviewer 3's point #5 (above), we agree that it is advantageous to confront the extremism hypothesis directly in our analyses.

Changes in the manuscript (also mentioned above):

a. We have now added two extra steps to our hierarchical regression models to investigate how ideological extremism and partisan extremism predict the outcome variables, how they affect the other predictors (if at all), and how much additional statistical variability (R^2) is accounted for by including extremity.

b. We conducted supplemental analyses for the Structural Equation Models adjusting for ideological and partisan extremism; these are described in the supplementary materials. Once again, no substantive changes were observed.

(12) Second, it is not clear that one of the outcome variables the authors use - perceptions of the U.S. being in a crisis - is inherently a variable about support for democracy. Of course, people who think America's way of life is under attack may support anti-democratic measures to preserve their way of life. However, Americans who favor democratic institutions can still think existing institutions are under attack as a result of numerous efforts to restrict voting, for example. The authors should justify using this as an outcome variable.

Response: This is an excellent point, and we were entirely convinced by the reviewer's reasoning. Therefore, we have excluded the variable "Perceptions of a U.S. crisis" from all the analyses carried out in the manuscript and noted (in the Method section) why.

(13) Third, the authors correctly point out in the discussion section that the mediation model should not be taken as causal. However, they should go one step further. The mediation model itself is not even necessarily in the correct direction of causation. The Dual Process Model of ideology and prejudice (Duckitt & Sibley, 2017), for example, would suggest that RWA and SDO are precursors, not outcomes, of conservatism. Rather, this mediation model is simply the best way of showing what happens to the association between conservatism and other outcomes when controlling for various motivational processes associated with conservatism. The authors should make the more specific point, then, that this model is mainly for illustrative purposes rather than drawing any conclusions on causal direction.

Response: Yes, this is the same point raised by Reviewer 3 (see point #3 above), and both reviewers are absolutely correct. Accordingly, we have made three significant changes.

Changes in the manuscript (also noted above):

a. In the "Mediation analyses" section, we now explicitly state that we are not attempting to test causal assumptions and further elaborate on the theoretical rationale for the ordering of variables in our Structural Equation Models (pp. 14-16).

b. In the "General Discussion" section, we now explicitly acknowledge our inability to draw causal conclusions as a limitation of this study (p. 19).

c. We also conducted additional analyses to explore the DPM Model, with the re-ordering of the variables suggested by the reviewer. The results of this alternative model are now briefly described the results in the "Mediation analyses" section in the main text (p. 16), and we now include the full analyses in the supplementary information.

(14) Fourth, the analyses which assess how Democrats and Republicans who support vs. oppose the insurrection (Figure 2) or support vs. oppose anti-democratic candidates (Figure 3) compare on conservatism and associated motivational processes use simple difference sin means. However, couldn't support for the insurrection be modeled as a dependent variable using a similar regression as that for prior variables? Additionally, since willingness to vote for an anti-democratic candidate is already modeled earlier, what does Figure 3 add that cannot be gleaned by prior analyses?

Response: Yes, the reviewer makes a very useful suggestion. The outcome variable is categorical (approval vs. disapproval of the insurrectionists) rather than continuous, but it can still be modeled within a regression framework. We have now done this, as noted below.

Changes in the manuscript:

a. We removed the "Comparisons of respondents who were willing (vs. unwilling) to vote for blatantly anti-democratic candidate" subsection from the manuscript;

b. In the "Analysis of respondents who approved vs. disapproved of the January 6 insurrectionists" section, we have added a new table for the hierarchical regression models carried out (pp. 16-17) and a figure on p. 40.

We thank the editor and reviewers for their excellent comments and observations!

Yours sincerely,

Débora de Oliveira Santos
John T. Jost

1st Mar 24

Dear Ms de Oliveira Santos,

Your manuscript titled "A Political Psychological Analysis of Anti-Democratic Tendencies in the United States" has now been seen by 2 of the 3 original reviewers, whose comments appear below. In light of their advice I am delighted to say that we are happy, in principle, to publish a suitably revised version in Communications Psychology under the open access CC BY license (Creative Commons Attribution v4.0 International License).

We therefore invite you to revise your paper one last time to address the remaining concerns of our reviewers and a list of editorial requests. At the same time we ask that you edit your manuscript to comply with our format requirements and to maximise the accessibility and therefore the impact of your work.

EDITORIAL REQUESTS:

Please review our specific editorial comments and requests regarding your manuscript in the attached "Editorial Requests Table". Please outline your response to each request in the right hand column. Please upload the completed table with your manuscript files as a Related Manuscript file.

If you have any questions or concerns about any of our requests, please do not hesitate to contact me.

SUBMISSION INFORMATION:

In order to accept your paper, we require the files listed at the end of the Editorial Requests Table; the list of required files is also available at https://www.nature.com/documents/commsj-file-checklist.pdf .

OPEN ACCESS:

Communications Psychology is a fully open access journal. Articles are made freely accessible on publication under a CC BY license (Creative Commons Attribution 4.0 International License). This license allows maximum dissemination and re-use of open access materials and is preferred by many research funding bodies.

For further information about article processing charges, open access funding, and advice and support from Nature Research, please visit https://www.nature.com/commspsychol/article-processing-charges

At acceptance, you will be provided with instructions for completing this CC BY license on behalf of all authors. This grants us the necessary permissions to publish your paper. Additionally, you will be asked to declare that all required third party permissions have been obtained, and to provide billing information in order to pay the article-processing charge (APC).

* TRANSPARENT PEER REVIEW: Communications Psychology uses a transparent peer review system. On author request, confidential information and data can be removed from the published reviewer reports and rebuttal letters prior to publication. If you are concerned about the release of confidential data, please let us know specifically what information you would like to have removed. Please note that we cannot incorporate redactions for any other reasons.

* CODE AVAILABILITY: All Communications Psychology manuscripts must include a section titled "Code Availability" at the end of the methods section. We require that the custom analysis code supporting your conclusions is made available in a publicly accessible repository at this stage; please choose a repository that generates a digital object identifier (DOI) for the code; the link to the repository and the DOI must be included in the Code Availability statement. Publication as Supplementary Information will not suffice.

* DATA AVAILABILITY:
All Communications Psychology manuscripts must include a section titled "Data Availability" at the end of the Methods section. More information on this policy, is available in the Editorial Requests Table and at http://www.nature.com/authors/policies/data/data-availability-statements-data-citations.pdf.

Please use the following link to submit the above items:
[Link redacted]
** This url links to your confidential home page and associated information about manuscripts you may have submitted or be reviewing for us. If you wish to forward this email to co-authors, please delete the link to your homepage first **

We hope to hear from you within two weeks; please let us know if you need more time.

Best regards,

Jennifer Bellingtier

Jennifer Bellingtier, PhD
Senior Editor
Communications Psychology

REVIEWERS' EXPERTISE:

Reviewer #3: polarization in US, democratic beliefs
Reviewer #4: democratic beliefs

REVIEWERS' COMMENTS:

Reviewer #3 (Remarks to the Author):

The authors addressed all of the questions I raised in my original review. This is a great paper! I'm looking forward to seeing it in print and citing it.

Reviewer #4 (Remarks to the Author):

Many thanks to the authors for their careful attention to the suggestions the reviewers made. I am

satisfied with the changes that have been made to the analyses and manuscript, and think it is ready for publication.
